# Characterization of the zinc finger proteins ZMYM2 and ZMYM4 as novel B-MYB binding proteins

**DOI:** 10.1038/s41598-020-65443-w

**Published:** 2020-05-21

**Authors:** Hannah Cibis, Abhiruchi Biyanee, Wolfgang Dörner, Henning D. Mootz, Karl-Heinz Klempnauer

**Affiliations:** Institute for Biochemistry, Westfälische-Wilhelms-Universität, D-48149 Münster, Germany

**Keywords:** Oncogenes, Mass spectrometry, Checkpoints, Transcription, Sumoylation, Protein-protein interaction networks

## Abstract

B-MYB, a highly conserved member of the MYB transcription factor family, is expressed ubiquitously in proliferating cells and plays key roles in important cell cycle-related processes, such as control of G2/M-phase transcription, cytokinesis, G1/S-phase progression and DNA-damage reponse. Deregulation of B-MYB function is characteristic of several types of tumor cells, underlining its oncogenic potential. To gain a better understanding of the functions of B-MYB we have employed affinity purification coupled to mass spectrometry to discover novel B-MYB interacting proteins. Here we have identified the zinc-finger proteins ZMYM2 and ZMYM4 as novel B-MYB binding proteins. ZMYM4 is a poorly studied protein whose initial characterization reported here shows that it is highly SUMOylated and that its interaction with B-MYB is stimulated upon induction of DNA damage. Unlike knockdown of B-MYB, which causes G2/M arrest and defective cytokinesis in HEK293 cells, knockdown of ZMYM2 or ZMYM4 have no obvious effects on the cell cycle of these cells. By contrast, knockdown of ZMYM2 strongly impaired the G1/S-phase progression of HepG2 cells, suggesting that ZMYM2, like B-MYB, is required for entry into S-phase in these cells. Overall, our work identifies two novel B-MYB binding partners with possible functions in the DNA-damage response and the G1/S-transition.

## Introduction

The highly conserved MYB proto-oncogene family member B-MYB is ubiquitously expressed in proliferating cells where it acts as an essential cell cycle-regulated transcription factor^1,2^. Mammalian B-MYB, like its *Drosophila melanogaster* homolog, are important interaction partners of the MYB-MuvB/DREAM complex, an evolutionarily conserved multiprotein complex that controls the transcription of genes that are relevant for mitosis^3,4^. In resting cells, the MuvB core complex, consisting of Lin-9, Lin-37, Lin-54, Lin-52 and RBBP4, associates with E2F4 and either p130 or p107 to form the DREAM complex, which acts as a repressor of E2F target genes. In S-phase, the MuvB core complex dissociates from E2F4/p130/p107 and recruits B-MYB to form the MYB-MuvB complex, which is then targeted to the promoters of genes required for the G2/M transition and mitosis^5–11^. B-MYB activity itself is highly regulated during the cell cycle by transcriptional and post-transcriptional mechanisms^12–17^. Notably, a stepwise phosphorylation mechanism of B-MYB has been described, which involves sequential phosphorylations mediated by cyclin-dependent kinase (Cdk) and Polo-like kinase 1 (Plk1) and, together with Pin1-facilitated peptidyl-prolyl *cis*/*trans* isomerization, triggers conformational changes of B-MYB to finally allow it to stimulate transcription of its target genes^18^.

In addition to its role as a cell cycle regulated transcription factor B-MYB has also “non-transcriptional” functions in proliferating cells. During mitosis, B-MYB interacts with the MYB-Clafi complex and thereby participates in the formation of the mitotic spindle^19^. B-MYB also stimulates G1/S transition in a manner that is independent of its sequence-specific DNA-binding activity and affects the DNA-replication program, further highlighting the complex manner of cell cycle regulation by B-MYB^20,21^. Recent findings have implicated B-MYB also in the DNA damage response. Disruption of *B-MYB* in chicken DT40 cells reduces their survival when treated with DNA damaging agents^22^. Furthermore, B-MYB has been implicated in the recovery from a cell cycle arrest induced by DNA-damage^23^. UV irradiation-induced cell cycle arrest leads to a switch of B-MYB from Cyclin/Cdk-dependent to Jnk- and p38 kinase-dependent phosphorylation^24,25^. Finally, our recent work has shown that B-Myb is recruited transiently to DNA double strand breaks (DSBs) by interacting with the Mre11-Rad50-Nbs1 (MRN) complex^26^.

In the work reported here we have employed affinity-purification of a GFP-B-MYB fusion protein expressed in HEK293T cells in conjunction with mass spectrometry to explore the B-MYB interacting proteome and to better understand the complex functions of B-MYB. We have identified and characterized the zinc finger proteins ZMYM4 and ZMYM2 as novel B-MYB binding factors.

## Results

### Identification of zinc finger MYM-type protein 4 (ZMYM4) as a novel B-MYB binding protein

Extracts of HEK293T cells stably expressing a GFP/B-MYB fusion protein were incubated with GFP-trap beads, followed by digestion of the bound proteins with trypsin and mass spectrometric analysis of the resulting peptides. This led to a list of proteins detected in three independent experiments in samples derived from GFP/B-MYB expressing cells but absent from samples derived form cells expressing only GFP (Supplementary Table S1). Complete lists of all proteins detected in these experiments are shown in Supplementary Tables S3 to S5. All members of the MuvB core complex (LIN9, RBBP4, LIN54, LIN37 and LIN52) were present in the B-MYB specific samples, demonstrating the reliability of the approach. In addition, several novel proteins were identified in the B-MYB specific samples. Based on their known functions and subcellular localizations, several interactions with proteins localized in mitochondria, the golgi apparatus, or other cytoplasmic vesicles were considered as likely artifacts, probably caused by the preparation of cell extract in buffer containing a membrane-disrupting detergent. For example, P5CS (delta-1-pyrroline-5-carboxylate synthase) is located in the mitochondrial matrix where it is involved in the biochemical pathway of L-proline synthesis. Using CRAPome, a database of common contaminants in affinity-purification mass spectrometry (AP-MS/MS) experiments^27^, we also excluded proteins frequently found in affinity-purification experiments from further analysis. This left the nuclear protein ZMYM4 (zinc finger MYM-type protein 4) as the most promising candidate of a novel B-MYB binding protein.

**Figure 1:**
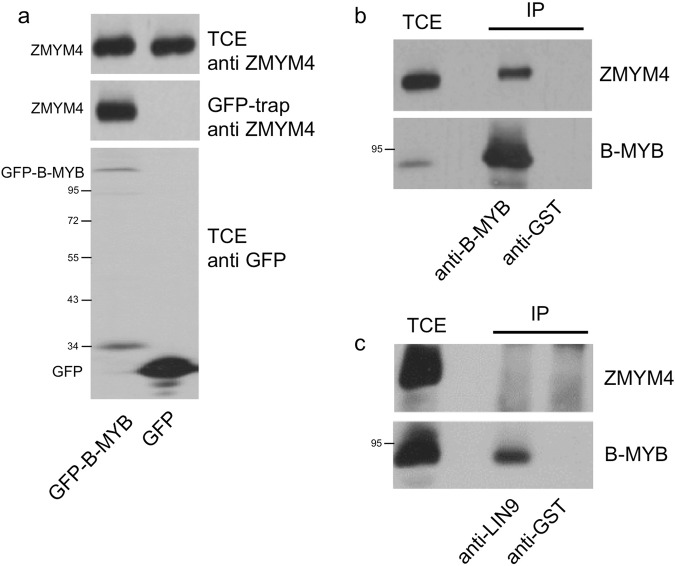
B-MYB interacts with ZMYM4. (**a**) Total extracts of Hek293T cells expressing GFP or GFP-B-MYB were analyzed by GFP-trap. Samples of the total extract (TCE) and the proteins bound to GFP-trap beads were analyzed by Western blotting with ZMYM4- and GFP-specific antibodies. (**b,c**) Total extracts of untransfected HEK293T cells were immunoprecipitated by antibodies against B-MYB, LIN9 or GST, followed by Western blotting of aliquots of the total extract and the immunoprecipitates (IP) with antibodies against ZMYM4 and B-MYB.

We verified the interaction of B-MYB and ZMYM4 by Western blotting of GFP-trap samples with antibodies against ZMYM4 (Fig. 1a). To confirm the interaction of B-MYB and ZMYM4 on the endogenous expression level, we immunoprecipitated B-MYB from extracts of untransfected HEK293T cells followed by Western blotting with antibodies against ZMYM4 (Fig. 1b). This showed that ZMYM4 was co-precipitated by B-MYB specific antibodies but not by unrelated control antibodies.

To investigate whether ZMYM4 is recruited via B-MYB to the MYB-MuvB complex we co-precipitated cell extracts with antibodies against LIN9, the direct interaction partner of B-MYB in this complex^7,8^. As shown in Fig. 1c, ZMYM4 was not detected in the LIN9 precipitate whereas B-MYB was readily co-precipitated. Thus, we currently have no evidence that ZMYM4 is recruited to the MYB-MuvB complex, at least under normal growth conditions.

### ZMYM4 interacts with the central part of B-MYB

**Figure 2:**
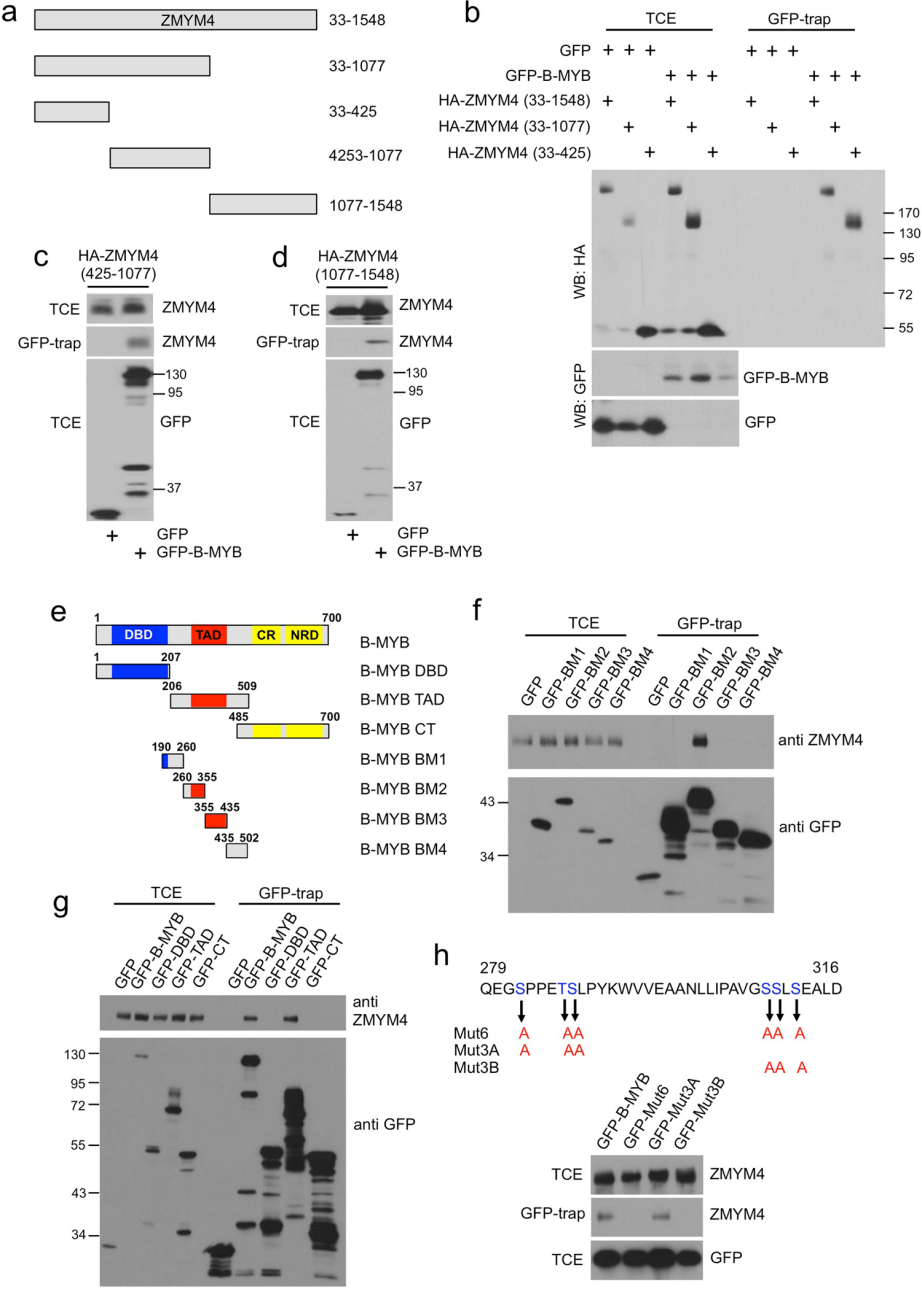
Mapping the binding sites in ZMYM4 and B-MYB. (**a**) Schematic illustration of HA-tagged ZMYM4 deletion constructs. Numbers refer to amino acids. (**b)** Extracts of HEK293T cells transfected with combinations of expression vectors (top) were analyzed by GFP-trap. Aliquots of the cell extracts (TCE) and bound proteins (GFP-trap) were analyzed by Western blotting with antibodies against the HA-tag or GFP. (**c,d**) Extracts of HEK293T cells transfected with expression vectors for GFP, GFP-B-MYB and HA-ZMYM4(425–1077) (**c**) or HA-ZMYM4(1077–1546) (**d**) were analyzed as described for panel b. (**e)** Schematic illustration of the domains of B-Myb (DBD: DNA binding domain; TAD: transactivation domain; CR: conserved region; NRD: negative regulatory domain) and of YFP/B-Myb deletion constructs. The numbers refer to amino acids. (**f–h)** HEK293T cells were transfected with expression vectors for GFP-B-MYB constructs as indicated at the top. Cell extracts were incubated with GFP-trap beads and bound endogenous ZMYM4 was detected with antibodies against ZMYM4. In panel h, expression vectors for GFP-fused full-length B-MYB carrying the amino acid substitutions indicated in the partial B-MYB amino acid sequence at the top were used.

We used HA-tagged deletion mutants of ZMYM4 to identify the domains mediating the interaction with B-MYB (Fig. 2a). Co-expression with GFP/B-MYB or GFP, followed by GFP-trap showed that the full-length protein as well as ZMYM4/33-1077 interacted with B-MYB while the N-terminal part of ZMYM4 (ZMYM4/33-425) did not interact (Fig. 2b). Further mapping experiments showed that the middle part of ZMYM4 (ZMYM4/425-1077) interacted well with B-MYB (Fig. 2c) while the C-terminal part (ZMYM4/1077-1548) interacted poorly (Fig. 2d). Overall, these experiments showed that the interaction with B-MYB is mainly mediated by the middle part of ZMYM4.

To map the ZMYM4 binding region within B-MYB we used a panel of GFP/B-MYB deletion mutants described before (Fig. 2e)^26^. GFP-trap experiments first narrowed down the binding region for ZMYM4 to the central part of B-MYB between amino acids 206 and 509 (Fig. 2g). This region was then further dissected to show that ZMYM4 binds to amino acids 260–355 of B-MYB (Fig. 2f). During the characterization of the interaction between B-MYB and NBS1 we recently identified DNA-damage induced phosphorylations at several amino acids in this part of B-MYB and mutated several serine and threonine residues to alanine^26^. These mutants were also examined for their ability to interact with ZMYM4. GFP-trap experiments showed that mutant 3B, which contains amino acid replacements of serine 306, 307 and 309 by alanine, disrupts the binding of ZMYM4 to B-MYB (Fig. 2h).

**Figure 3:**
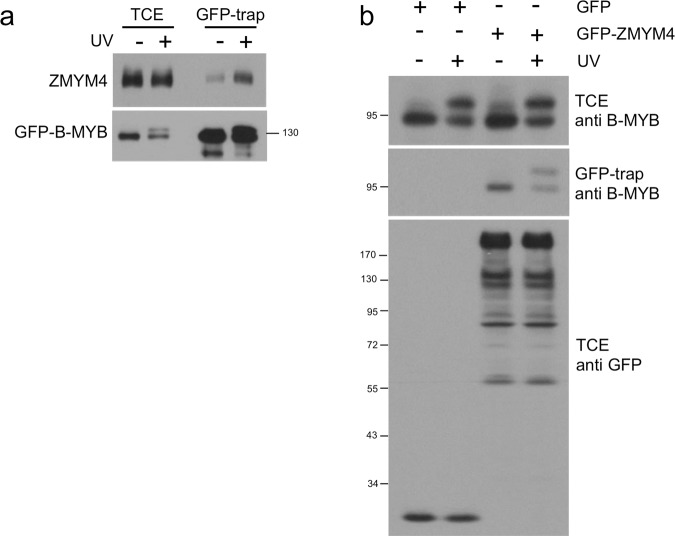
ZMYM4 shows increased binding to B-MYB after UV-irradiation and interferes with the transactivation potential of B-MYB. (**a**) HEK293T were incubated with 10 μM BrdU, starting 24 h before transfection, and irradiated with UV-C for 6 sec one hour before they were harvested 24 h after transfection. As control, cells were not irradiated. Total cell extracts were then analyzed by GFP-trap and Western blotting with the indicated antibodies. (**b)** HEK293T were pre-incubated with BrdU (as described above) and transfected with expression vectors for B-MYB, GFP-ZMYM4 and GFP, as shown at the top. UV-C irradiation and GFP-trap analysis was performed as described for panel a.

Previously, we have demonstrated the phosphorylation of these residues when cells preincubated with bromo-deoxy uridine (BrdU) were irradiated with UV-C, leading to the appearance of a form of B-MYB with lower mobility in SDS-polyacrylamide gels^26^. We therefore investigated whether the binding of ZMYM4 to B-MYB is affected under such conditions of DNA-damage. As shown by GFP-trap, binding of endogenous ZMYM4 to GFP-B-MYB was increased by UV-irradiation (Fig. 3a). Densitometric analysis of the protein bands from several replicate experiments showed an average 4.1 fold (standard deviation +/− 1.3 fold) increase of binding of ZMYM4 to GFP-B-MYB after UV irradiation. The UV-induced mobility shift of B-MYB was also clearly visible in this experiment. We also performed a complementary experiment in which we monitored the interaction of endogenous B-MYB with GFP-ZMYM4. This experiment showed no increased binding of the phosphorylated (i.e. up-shifted) form of B-MYB to ZMYM4, suggesting that the UV-induced phosphorylation of the serine residues was not responsible for the stronger binding of ZMYM4 upon induction of DNA-damage (Fig. 3b). Thus, it appears likely that an unknown modification of B-MYB, ZMYM4 or an unknown protein causes the stronger interaction of ZMYM4 with B-MYB in Fig. 3a. In any case, these observations provide the first evidence for a possible role of ZMYM4 in the DNA-damage response.

### ZMYM4 is modified by SUMO conjugation

**Figure 4:**
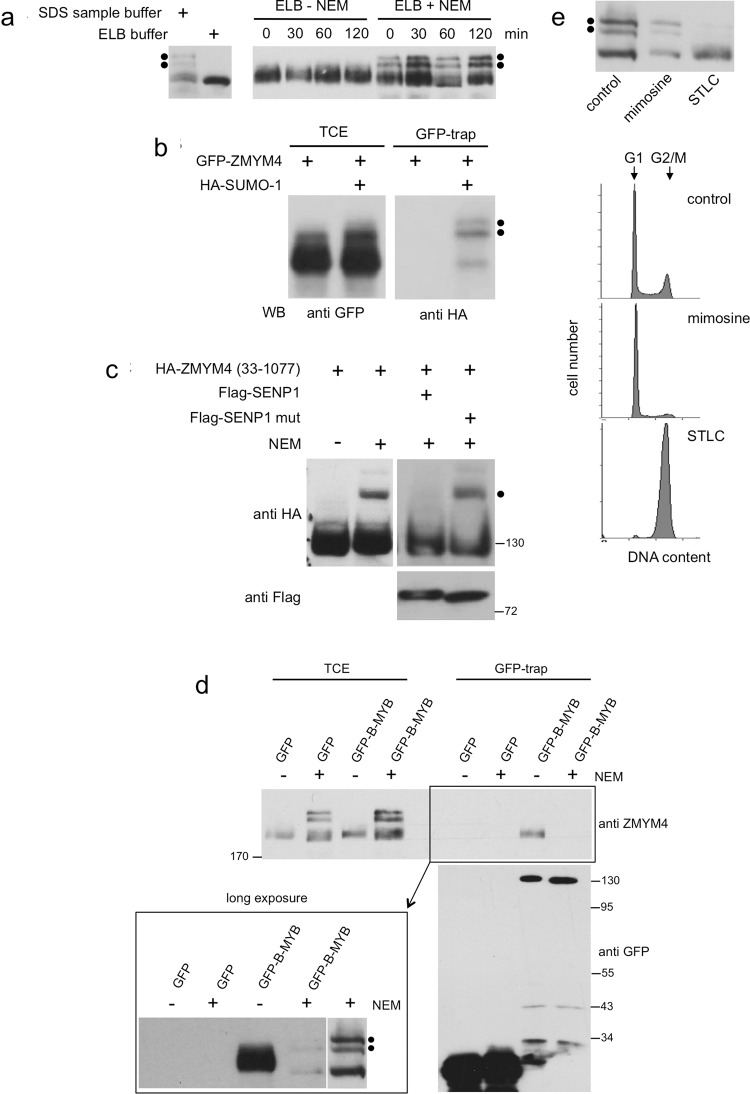
SUMOylation of ZMYM4. (**a**) High molecular weight forms of ZMYM4 (marked with black dots) are visible under different buffer conditions. (**b)** HEK293T cells were transfected with expression vectors for GFP-ZMYM4 and HA-SUMO-1, as indicated. Total cell extracts were analyzed by Western blotting with antibodies against GFP. Part of the cell extract was incubated with GFP-trap beads and bound proteins were analyzed by HA-specific antibodies. (**c**) HEK293T cells were transfected as indicated at the top. Total cell extracts were prepared with or without NEM and analyzed by Western blotting with antibodies against the HA- or Flag-tag. The black dot marks a band with lower electrophoretic mobility. (**d)** Extracts of HEK293T cells transfected with expression vectors for GFP or GFP-B-MYB and prepared without or with NEM were bound to GFP-trap beads. Samples of the cell extract and the bound proteins were analyzed by Western blotting for ZMYM4, GFP and GFP-B-MYB. The lower left part of the figure shows a longer exposure of the last four lanes juxtaposed to a shorter exposure of a lane from the same gel containing NEM-treated total cell extract. Note that in the sample of GFP-B-MYB bound protein only one of the modified forms of ZMYM4 is present. (**e)** HepG2 cells were cultivated in normal growth medium or in growth medium supplemented with mimosine or STLC. Total cell extracts were then analyzed by Western blotting with antibodies against ZMYM4. The cells were also analyzed by flow cytometry to assess their cell cycle status.

Western blotting showed additional higher molecular weight forms of ZMYM4 when the cells were lysed directly in SDS-sample buffer rather than in buffer containing only NP40 as detergent (Fig. 4a). This indicated that the higher molecular weight forms of ZMYM4 might be due to covalent modifications of the proteins that are labile in cell extracts. Because of the significant increase in apparent molecular weight caused by the modification and its instability we reasoned that the higher molecular weight bands might represent SUMOylated forms of ZMYM4. To address this, we prepared cell extract in the presence or absence of N-ethylmaleimide (NEM), a potent inhibitor of cysteine-proteases that is known to inhibit the activity of the SUMO-specific proteases^28^. We observed that the higher molecular weight forms of ZMYM4 were stable in the presence of NEM but not in its absence (Fig. 4a). To further demonstrate that the slower migrating bands correspond to SUMO-modified ZMYM4 we transfected HEK293T cells with GFP/ZMYM4 and HA-tagged SUMO1. Total cell extracts were prepared in the presence of NEM and analyzed by GFP-trap and Western blotting. This demonstrated that higher molecular weight forms of GFP/ZMYM4 reacted with HA-specific antibodies (Fig. 4b), consistent with SUMO-conjugation of ZMYM4. In a further experiment we co-expressed HA-ZMYM4/33-1077 together with Flag-tagged wt-SENP1 or a catalytically inactive SENP1 mutant. Western blotting of total cell extract prepared in the presence of NEM showed a higher molecular weight form of the truncated ZMYM4 that was abolished by wt-SENP1 but maintained when mutant SENP1 was co-expressed (Fig. 4c). Taken together, these experiments showed that ZMYM4 is SUMOylated, presumably at two sites. Moreover, the experiment suggested that at least one of the SUMOylation sites is located between amino acids 33 to 1077 of ZMYM4.

We wondered whether SUMOylation of ZMYM4 affects its binding to B-MYB. This was not evident from previous co-precipitation experiments because they were carried out in the absence of NEM where ZMYM4 was de-SUMOylated. We performed a GFP-trap experiment using cell extract of HEK293T cells prepared with NEM to block de-SUMOylation, or without NEM as control (Fig. 4d). Surprisingly, in the presence of NEM the binding was almost completely lost. Since the ZMYM4 binding region in B-MYB does not contain nearby cysteine residues, the loss of binding in the presence of NEM may be due to the disruption of the zinc-fingers of ZMYM4 caused by covalent modification of critical cysteine residues and concomitant structural changes in ZMYM4. However, longer exposure of the Western blot of Fig. 4d revealed weak binding of ZMYM4 to B-MYB. Interestingly, the slowest migrating of the modified forms of ZMYM4 was under-represented in the bound fraction, suggesting that the modification responsible for the slowest migrating ZMYM4 species interferes with binding to B-MYB.

As a first step to understand the physiological role of the SUMOylation of ZMYM4 we investigated whether the extent of SUMOylation of ZMYM4 varies with the state of the cells. We used inhibitors to arrest the cells at specific points in the cell cycle and analyzed the mobility of ZMYM4 by Western blotting. Treatment with mimosine, an inhibitor of DNA-replication that arrests cells at the G1/S-boundary and within S-phase^29^, had no significant effect on the extent of SUMOylation in HepG2 cells. Treatment with the kinesin Eg5 inhibitor STLC (S-Trityl-L-cystein), which arrests the cells in mitosis^30^, showed a clear reduction of the amount of the higher molecular weight forms (Fig. 4e). Such variations were also seen in HEK293T cells treated with mimosine or STLC (data not shown), providing the first evidence that the SUMOylation of ZMYM4 may be regulated during the cell cycle.

### The ZMYM4-related zinc-finger protein ZMYM2 interacts with B-Myb

**Figure 5:**
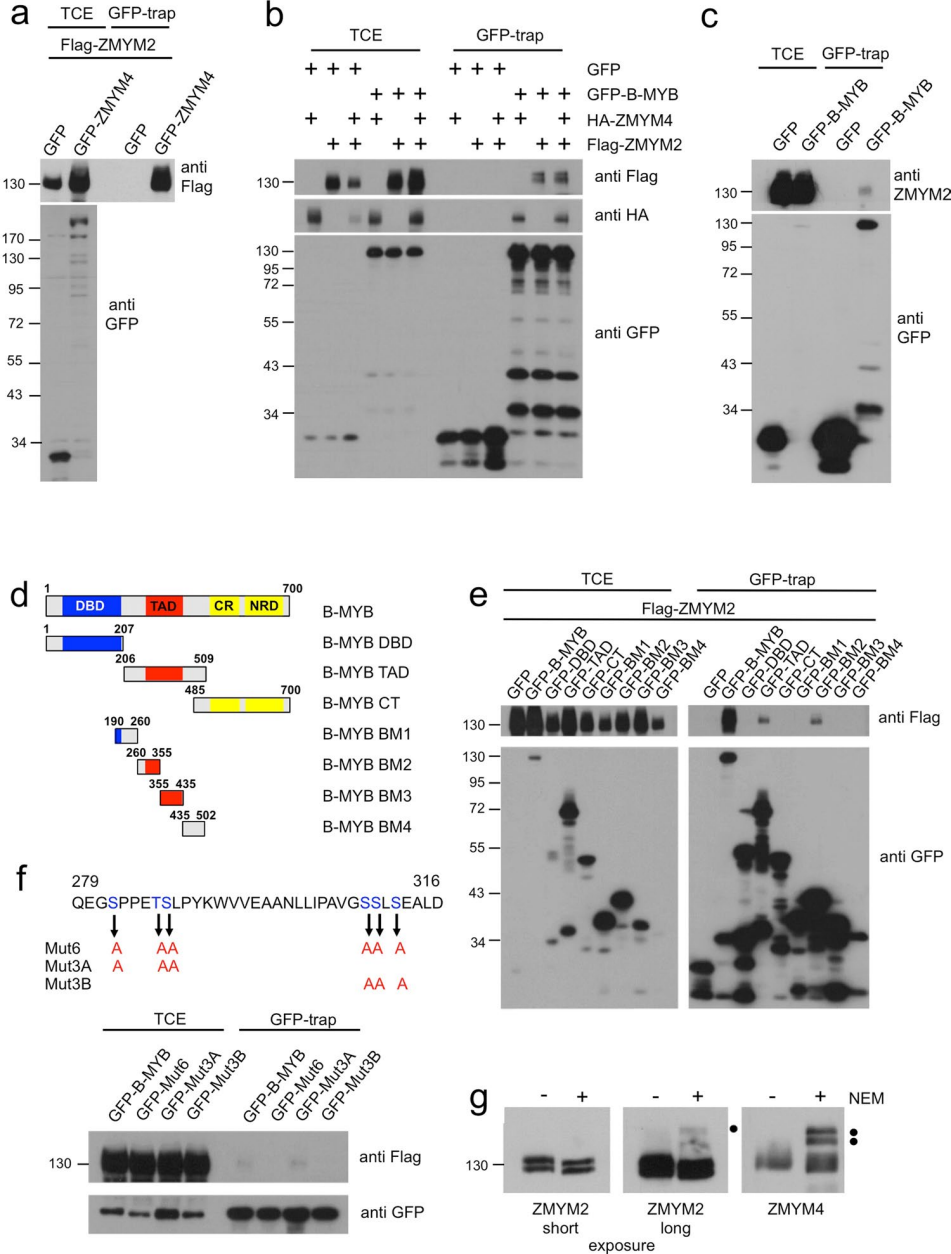
Characterization of the interaction between ZMYM2 and B-MYB. (**a**) QT6 cells were transfected with expression vectors for Flag-ZMYM2, GFP and GFP-ZMYM4. Cell extracts were subjected to GFP-trap and input and bound proteins were analyzed by Western blotting with Flag- and GFP-specific antibodies. (**b)** Extracts of QT6 cells transfected as shown at the top were examined by GFP-trap. Aliquots of total cell extracts and bound fractions were analyzed by Western blotting using the indicated antibodies. **c**. Extracts of HEK293 cells stably expressing GFP or GFP-B-MYB were analyzed by GFP-trap. ZMYM2- and GFP-specific antibodies were used to display GFP-proteins and endogenous ZMYM2. (**d)** Schematic illustration of the domains of B-Myb and of GFP/B-Myb deletion constructs. (**e)** QT6 cells were transfected with expression vectors for GFP-B-MYB constructs and Flag-ZMYM2, as indicated at the top. Cell extracts were incubated with GFP-trap beads and bound endogenous ZMYM2 was detected with antibodies against the Flag-tag. (**f)** HEK293T cells were transfected with expression vectors for GFP-fused full-length B-MYB carrying the amino acid substitutions indicated in the partial B-MYB amino acid sequence at the top. Total cell extract and GFP-trap samples were analyzed by Western blotting with antibodies against GFP and endogenous ZMYM2. (**g)** HEK293T cell extracts prepared without or with NEM were analyzed by Western blotting with antibodies against ZMYM2 and ZMYM4. Black dots mark bands with slower electrophoretic mobility.

To further explore the function of ZMYM4 we investigated its interaction partners by generating GFP-ZMYM4 expressing HEK293T cells and analyzing them by GFP-trap and mass spectrometry, using cells expressing only GFP as control. Complete lists of all proteins detected in these experiments are shown in Supplementary Tables S4 to S6. One of the proteins with the highest score in three independent experiments was ZMYM2 (Supplementary Table S2). Complete lists of all proteins detected in these experiments are shown in Supplementary Tables S6 to S8. We confirmed the interaction of ZMYM4 and ZMYM2 by a GFP-trap experiment (Fig. 5a). Given the interaction of ZMYM4 with B-MYB we wondered whether ZMYM2 is recruited to B-MYB via ZMYM4. To address this possibility we co-transfected QT6 fibroblasts with expression vectors for ZMYM4 and ZMYM2, alone or in combination, and GFP-B-MYB or GFP. GFP-trap analysis showed that ZMYM2 was co-precipitated with B-MYB irrespective of the presence or absence of ZMYM4 (Fig. 5b), suggesting that ZMYM2 is also a B-MYB binding protein. We confirmed the interaction of endogenous ZMYM2 and B-MYB by GFP-trap using HEK293T cells expressing GFP/B-MYB (Fig. 5c). The interaction of endogenous ZMYM2 with GFP/B-MYB was much weaker than the interaction with ZMYM4 in an equivalent experiment (Fig. 1a), which might explain why ZMYM2 was not observed as a potential B-MYB binding protein in the initial mass spectrometry experiments.

Mapping the ZMYM2 interaction site within B-MYB showed that ZMYM2 also binds to the central part of B-MYB (Fig. 5d,e). Furthermore, the interaction of B-MYB and ZMYM2 was disrupted in the B-MYB mutant Mut3B, suggesting that ZMYM4 and ZMYM2 bind to B-MYB in similar manner, possibly sharing the same binding site in B-MYB (Fig. 5f). A comparison of the SUMOylation of ZMYM2 and ZMYM4 in cell extracts in the presence of NEM showed that the fraction of SUMOylated ZMYM2 in unsynchronized HEK293T cells is much lower than the fraction of SUMOylated ZMYM4 (Fig. 5g).

### ZMYM2 is required for the G1/S-transition in HepG2 cells

**Figure 6:**
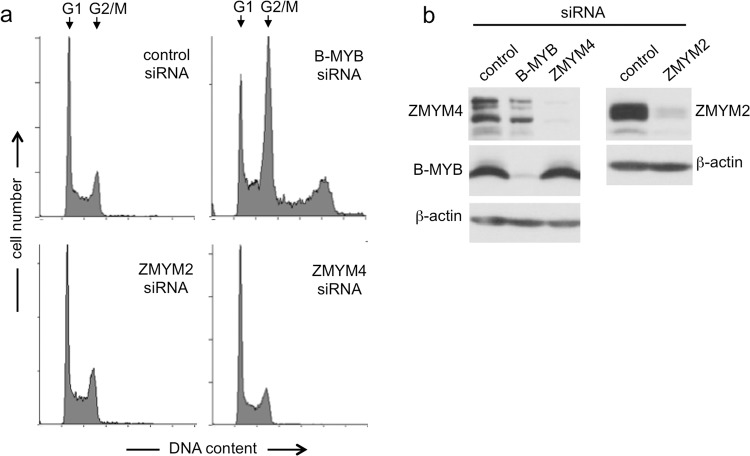
Cell cycle distribution after knockdown of B-MYB, ZMYM2 and ZMYM4. HEK293 cells were transfected with with control-siRNA, B-Myb-siRNA, ZMYM2-siRNA3 and ZMYM4-siRNA2. Cells were analyzed after 72 h by flow cytometry (**a**) and Western blotting (**b**).

B-MYB has been implicated in cell cycle progression at two steps, namely at the G1/S- and the G2/M-transition. In contrast to the G2/M-transition, where B-MYB acts as a part of the MYB-MuvB complex, the role of B-MYB at the G1/S-transition is less well studied^20^. We performed knockdown experiments to investigate whether ZMYM4 or ZMYM2 are also involved in these cell cycle transitions. Knockdown of B-MYB in HEK293 cells increased the abundance of G2/M-cells, reflecting the crucial role of B-MYB at the G2/M-transition. In addition, B-MYB knockdown caused accumulation of cells with a higher DNA content due failure of a fraction of the cells to complete cytokinesis as reported before (Fig. 6)^18,19^. Knockdown on ZMYM4 or ZMYM2 in HEK293 cells (Fig. 6b) did not induce similar changes of the cell cycle profile, suggesting that ZMYM4 and ZMYM2 are not essential for the G2/M transition or cytokinesis (Fig. 6a).

**Figure 7:**
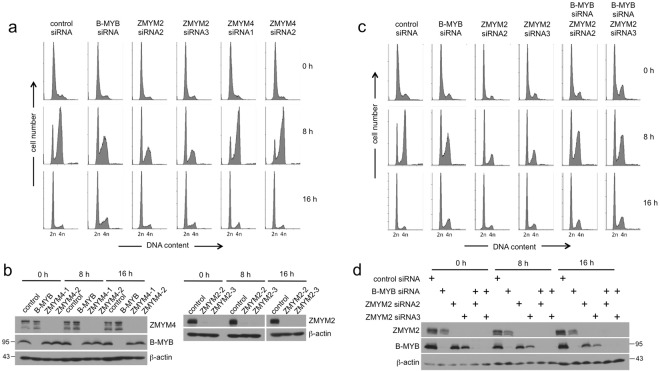
Effect of knockdown of B-MYB, ZMYM2 and ZMYM4 on the G1/S-transition in HepG2 cells. (**a,b**). HepG2 cells were transfected with with the siRNA indicated at the top. Cells were arrested in the G1-phase by addition of 4 mM thymidine 48 h after siRNA transfection. 20 h after addition of thymidine cells were released into S-phase by washing with medium without thymidine. The cells were then analyzed by flow cytometry (**a**) and Western blotting (**b**) immediately or after incubation for 8 or 16 h. (**c,d)** HepG2 cells were transfected with with the siRNA indicated at the top. Thymidine-block and -release were performed as described above. The cells were then analyzed by flow cytometry (**c**) and Western blotting (**d**) immediately or after incubation for 8 or 16 h.

In HepG2 cells, knockdown of B-MYB has been shown to lead to a defect of the G1/S-phase progression^20^. We confirmed this by arresting the cells at the G1/S-boundary with a thymidine block and subsequently releasing them into S-phase in thymidine-free medium (Fig. 7a). Control siRNA-treated HepG2 cells progressed to S-phase within 8 hours whereas only a fraction of the B-MYB knockdown cells entered S-phase. Knockdown of ZMYM2 revealed an even more pronounced disruption of S-phase entry while knockdown of ZMYM4 had no effect (Fig. 7a). This indicated that ZMYM2, like B-MYB, is required for the G1/S-transition in HepG2 cells. The knockdown efficiency was confirmed by Western blotting (Fig. 7b). We confirmed the defect in S-phase entry upon ZMYM2 knockdown also by a ^3^H-thymidine labeling experiment, which showed that the DNA-synthesis activity of the cell population was significantly reduced after knockdown of ZMYM2 (Supplementary Fig. S1).

Since the knockdown of B-MYB as well as of ZMYM2 impaired the G1/S-transition in HepG2 cells we wondered whether or not both proteins act independently of each other to facilitate S-phase entry. To investigate this we examined S-phase entry in HepG2 cells after single knockdown of B-MYB or ZMYM2 and after simultaneous knockdown of B-MYB and ZMYM2 (Fig. 7c). The knockdown of B-MYB and ZMYM2 was confirmed by Western blotting (Fig. 7b). As before, silencing of B-MYB or of ZMYM2 reduced the fraction of cells entering S-phase with a particularly strong reduction in case of the ZMYM2 knockdown. Surprisingly, when ZMYM2 and B-MYB were silenced together the fraction of cells progressing into S-phase was increased to the level seen when only B-MYB was silenced (Fig. 7c). This showed that the strong inhibition of S-phase entry upon silencing of ZMYM2 was relieved when B-MYB was absent. This is difficult to reconcile with a model in which B-MYB and ZMYM2 facilitate progression into S-Phase independently of each other, but rather supports some kind of dependency in the action of B-MYB and ZMYM2 at the G1/S-boundary. The molecular details of this dependency are currently unknown and need to be addressed in future studies.

**Figure 8:**
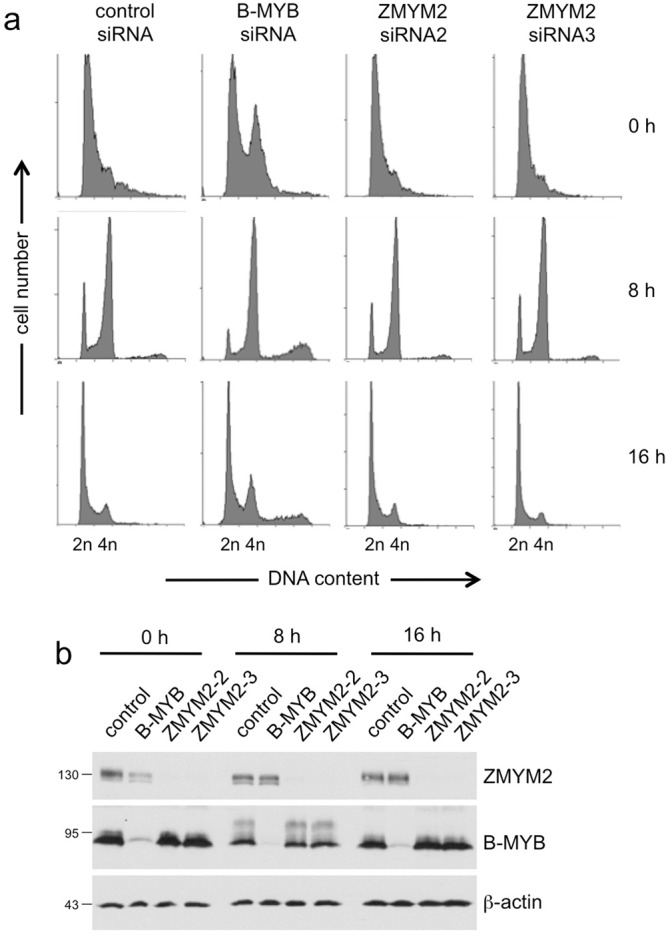
Effect of knockdown of B-MYB and ZMYM2 on the G1/S-transition in HEK293 cells. HEK293 cells were transfected with with the siRNAs indicated at the top. Cells were arrested in the G1-phase by addition of 4 mM thymidine 24 h after siRNA transfection. 20 h later cells were released into S-phase by washing with medium without thymidine. The cells were then analyzed by flow cytometry (**a**) and Western blotting (**b**) immediately after release of the mimosine-block or after further incubation for 8 and 16 h.

Finally, we wondered whether the progression into S-phase was independent of ZMYM2 in HEK293 cells because they did not show an obvious alteration of their cell cycle profile upon ZMYM2 knockdown (Fig. 6). We therefore transfected HEK293 with siRNAs specific for B-MYB and ZMYM2 and arrested them by a thymidine block (Fig. 8a). The knockdown of B-MYB and ZMYM2 was confirmed by Western blotting (Fig. 8b). The control siRNA-arrested cells form an asymmetric peak of G1 cells that has a distinct shoulder towards higher DNA content because the thymidine block also arrests cells that are within S-phase. Nevertheless, it was apparent that most of the control siRNA-treated cells have progressed to the G2-phase within 8 hours after release from the thymidine block. B-MYB siRNA-treated and thymidine-arrested HEK293 cells showed additional to the G1 peak a less prominent peak of G2 cells which is explained by delayed G2/M progression due to the lack of B-MYB expression. However, it is apparent that most of the cells arrested at the G1/S-boundary had also progressed through S-phase during 8 hours after removal of thymidine. As explained before, a fraction of the cells failed to perform cytokinesis, resulting in a small peak with higher DNA content. ZMYM2-silenced HEK293 cells, in contrast to HepG2 cells (Fig. 7), showed no defect of the G1/S-transition and progression to the G2 phase. Together, these findings indicated that neither B-MYB nor ZMYM2 are required for the G1/S-transition in HEK293 cells. Unlike HepG2 cells, HEK293 cells exhibit a defective G1/S-checkpoint caused by the expression the adenovirus E1A protein. E1A sequesters the retinoblastoma protein and allows transcription factor E2F to be active independently of cyclin/Cdk-induced phosphorylation, thereby inactivating the G1/S-checkpoint^31,32^. Our data therefore raise the possibility that the ability of B-MYB and ZMYM2 to promote S-phase entry is linked to a functional G1/S-checkpoint.

## Discussion

To gain more insight into the role of B-MYB cells in we have employed affinity purification in conjunction with mass spectrometry to identify novel B-MYB interacting proteins. As a result we have characterized two members of the family of MYM-type zinc finger proteins, ZMYM4 and ZMYM2, as novel B-MYB binding proteins. ZMYM4 has been very poorly studied so far; apart from a single report showing that the *ZMYM4* gene is overexpressed in a fraction of human lung adenocarcinomas and squamous cell carcinomas, suggesting that it might have a role in cancer development^33^, virtually nothing is known about the function of ZMYM4. Our work, therefore, is the first characterization of the ZMYM4 protein.

Although we have shown that ZMYM4 binds to B-MYB at endogenous expression level, we have no evidence that ZMYM4 plays a major role in B-MYB dependent control of the cell cycle. ZMYM4 appeared neither to be recruited by B-MYB to the MYB-MuvB complex, nor did silencing of ZMYM4 have obvious effects on the cell cycle of HEK293 or HepG2 cells. The interaction of ZMYM4 and B-MYB was mediated by the central domains of both proteins and disrupted when serine residues 306, 307 and 309 of B-MYB were replaced by alanine. We previously observed that these residues are phosphorylated in response to double strand DNA breaks, resulting in a mobility shift of B-MYB^26^. We showed that induction of DNA-damage increased the binding of ZMYM4 to B-MYB and triggered phosphorylation of these residues, as evidenced by a B-MYB mobility shift (Fig. 3a), however, ZMYM4 did not interact preferentially with the phosphorylated form of B-MYB (Fig. 3b). The increased binding of ZMYM4 to B-MYB after DNA-damage, therefore, appeared not to be caused by the phosphorylation at serines 306, 307 and 309 but rather by other, as yet unknown modifications of B-MYB or ZMYM4, or another protein may be responsible for the increased interaction.

The central domain of B-MYB has been identified as a transcriptional activation domain that recruits the coactivator p300^34,35^. The binding site for p300 maps between amino acids 275 to 376 (numbering refers to murine B-MYB)^36^ and, hence, lies close to the binding site for ZMYM4. It is therefore conceivable that ZMYM4 interferes with the binding of p300 to B-MYB and thereby suppresses the p300-dependent stimulation of the B-MYB transactivation potential. Recently, we have shown that the activity of B-MYB was decreased after induction of DNA damage^26^, raising the possibility that ZMYM4 modulates the transactivation potential of B-MYB in response to DNA-damage. It will be interesting to investigate this in future work.

Our data have shown that ZMYM4 is SUMOylated, most likely at two positions. The fraction of SUMOylated ZMYM4 appeared to be quite high as almost 50% of ZMYM4 were present as slower migrating SUMOylated forms of the protein. We have observed that ZMYM4 interacts with ZMYM2, a multi-SUMO-binding protein^37^. Whether the SUMOylation of ZMYM4 is responsible for the interaction with ZMYM2 is an interesting possibility that remains to be explored. We have also observed that extent of SUMOylation of ZMYM4 was very low when cells were arrested in mitosis (Fig. 4e), suggesting that SUMOylation of ZMYM4 is regulated during the cell cycle. However, the exact function of the SUMO-modification of ZMYM4 remains to be studied in detail in future work.

Besides ZMYM2, the mass spectrometric analysis has identified several proteins as potential ZMYM4 interaction partners, including PRR12, UBQLN4 and UBQLIN1 as candidates with the highest scores in several independent experiments (Supplementary Table S2). Translocations involving *PRR12* have linked PRR12 to the development of intellectual disability, suggesting a role in neuronal development^38^. Ubiquilins4 and 1 are members of the Ubl-UBA family of ubiquitin-binding proteins that are involved in protein degradation pathways and link the ubiquitination machinery to the proteasome^39^. Recent work has also implicated UBQLN4 in the cellular response to DNA damage, showing that it affects the balance between homologous recombination and non-homologous end-joining in the repair of DNA double strand breaks^40^. It is intriguing that B-MYB has also been implicated in the response to DNA double strand breaks^26^ and that DNA damage strengthens the binding of ZMYM4 to B-MYB (Fig. 3). It will therefore be interesting to further explore a potential link between B-MYB and ZMYM4 in the DNA-damage response. Notably, another ZMYM family member, ZMYM3, is also involved in the response to DNA damage^41^. ZMYM4 also interacts with RCOR1 (also known as CoREST) (Supplementary Table S2) and KDM1A (also known as LSD1) (Supplementary Tables S6 to S8), both of which are are key components of LSD1-CoREST-HDAC transcriptional repressor complexes, suggesting a role of ZMYM4 in the regulation of transcription.

*ZMYM2* has initially attacted attention because it is involved in chromosomal translocations generating ZMYM2/FGFR1 fusion proteins that lead to constitutive FGFR1 signaling and the development of a myeloproliferative disease eventually progressing to AML^42,43^. ZMYM2 contains several SUMO-interaction motifs (SIMs), allowing it to interact with target proteins conjugated to several SUMO moieties^37,44,45^. Similar to ZMYM4, ZMYM2 is part of a chromatin-bound ZMYM2-LSD1-CoRest-HDAC1 transcriptional repressor complex^46^. More recently, ZMYM2 was shown to interact with NANOG and to inhibit NANOG-mediated cell reprogramming^47^. Our new data have shown that ZMYM2 binds to the same region of B-MYB as ZMYM4, however, its binding appears to be weaker than that of ZMYM4. Although ZMYM2 and ZMYM4 bind to each other, we have no evidence that they interact with B-MYB as a complex. Unlike ZMYM4, ZMYM2 plays a role at the G1/S-phase transition of the cell cycle, possibly in conjunction with B-MYB. Knockdown of ZMYM2 or of B-MYB strongly impaired the G1/S-transition in HepG2 cells (Fig. 7), suggesting that they are both required to promote the G1/S-transition. Interestingly, in HEK293 cells neither B-MYB nor ZMYM2 appeared to be required for entry into S-phase. This may reflect the fact that HEK293 cells, in contrast to HepG2 cells, lack a functional G1/S-checkpoint due to the expression of the adenoviral E1A protein, and raises the possibility that the function of both proteins is linked to this cell cycle checkpoint. The exact roles of B-MYB and ZMYM2 at the G1/S-transition are currently not known. Likewise, it is not entirely clear whether they act independently or cooperate with each other to facilitate S-phase entry. Our observation that simultaneous knockdown of B-MYB and ZMYM2 reduced the requirement of ZMYM2 for S-phase entry (Fig. 7c) provided a first hint that the role of ZMYM2 at the G1/S-transition may be dependent on B-MYB. The mechanistic basis of this dependency and the possible role of the interaction of both proteins warrants further investigation in the future.

In summary, we have identified two members of the ZMYM-family of zinc finger proteins as novel B-MYB binding partners. Our characterization of ZMYM4 and ZMYM2 suggest that they may have roles in the DNA-damage response and the G1/S cell cycle transition.

## Methods

### Cells

Human HEK293, HEK293T and HepG2 cells were grown in DMEM and RPMI1640 medium containing 10% fetal calf serum. Quail QT6 fibroblasts were grown in Iscove’s medium supplemented with 8% fetal calf serum and 2% chicken serum. Cells were arrested at the G1/S-boundary by adding 4 mM thymidine (Thermo Fisher Scientific) or 0.5 mM mimosine (Sigma-Aldrich) for 20 h to the medium^29^. To arrest cells in mitosis, 0.5 μM STLC (S-Trityl-L-cystein, St. Cruz Biotechnology) was added for 20 h.

### Cell cycle analysis

Cell cycle analysis was performed as described before^20^. Briefly, cells were trypsinized, fixed with 70% ice-cold ethanol in PBS for 1 h or longer at −20 °C, washed with PBS (+0.5% BSA) and stained with propidium iodide (50 μg/mL PI and 25 μg/mL RNase A in PBS) for 1 h at room temperature. Flow cytometry was performed with a Beckman-Coulter Cytomics FC500 flow cytometer. 15.000 cells were counted per condition in every experiment.

### Antibodies

B-MYB was detected with the mouse monoclonal antibody LX015.1^48^. Immunoprecipitation of B-MYB was performed with rabbit antisera raised against the N-terminal (anti B-MYB#53) or the C-terminal (anti B-MYB Brian) part of B-MYB^15,49^. Antiserum against Lin9 was kindly provided by S.Gaubatz^50^. In addition, the following commercially available antibodies were used: mouse anti-GFP (Sigma-Aldrich, 7.1 + 13.1), mouse anti-β-actin (Sigma-Aldrich, AC-15), mouse anti Flag (Sigma-Aldrich, M2), Mouse anti HA-tag (Hiss Diagnostics, HA.11), rabbit anti-ZMYM4 (Biomol, A301-809A) and rabbit anti-ZMYM2 (Abcam, ab106624).

### Expression vectors and transfections

All human B-MYB expression vectors have been described before^26^. Briefly, peGFP-B-MYB encodes a fusion protein of eGFP and full-length human B-MYB. Truncated derivatives of human B-Myb encoding amino acids 1–207, 206–700, 206–509, 1–207 plus 485–700, 485–700, 1–509, 335–700, 207–335, 335–509 and 272–700 fused to eGFP were generated by cloning the corresponding parts of the B-MYB coding region into peGFP-C1, 2 or 3, utilizing appropriate restriction sites. Expression vectors for GFP-B-MYB BM1, 2, 3 and 4 were generated by PCR and contain human B-MYB amino acids 190–260 (BM1), 260–355 (BM2), 355–435 (BM3) and 435–502 (BM4), fused C-terminally to GFP. B-MYB point mutants Mut6 (S282A,T286A,S287A,S306A,S307A,S309A), Mut3A (S282A,T286A,S287A) and Mut3B (S306A,S307A,S309A) were constructed by PCR using appropriate oligonucleotides to introduce the mutations.

ZMYM4 expression plasmids are derived from pCR-BluntII-TOPO-ZMYM4 (Source BioScience, Nottingham, UK), which contains the coding region of human ZMYM4 from amino acid 33 to 1548. pCDNA3-HA-ZMYM4 was generated by PCR amplification of the 5′ part of the ZMYM4 coding sequence using the oligonucleotides 5′-ACTGGTACCATGGCTTACCCATACGATGTTCCAGATTACGCTGCCTCGAGCATGGATACAGAAATGTCTGAAGATATAGACCACAA-3′ and 5′-CATTCTGGTAATTAACTTCATGTCGAATAATAGCATTCTTCTGACACATG-3′ as forward and reverse primers. The resulting DNA fragment was digested with KpnI and NdeI and cloning together with a NdeI/BamHI fragment encompassing the 3′ part o the ZMYM4 coding region between the KpnI and BamHI sites of pCDNA3. pLVX-GFP-ZMYM4 was generated by isolating the GFP-coding region (together with the 3′ part of the CMV promoter) as an NdeI/XhoI fragment from the pEGFP-C2 plasmid and the ZMYM4 coding regions as an XhoI/BamHI Fragment from pCDNA3-HA-ZMYM4. Both fragment were then cloned together between the NdeI and BamHI sites of pLVX-dsRed. Deletion constructs pCDNA3-HA-ZMYM4 (33–425), pCDNA3-HA-ZMYM4 (33–1077), pCDNA3-HA-ZMYM4 (425–1077) and pCDNA3-HA-ZMYM4 (1077–1548) of pCDNA3-HA-ZMYM4 were obtained by PCR with appropriate primers and cloning into pCDNA3. The expression vector for Flag-tagged human ZMYM2 was obtained from A.Sharrocks^37^. Expression vectors for wild-type and mutant SENP1 were obtained from O.S.Gabrielsen^51^.

### RNA interference

B-MYB expression was silenced with siRNA duplexes targeting the sequence 5′-GAAACAUGCUGCGUUUGUA-3′ (B-MYB siRNA4)^20^. ZMYM2 expression was silenced with duplexes targeting the sequence 5′-GGCUGCAAAUUAUUAUACA-3′ (ZMYM2 siRNA2) or 5′-CCACUGCUUUAAUAGAUAU-3′ (ZMYM2 siRNA3). ZMYM4 expression was silenced with duplexes targeting the sequence 5′-GCUACCCAUUUGCCAAUAA-3′ (ZMYM4 siRNA1) or 5′-GGUUGCAAAUUGCUUUAUA-3′ (ZMYM4 siRNA2). siRNA targeting Renilla luciferase (5′-AAACAUGCAGAAAAUGCUG-3′) was used as negative control. Cells were grown in 6 cm petri dishes to approximately 50–80% confluence and received 2.5 ml fresh growth medium before transfection. 100 nM siRNA was then transfected using Lipofectamine RNAiMAX Transfection Reagent (Invitrogen), according to manufacturers’ protocols. Cells were harvested 48–72 h later and analyzed by flow cytometry and Western blotting.

### ^3^H-thymidine labeling

Cells were incubated with growth medium supplemented with 10 μCi/ml 3H-thymidine for 1 h^52^. The cells were then washed with PBS, lysed in PBS containing 1% SDS and heated to 95 °C to reduce the viscosity. Aliquots were then spotted on Whatman filter paper and washed 2 times for 15 min with 10% trichloracetic acid (TCA) and once with ethanol. The filter paper was dried and the radioactivity was determined in a scintillation counter. Aliquots of the lysed cells were analyzed for expression of β-actin by SDS-PAGE and Western blotting to correct for differences in cell numbers between ZMYM2-specific and control knockdown samples.

### Lentiviral infections

Lentiviral expression vectors were constructed by replacing the RFP-coding sequence of plasmid pLVX-DsRed-Monomer-C1 (Clontech) with the sequences encoding GFP, GFP-B-MYB or GFP-ZMYM4. The resulting DNAs were cotransfected with the lentiviral packaging plasmids *pMD2.G and pSPAX2* into HEK293T cells to generate infectious viral particles, followed by infection of HEK293T cells and puromycin selection to eliminate uninfected cells^20^.

### Co-immunoprecipitation and GFP-trap analysis

Co-immunoprecipitation and GFP-trap experiments were performed as described before^26^, Briefly, cells were lysed in ELB buffer (50 mM Tris/HCl pH 7,5; 120 mM NaCl; 20 mM NaF; 1 mM EDTA; 6 mM EGTA; 15 mM sodium pyrophosphate; 1 mM phenylmethylsulfonyl fluoride; 0,2% NP-40 and a protease inhibitor mix containing Aprotinin, Leupeptin and Pepstatin). After incubation on ice for 15 min, lysates were centrifuged at 14 000 × g for 15 min, and the supernatant was used as total cell extract. Immunoprecipitations were performed overnight at 4 °C. Immune complexes were bound to Protein-A Sepharose beads, collected by centrifugation, washed with lysis buffer and finally subjected to SDS-PAGE. Proteins were transferred to nitrocellulose membranes and stained with the appropriate antibodies. For GFP-trap experiments, cells expressing GFP or GFP fusion proteins were lysed in ELB buffer as described above and the supernatant was used as total cell extract. Aliquots of cell extracts were then incubated with GFP-trap beads (Chromotec, München) for 3 h at 4 °C. Beads were washed 4 times with ELB buffer. Bound proteins and input samples were analyzed by Western blotting with appropriate antibodies. In some experiments cells were lysed directly with SDS-sample buffer or with ELB buffer containing 20 mM N-ethyl maleimide (NEM).

### Mass spectrometry

Mass spectrometry was performed as described^53^. Briefly, cells from eight subconfluent 10 cm plates of HEK293T cells stably expressing GFP-B-MYB were lysed in ELB-buffer (50 mM Tris/HCl pH 7,5; 120 mM NaCl; 1 mM EDTA; 6 mM EGTA; 0,2% NP-40). GFP-B-MYB was recovered from the cell extract by binding to magnetic GFP-trap beads (Chromotek). The beads were reduced using 20 µl DTT (1 mM in 100 mM NH_4_HCO_3_) for 30 min hat 56 °C. Subsequently beads were alkylated using 10 µl 2-iodoactamide (10 mM in 100 mM NH_4_HCO_3_) for 30 min at room temperature and washed twice with ddH_2_O. The beads were incubated with 40 µl digestion solution (200 ng Trypsin, 0.01% ProteaseMax, 50 mM NH_4_HCO_3_) at 37 °C for 2 h. LS/MS² analysis of 20 µL peptide solution was performed using an UltiMate 3000 RS LC nano system (Thermo Fisher Scientific GmbH, Dreieich, Germany) connected to a maXis II UHR-qTOF mass spectrometer with a nano-ESI source (CaptiveSpray with nanoBooster, Bruker Daltonik GmbH, Bremen, Germany). The sample was acidified with formic acid to a final concentration of 0.1%, loaded on a C18 trapping column (Acclaim PepMap100, 5 μm, 100 Å, ID 300 μm × L 5 mm, Thermo Fisher Scientific GmbH, Dreieich, Germany) at a flow rate of 20 μL/min in 2% eluent B (eluent A: 0.1% formic acid in water; eluent B: 0.1% formic acid in acetonitrile). After 5 minutes of washing at 2% B, a 120 minute gradient (2% to 50% B, flow rate 300 nL/min) was applied for fractionation on a C18 nano column (Acclaim PepMap 100 C18, 2 μm, 100 Å, ID 0.075 mm × L 250 mm, Thermo Fisher Scientific GmbH, Dreieich, Germany). MS settings: capillary voltage 1.200 V, mass range: m/z 150–2200. MS survey scans were performed with a cycle time of 3 s. After each survey scan, the 10 to 20 most abundant precursor ions with z > 1 were selected for fragmentation using collision-induced dissociation. MS/MS summation time was adjusted depending on the precursor intensity, the precursor isolation window and the collision energy were depending on the precursor m/z and charge. DataAnalysis 4.4 (Bruker Daltonik GmbH, Bremen, Germany) was used for chromatogram processing and fragment spectra isolation. The resulting data were analyzed using ProteinScape 4 (Bruker Daltonik GmbH, Germany) as a front-end for searches against the SWISS-PROT databases on a Mascot server (Mascot 2.5, Matrix Science Ltd., London, UK).

## Supplementary information

Supplementary information.
