# Supplementary material for: Characterization of the zinc finger proteins ZMYM2 and ZMYM4 as novel B-MYB binding proteins
Source: Sci Rep. 2020 May 21;10:8390. doi: 10.1038/s41598-020-65443-w (PMC7242444; doi:10.1038/s41598-020-65443-w)
Supplement: Supplementary file 1 — Supplementary information. [file 41598_2020_65443_MOESM1_ESM.pdf]

Supplementary information

Characterization of the zinc finger proteins ZMYM2 and ZMYM4 as novel B-MYB binding proteins

Hannah Cibis, Abhiruchi Biyanee, Wolfgang Dörner, Henning D. Mootz and Karl-Heinz Klempnauer

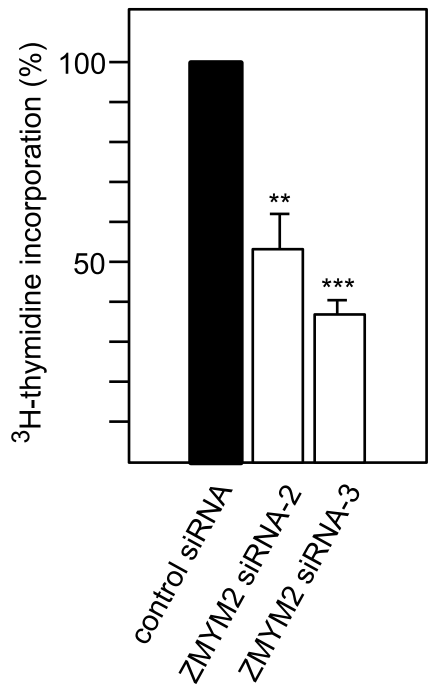

Supplementary Fig. S1 **Effect of silencing of ZMYM2 on the DNA-synthesis in HepG2 cells.** HepG2 cells treated for 72 hours with control or ZMYM2-specific siRNAs were incubated for 1 h in medium supplemented with 10 mCi/mL ^{3}H-thymidine. Radioactivity incorporated into the cells was determined by TCA-precipitation followed by liquid scintillation counting. The bars indicate the percent incorporation (with standard deviation) relative to control siRNA-treated cells. Asterisks indicate statistical significance (**p < 0.01; ***p < 0.001, Student's t-test).

protein	experiment 1		experiment 2		experiment 3		crapome	remarks
	GFP-B-MYB	GFP	GFP-B-MYB	GFP	GFP-B-MYB	GFP		
MYBB_HUMAN	3054,9	0	2854,8	0	2600,1	0		
P5CS_HUMAN	1231,7	0	1527,9	0	1337,4	0	93/411	mitochondria
ZMYM4_HUMAN	992,8	0	783,8	0	747,2	0	27/411	nucleus
LIN9_HUMAN	523,7	0	393,3	0	734,9	0	1/411	LIN complex
ATD3B_HUMAN	446,5	0	427,9	0	320,8	0	59/411	mitochondria
PGES2_HUMAN	368	0	164,8	0	440,5	0	3/411	mitochondria
ATD3A_HUMAN	354,8	0	234,8	0	552,3	0	87/411	mitochondria
ACD11_HUMAN	307,7	0	202,6	0	562,8	0	6/411	cytosol
ECHA_HUMAN	278,6	0	283,4	0	654,8	0	72/411	mitochondria
ACOX1_HUMAN	216,7	0	378,6	0	588,2	0	4/411	peroxisomes, nucleolus
PRKDC_HUMAN	194,2	0	28,4	0	33,6	0	175/411	nucleus
RBBP4_HUMAN	162,8	0	33,3	0	315,2	0	148/411	LIN complex
ECHB_HUMAN	128,7	0	96	0	281	0	47/411	mitochondria
LRC59_HUMAN	128	0	103,5	0	185,5	0	80/411	cytoplasmic membranes, endoplasmatic reticulum, nuclear envelope
RS5_HUMAN	113,7	0	141,9	0	65,8	0	160/411	ribosomal protein
APLP2_HUMAN	102,6	0	197,7	0	149,5	0	1/411	cytoplasmic vesicles
LIN54_HUMAN	102,5	0	118,2	0	368,5	0	6/411	LIN complex
ARL1_HUMAN	73,6	0	40,1	0	55,7	0	28/411	Golgi apparatus
LIN37_HUMAN	60,5	0	20,1	0	151,5	0	1/411	LIN complex
TCPZ_HUMAN	51,2	0	23,5	0	24,6	0	177/411	cytosol
PDXL2_HUMAN	47,2	0	20,4	0	137,4	0	0/411	Golgi apparatus
LIN52_HUMAN	43,8	0	83,4	0	170,4	0	0/411	LIN complex
DHB12_HUMAN	31,6	0	35,5	0	36,3	0	27/411	cytoplasmic membranes, endoplasmatic reticulum

Supplementary table S1. **Identification of B-MYB interacting proteins**. List of proteins detected in three independent MS/MS-experiments as B-MYB interacting proteins. The numbers refer to Mascot scores.

protein	experiment 1		experiment 2		experiment 3			
	GFP-ZMYM4	GFP	GFP-ZMYM4	GFP	GFP-ZMYM4	GFP	crapome	remarks
ZMYM4_HUMAN	9656,3	0	9700,8	0	6526,7	0		
UBQL4_HUMAN	1266,8	0	220,9	0	1235,0	0	4/411	nucleoplasm
PRR12_HUMAN	1003,1	0	1000,8	0	740,9	0	3/411	nucleus, cytosol
ZMYM2_HUMAN	710,6	0	936,4	0	544,2	0	17/411	nucleoplasm
UBQL1_HUMAN	667,9	0	44,0	0	774,2	0	10/411	cytosol, nucleoplasm
YYAP1_HUMAN	499,2	0	605,9	0	267,8	0	5/411	nucleolus
SALL2_HUMAN	393,0	0	264,9	0	155,8	0	3/411	nucleoplasm, vesicles
PUR6_HUMAN	370,5	0	39,8	0	85,2	0	106/411	cytosol
ZMYM3_HUMAN	348,9	0	400,1	0	210,1	0	23/411	nucleoplasm
ZSWM3_HUMAN	310,1	0	453,0	0	103,0	0	0/411	vesicles, cytosol, nucleoli
DYL2_HUMAN	263,0	0	103,5	0	153,1	0	38/411	cytosol, nucleoplasm
ILVBL_HUMAN	257,2	0	291,5	0	88,2	0	9/411	Golgi, vesicles
ZSWM1_HUMAN	147,4	0	189,5	0	55,3	0	0/411	cytosol, plasma membrane
DECR_HUMAN	142,6	0	877,8	0	35,1	0	15/411	mitochondria, cytosol
RCOR1_HUMAN	115,6	0	125,4	0	122,7	0	22/411	nucleoplasm, REST corepressor
SYLC_HUMAN	89,0	0	96,2	0	13,8	0	131/411	cytosol nuclear bodies
AP1G1_HUMAN	78,4	0	470,2	0	55,2	0	4/411	vesicles, cytosol
HS105_HUMAN	69,6	0	40,6	0	67,4	0	119/411	cytosol, nucleoplasm
SIAH1_HUMAN	70,4	0	52,8	0	41,1	0	5/411	mitochondria, nucleoplasm
PSA5_HUMAN	67,1	0	59,4	0	18,2	0	88/411	cytosol
CS068_HUMAN	66,6	0	252,8	0	80,2	0	n/a	nucleoplasm
RHG35_HUMAN	29,5	0	47,1	0	21,8	0	12/411	nuclear bodies
MYCB2_HUMAN	25,3	0	32,5	0	45,4	0	6/411	nucleoplasm, vesicles
HDHD5_HUMAN	23,1	0	19,3	0	22,1	0	12/411	mitochondria
POGZ_HUMAN	23,0	0	114,4	0	27,5	0	25/411	nucleoplasm

Supplementary table S2. **Identification of ZMYM4 interacting proteins**. Proteins detected in three independent MS/MS-experiments as potential ZMYM4 interaction partners are listed in the table. The numbers refer to Mascot scores.

Supplementary table S3: Raw mass spectrometry data for GFP-B-MYB experiment 1

Protein	Scores A GFP-B-Myb	Scores B GFP	Peptides A	Peptides B	SC (A) [%]	SC (B) [%]
MYBB_HUMAN	3054,9		42		61,7	
TBB4B_HUMAN	1730,9	1068,7	26	22	66,5	64,3
TBB4A_HUMAN	1698,8	1031,9	24	22	66,4	64,0
TBB5_HUMAN	1639,6	977,8	25	21	66,7	64,4
TBA1B_HUMAN	1372,7	1073,2	23	20	75,8	61,9
TBA1A_HUMAN	1322,2		22		72,5	
TBB2B_HUMAN	1322,2		21		53,3	
P5CS_HUMAN	1231,7		21		44,5	
TBA1C_HUMAN	1219,5	827,5	20	16	69,5	52,6
ZMYM4_HUMAN	992,8		24		29,5	
HS71A_HUMAN	921,3	397,9	19	10	44,6	20,1
HSP7C_HUMAN	835	385,2	17	10	41,8	21,1
TBB6_HUMAN	812,9	417,1	15	9	51,1	26,9
CH60_HUMAN	579,4	258,8	12	6	42,9	17,1
LIN9_HUMAN	523,7		10		35,1	
GRP78_HUMAN	487,7	131,6	13	3	27,1	7,5
ATD3B_HUMAN	446,5		9		24,4	
GRP75_HUMAN	401,6	200,7	11	4	23,6	9,4
PGES2_HUMAN	368		7		33,7	
ATD3A_HUMAN	354,8		10		23,7	
ACD11_HUMAN	307,7		8		22,1	
ACTB_HUMAN	297,6	101,7	5	2	28,8	9,1
ECHA_HUMAN	278,6		5		10,0	
PYR1_HUMAN	265,9	24,5	8	1	9,5	1,2
EF1A1_HUMAN	257,6	138,8	7	5	30,7	30,5
EFTU_HUMAN	255	212,9	4	4	15,3	15,3
K2C1_HUMAN	254,2	186,9	6	4	16,5	7,8
THIL_HUMAN	247,2	125,4	5	3	23,7	15,5
C1QBP_HUMAN	231,2	449,4	5	8	37,2	53,5
ACOX1_HUMAN	216,7		5		13,3	
PRKDC_HUMAN	194,2		5		2,8	
RBBP4_HUMAN	162,8		3		11,3	
ATPA_HUMAN	161,2	157,4	4	3	19,2	9,2
SCPDL_HUMAN	160,3		5		30,1	
K1C10_HUMAN	158	49,6	4	1	16,4	3,3
RS3_HUMAN	146,1	176,8	5	5	24,7	26,3
AT1A1_HUMAN	130,6		3		6,5	
ECHB_HUMAN	128,7		3		12,9	
LRC59_HUMAN	128		4		22,1	
AT2A2_HUMAN	116,9		3		6,1	
RU2A_HUMAN	114,3		3		22,7	
TCPA_HUMAN	113,9		3		16,2	
RS5_HUMAN	113,7		3		23,5	
RCN2_HUMAN	108,4	16,9	3	1	19,6	7,3
RS27A_HUMAN	104	88,8	2	2	22,4	23,1
APLP2_HUMAN	102,6		2		5,0	
LIN54_HUMAN	102,5		3		7,7	
RS16_HUMAN	96,9	62,1	3	1	16,4	7,5
H2B1K_HUMAN	90,3		1		11,9	
EMD_HUMAN	87,2		1		5,9	
SSRD_HUMAN	85,8	65,9	2	2	17,3	17,3
RAB1C_HUMAN	83,8	21,2	2	1	13,4	8,0
K1C9_HUMAN	75,4	72,3	2	2	13,2	7,7
RL23_HUMAN	74,2	30,9	2	1	25,0	14,3
ARL1_HUMAN	73,6		1		8,8	
TCPE_HUMAN	73,4		2		10,9	
DYL1_HUMAN	72,2		1		24,7	
SERA_HUMAN	71,3	26,2	3	1	7,5	2,3
RS2_HUMAN	71,2	14,8	1	1	4,4	3,8
MD2L1_HUMAN	70,9	49,1	1	1	10,2	7,8
K22E_HUMAN	68,9		3		13,5	
RPN1_HUMAN	68,2	31,9	1	1	4,1	1,8
FUBP2_HUMAN	60,9	26,2	2	1	4,1	2,5
HDAC6_HUMAN	60,8	60	2	2	2,9	4,4

LIN37_HUMAN	60,5		1		7,3	
CALX_HUMAN	58,5	44,6	1	2	2,7	6,3
XPO2_HUMAN	58,1		2		6,7	
AR6P1_HUMAN	57,9		1		13,8	
RS15_HUMAN	57		2		28,3	
DCAKD_HUMAN	52,1		1		5,6	
RS27_HUMAN	51,7	55,9	1	1	15,5	15,5
TRAP1_HUMAN	51,7		1		2,0	
PP1G_HUMAN	51,4	16,2	1	1	5,3	5,3
SPTC1_HUMAN	51,3		1		3,0	
S61A1_HUMAN	51,2		2		7,6	
TCPZ_HUMAN	51,2		2		9,4	
DNJA1_HUMAN	49,7	41,4	1	1	6,0	3,3
RB11A_HUMAN	47,5		1		13,4	
SYMC_HUMAN	47,3		1		3,1	
PDXL2_HUMAN	47,2		1		4,1	
PYRG1_HUMAN	45,7		2		9,0	
ADT2_HUMAN	44,6	22,5	2	1	7,4	3,0
RS30_HUMAN	44,5	51,7	1	1	16,9	16,9
XPO1_HUMAN	43,9		2		3,5	
LIN52_HUMAN	43,8		2		31,0	
OST48_HUMAN	42,9		1		6,4	
RS18_HUMAN	42,5	26,9	1	1	5,3	5,3
MIMIT_HUMAN	41,1		1		10,7	
DNJA3_HUMAN	40,9		1		4,8	
LPPRC_HUMAN	40,4		1		2,1	
RL11_HUMAN	39,9	62,9	1	1	7,9	7,9
DJC10_HUMAN	37,1	66,3	1	2	2,1	3,7
ATPB_HUMAN	36,6		1		4,5	
TIM44_HUMAN	36,5	44,9	1	1	3,1	3,1
TCPB_HUMAN	35,7	18,7	1	1	4,1	4,1
RS26_HUMAN	35,5		1		13,0	
MCM7_HUMAN	35,5		1		3,6	
PGRC1_HUMAN	34,4		1		7,7	
MSRB2_HUMAN	33,6	162,3	1	5	15,9	40,7
NUCL_HUMAN	31,9		1		3,1	
DHB12_HUMAN	31,6		1		4,8	
DNJA2_HUMAN	31		1		7,3	
IRS4_HUMAN	27,5		1		2,2	
TBR1_HUMAN	26,9		1		1,8	
HNRPU_HUMAN	24,3		1		2,7	
RBM39_HUMAN	24,1		1		2,1	
SSRA_HUMAN	23,3	38,1	1	1	5,2	5,2
PEF1_HUMAN	22	71,1	1	2	10,9	13,4
TM109_HUMAN	22		1		4,9	
ILVBL_HUMAN	21,7		1		4,9	
SURF4_HUMAN	21,7		1		6,7	
HV311_HUMAN	19,7		1		19,7	
TCPD_HUMAN	19,1		1		5,4	
BASI_HUMAN	17,9	21,5	1	1	4,7	4,7
VIME_HUMAN	17,6		1		3,0	
RL10_HUMAN	17,1	13,9	1	1	12,6	12,6
AL1B1_HUMAN	16,1		1		3,3	
TCPH_HUMAN	15,9		1		4,4	
CCD47_HUMAN	15,7		1		5,0	
RS10_HUMAN	15,5		1		5,5	
ASB11_HUMAN	15,2		1		4,6	
EF1G_HUMAN	14,6		1		5,0	
GCDH_HUMAN	14,5		1		5,9	
TRIM4_HUMAN	14,3		1		2,2	
TAM41_HUMAN	14,2		1		5,3	
S10AG_HUMAN	13,7		1		18,4	
SFTPD_HUMAN	13,4		1		6,4	

Supplementary table S4: Raw mass spectrometry data for GFP-B-MYB experiment 2

Protein	Scores A GFP-B-Myb	Scores B GFP	Peptides A	Peptides B	SC (A) [%]	SC (B) [%]
MYBB_HUMAN	2854,8		38		63,1	
P5CS_HUMAN	1527,9		22		48,9	
TBB4B_HUMAN	1282	987	20	16	60,2	48,8
TBB4A_HUMAN	1276,2		20		59,9	
TBB5_HUMAN	1173,4	995,6	20	17	60,4	52,9
TBA1B_HUMAN	1162,7	1132,7	16	20	58,5	57,4
TBA1A_HUMAN	1125,3		15		55,4	
TBA1C_HUMAN	1069,5	902,9	16	15	58,8	48,1
TBB2B_HUMAN	1058,7	866	17	14	51	46,7
ZMYM4_HUMAN	783,8		17		19,4	
HSP7C_HUMAN	615,1	394,9	13	9	26,6	17,8
TBB6_HUMAN	596,5	477,5	11	8	35,7	25,6
CH60_HUMAN	564,3	295,2	10	4	36,6	13,8
GRP78_HUMAN	497,8	271,2	9	5	18,8	12,5
ATD3B_HUMAN	427,9		10		21,6	
HS71A_HUMAN	423,2	314,7	8	6	24,5	12,3
LIN9_HUMAN	393,3		7		24,2	
ACOX1_HUMAN	378,6		8		21,8	
ECHA_HUMAN	283,4		6		14	
RS3_HUMAN	261,8	280,4	7	6	39,5	30,5
GRP75_HUMAN	240,2	254,7	6	5	13,1	12,5
ATD3A_HUMAN	234,8		7		13,2	
PYR1_HUMAN	228,8	96,8	3	2	3,1	2,2
ACTB_HUMAN	204,8	240,2	4	4	20,8	20,8
ACD11_HUMAN	202,6		7		19,1	
EFTU_HUMAN	201,6	211	3	3	11,1	11,1
APLP2_HUMAN	197,7		3		7,1	
AT1A1_HUMAN	183,4	314,5	5	5	9,6	8,9
PGES2_HUMAN	164,8		2		12,5	
SERA_HUMAN	164,7	114	3	4	9,6	10,7
ATPA_HUMAN	164,4	193,6	3	5	9,2	11,9
C1QBP_HUMAN	161,8	495,3	4	9	30,1	53,5
EMD_HUMAN	157,1	86	2	1	14,2	5,9
RS5_HUMAN	141,9		3		23,5	
RCN2_HUMAN	133,1	52,6	3	1	18,3	7,3
TCPA_HUMAN	131,9		2		8,8	
HS90A_HUMAN	131	149,4	2	3	4	6,7
HS90B_HUMAN	121,8	197,7	2	4	4	9,4
RS16_HUMAN	120,4	83,6	3	2	16,4	16,4
LIN54_HUMAN	118,2		3		6,7	
NUCL_HUMAN	109,1	71	4	2	10,8	5,1
LRC59_HUMAN	103,5		4		22,1	
ECHB_HUMAN	96		4		17,5	
DYL1_HUMAN	83,7	64,1	1	1	24,7	24,7
LIN52_HUMAN	83,4		2		31	
RS27A_HUMAN	79,8	234,6	2	4	22,4	23,1
THIL_HUMAN	78,6	129,3	1	2	4,2	11
RS27_HUMAN	78,3	91,2	1	1	15,5	15,5
K1C10_HUMAN	77,2		1		3,3	
CALX_HUMAN	70,6	77,7	1	1	2,7	2,7
XPO2_HUMAN	69	21,5	1	1	1,5	1,5
EF1A1_HUMAN	67,4	101,7	1	1	6,1	6,1
YBOX1_HUMAN	66,7	89	1	2	5,2	11,1
SYMC_HUMAN	66,6		1		3,1	
K2C1_HUMAN	66,3	76	2	3	7,9	9,8
RAB5C_HUMAN	65,4		1		11,1	
SSRG_HUMAN	60,5	62,2	1	1	7,6	7,6
RAB1B_HUMAN	56,9		1		9,5	
FUBP2_HUMAN	56,7	124,4	2	3	6,6	8,2
RL23_HUMAN	54,7	28,7	1	1	14,3	10,7
MIF_HUMAN	50,9		1		18,3	
RL11_HUMAN	49,6	28,4	1	1	7,9	7,9
KPYM_HUMAN	47,9	43,4	1	1	3,4	3,4
EF1G_HUMAN	46,7		1		6,4	

P53_HUMAN	45,6		1		9,2	
RPN1_HUMAN	44,8	174,2	2	4	4,4	8,4
MCM7_HUMAN	44,6		1		3,6	
SSRD_HUMAN	41,8	47,7	1	2	6,4	17,3
SURF4_HUMAN	41,2	34,2	1	1	6,7	6,7
RS18_HUMAN	41	45	1	1	5,3	5,3
KCRB_HUMAN	40,1		2		7,3	
ARL1_HUMAN	40,1		1		8,8	
IF5A1_HUMAN	38,7		2		30,5	
HNRPF_HUMAN	38	125,1	1	3	4,1	12
DHB12_HUMAN	35,5		2		8,7	
AT2A2_HUMAN	34,2		1		2,5	
RBBP4_HUMAN	33,3		1		3,1	
RS23_HUMAN	33,1	155,4	1	3	18,9	27,3
TCPB_HUMAN	31,6		1		4,1	
AR6P1_HUMAN	31,1		1		13,8	
THOC4_HUMAN	30,8	36,9	1	1	7	7
RPN2_HUMAN	30,6	103,6	1	2	1,9	5,7
IQGA3_HUMAN	30,4		1		1,6	
BASI_HUMAN	29,8	49,9	1	2	4,7	8,3
SYFA_HUMAN	29,6		1		2,8	
PCBP1_HUMAN	29,4	101,6	1	1	5,6	5,6
PRKDC_HUMAN	28,4		1		0,5	
RAB2A_HUMAN	27,6	50,6	1	2	6,1	12,7
MD2L1_HUMAN	27,1	19,8	1	1	10,2	4,9
PGRC1_HUMAN	27	22,6	1	1	7,2	7,2
RU2A_HUMAN	24,6		1		5,5	
TCPZ_HUMAN	23,5		1		3,2	
TCPD_HUMAN	22,7		1		2,4	
COF1_HUMAN	21,8		1		16,9	
PDXL2_HUMAN	20,4		1		2,5	
EF2_HUMAN	20,2	42	1	1	1,5	2,1
LIN37_HUMAN	20,1		1		10,6	
TBR1_HUMAN	19,4	16,1	1	1	1,8	1,8
AL1B1_HUMAN	18,9		1		2,7	
TRY2_HUMAN	18,1		1		8,1	
PP1G_HUMAN	17,7		1		5,3	
HV353_HUMAN	17,4		1		19,8	
OST48_HUMAN	17,2	46,5	1	2	6,4	5,7
GCDH_HUMAN	16,6		1		5,9	
AAAT_HUMAN	15,8	23,4	1	1	3,7	3,7
IF4A1_HUMAN	15,8		1		5,2	
G3P_HUMAN	15,5	17,8	1	1	4,5	4,5
TIM50_HUMAN	15,4	73,2	1	2	3,1	9,3
SFI1_HUMAN	14,5		1		1,4	
LMO7_HUMAN	14,3		1		1,1	
CBPC2_HUMAN	14,2		1		1,7	
K2C79_HUMAN	13,7		1		2,2	
AKAP1_HUMAN	13,6		1		2,2	

Supplementary table S5: Raw mass spectrometry data for GFP-B-MYB experiment 3

Protein	Scores A GFP-B-Myb	Scores B GFP	Peptides A	Peptides B	SC (A) [%]	SC (B) [%]
MYBB_HUMAN	2600,1		37		61,7	
TBB5_HUMAN	1350,3	1157,2	23	21	57,9	62,2
P5CS_HUMAN	1337,4		23		52,2	
TBA1B_HUMAN	1237,3	845,3	18	15	58,5	52,5
TBB4B_HUMAN	1231,6	1064,5	20	19	53,7	51,9
TBA1C_HUMAN	1100,5	860,4	17	16	55,9	58,1
TBB2A_HUMAN	896,7	807,3	16	14	38,2	42,7
HSP7C_HUMAN	878,1	491,9	18	11	46,3	22,9
HS71A_HUMAN	866,2	521,8	18	14	42,6	37,1
ZMYM4_HUMAN	747,2		15		21,1	
LIN9_HUMAN	734,9		12		43,7	
ECHA_HUMAN	654,8		12		30,9	
GRP78_HUMAN	609,6	141,6	13	3	33,6	5,4
ACOX1_HUMAN	588,2		13		30,8	
ACD11_HUMAN	562,8		15		42,3	
ATD3A_HUMAN	552,3		10		24,3	
PGES2_HUMAN	440,5		10		42,4	
CH60_HUMAN	409,1	109,7	9	3	31,6	11
LIN54_HUMAN	368,5		7		17	
ATD3B_HUMAN	320,8		5		10,6	
RBBP4_HUMAN	315,2		6		29,2	
GRP75_HUMAN	308,6	99,5	7	3	16,8	5,4
RU2A_HUMAN	289,1	34,1	4	2	29,4	19,2
ECHB_HUMAN	281		6		27,4	
PYR1_HUMAN	220,9		5		4,5	
XPO2_HUMAN	205,8		4		7,1	
LRC59_HUMAN	185,5		5		31,3	
HNRPU_HUMAN	184,6	177,3	5	3	11,3	6,7
LIN52_HUMAN	170,4		4		54,3	
RAB1A_HUMAN	160,5		4		32,7	
RAB1B_HUMAN	158,7		4		31,3	
LIN37_HUMAN	151,5		3		23,2	
APLP2_HUMAN	149,5		2		4,5	
DCAKD_HUMAN	147,3		4		25,1	
NUCL_HUMAN	141,1	33,1	3	1	8	3,1
PDXL2_HUMAN	137,4		2		6,4	
SYEP_HUMAN	118		2		2,2	
EMD_HUMAN	117,4		2		14,2	
EF1A1_HUMAN	109,1	125,3	3	4	17,3	19,7
SURF4_HUMAN	102,8		1		6,7	
CALX_HUMAN	100,7		2		4,4	
RS15_HUMAN	92,2	18,1	2	1	28,3	15,2
RS3A_HUMAN	91,8		2		11,7	
RL37A_HUMAN	89,4		1		19,6	
SCPDL_HUMAN	80,3		3		15,4	
ACTB_HUMAN	78,8	473,7	1	9	6,1	39,2
NPM_HUMAN	78,7	74,7	1	1	4,8	4,8
MD2L1_HUMAN	73,2	88,4	1	1	10,2	10,2
SGPL1_HUMAN	72,3		2		5,3	
LANC1_HUMAN	71,8	115,9	1	3	6,5	12,8
HS90B_HUMAN	71,7		2		5,7	
ATPA_HUMAN	68,9	16,7	1	1	2,7	4
H2A1H_HUMAN	66,6		1		14,8	
RS5_HUMAN	65,8		1		7,4	
SC22B_HUMAN	64,7		1		6,5	
DYL1_HUMAN	62,9		1		24,7	
LYRIC_HUMAN	60,9		1		2,7	
SSRD_HUMAN	60,6		2		17,3	
H2B1K_HUMAN	57,9	112,5	1	2	11,9	19
PGRC1_HUMAN	57,6		1		20,5	
ARL1_HUMAN	55,7		1		8,8	
RL40_HUMAN	54,4	20,7	1	1	12,5	12,5
HDAC6_HUMAN	53,6	80,8	2	3	3,8	4,7
AT2A1_HUMAN	52		1		2,3	

DNJA1_HUMAN	49,2	19,3	1	1	6	6
RPN2_HUMAN	48,5		1		1,9	
RS27_HUMAN	47,3	41,7	1	1	15,5	15,5
RS8_HUMAN	46,1		1		6,2	
P53_HUMAN	45,5		1		4,3	
CLH1_HUMAN	44,9		2		1,3	
RL38_HUMAN	39,6	16,7	1	1	18,6	18,6
TMEDA_HUMAN	39,2		1		7,8	
RS10_HUMAN	38,6		1		5,5	
HV366_HUMAN	38	23,7	1	1	32,8	32,8
RS2_HUMAN	38		1		3,8	
RL23_HUMAN	37,9	54,3	1	2	14,3	25
DHB12_HUMAN	36,3		1		4,8	
RS30_HUMAN	36,1	21,2	1	1	16,9	16,9
RAB7A_HUMAN	35,9		1		6,8	
HV372_HUMAN	34,2	31,2	1	1	9,2	9,2
PRKDC_HUMAN	33,6		1		0,5	
RPN1_HUMAN	33,2		2		4,4	
S61A1_HUMAN	31		1		4,8	
DNJA3_HUMAN	30,9		1		4,8	
DNJA2_HUMAN	28,8		1		3,6	
YBOX1_HUMAN	28,5		1		9,3	
RL17_HUMAN	26,8		1		8,7	
K2C1_HUMAN	26,6	29	1	1	1,9	1,9
CPVL_HUMAN	25,2		1		3,6	
RL18_HUMAN	25,2		1		6,9	
AT1A3_HUMAN	24,8		1		2,3	
TCPZ_HUMAN	24,6		1		3,2	
NSDHL_HUMAN	23,2		1		3,5	
RS18_HUMAN	23,1	25	1	1	5,3	5,3
RHG15_HUMAN	21,7		1		2,3	
RS16_HUMAN	20,5		1		9,6	
LPPRC_HUMAN	19,6		1		1,4	
RL15_HUMAN	19		1		6,9	
AKAP1_HUMAN	18,4	18,5	1	1	2,2	2,2
TCPG_HUMAN	18,2		1		2	
RL9_HUMAN	18,2		1		5,2	
TMX1_HUMAN	16,9		1		4,3	
RHG23_HUMAN	16,4	22,6	1	1	0,8	0,8
SVOPL_HUMAN	16,2		1		3,7	
K1C10_HUMAN	15,5	57,1	1	1	4,6	3,3
TCF7_HUMAN	14,5		1		4,7	
C1QBP_HUMAN	14,3	434,3	1	7	10,6	53,5
ADT2_HUMAN	14		1		3	
DOCK5_HUMAN	13,7		1		0,9	
MOB1A_HUMAN	13,6		1		10,2	

Supplementary table S6: Raw mass spectrometry data for GFP-ZMYM4 experiment 1

protein	Scores A GFP-ZMYM4	Scores B GFP	Peptides A	Peptides B	SC (A) [%]	SC (B) [%]
ZMYM4_HUMAN	9656,3		101		64,3	
TBB5_HUMAN	2617,4	2066,3	28	25	74,3	68,7
TBB4B_HUMAN	2339,6	1731,6	25	22	59,8	56,9
TBB4A_HUMAN	2269,9	1573,9	24	19	63,5	53,2
HS71A_HUMAN	1944,9	1000,0	23	13	57,1	34,2
TBA1B_HUMAN	1876,9	1632,4	21	20	70,3	65,2
TBA1A_HUMAN	1818,0	1568,2	20	19	70,3	65,0
HSP7C_HUMAN	1755,9	642,2	23	11	51,9	33,0
CLH1_HUMAN	1754,9	894,9	32	20	35,6	25,2
TBB2A_HUMAN	1747,9		22		53,7	
TBA1C_HUMAN	1686,3	1282,7	19	16	70,2	64,8
DJC10_HUMAN	1682,2	1458,1	16	15	40,4	38,3
HDAC6_HUMAN	1585,4	1349,4	14	14	34,4	32,1
FUBP2_HUMAN	1436,0	424,2	15	10	26,4	26,7
C1QBP_HUMAN	1388,0	1233,7	13	12	62,8	62,8
UBQL4_HUMAN	1266,8		17		69,7	
CH60_HUMAN	1034,7	1424,5	13	19	37,3	41,7
TBB6_HUMAN	1014,9	486,6	15	8	41,7	27,6
PRR12_HUMAN	1003,1		14		28,6	
PYR1_HUMAN	989,2	60,6	19	2	17,3	1,4
TRI32_HUMAN	912,1	219,1	14	6	38,1	21,0
NNRE_HUMAN	758,9	403,4	8	5	55,6	37,8
KDM1A_HUMAN	756,6		14		37,2	
MAGD1_HUMAN	738,3	198,0	8	3	21,9	11,2
PRKDC_HUMAN	737,2	466,9	14	11	6,5	6,4
ZMYM2_HUMAN	710,6		18		30,3	
RU2A_HUMAN	699,9	397,1	8	4	43,5	31,0
DNJA3_HUMAN	689,1	157,8	10	3	28,3	8,3
UBQL1_HUMAN	667,9		8		32,8	
GRP78_HUMAN	667,5	281,6	12	6	32,0	16,8
FBX22_HUMAN	623,5	522,5	8	7	43,4	45,2
DDX3X_HUMAN	618,2	378,0	12	7	27,3	18,9
HNRPU_HUMAN	585,3	188,7	8	3	19,8	7,2
DYL1_HUMAN	584,1	23,5	6	1	50,6	24,7
MATR3_HUMAN	556,2	23,3	8	1	23,0	3,0
ACTB_HUMAN	542,8	390,0	7	5	36,5	24,8
XPO2_HUMAN	537,8	422,8	12	9	27,0	22,7
UBP2L_HUMAN	534,2	736,3	11	10	25,6	20,8
HNRPM_HUMAN	529,8	53,9	9	2	19,7	5,3
CRKL_HUMAN	526,2	233,1	9	5	53,8	41,3
IRS4_HUMAN	514,2	378,3	11	7	25,5	17,2
YYAP1_HUMAN	499,2		9		24,6	
HNRH1_HUMAN	483,6	358,3	7	5	23,6	22,0
FAS_HUMAN	480,1	478,4	11	10	8,3	7,9
DNJA1_HUMAN	473,6	385,3	6	5	35,0	28,7
PEF1_HUMAN	469,0	567,7	5	5	33,8	33,8
EF1A1_HUMAN	451,7	701,2	5	9	24,7	32,7
GRP75_HUMAN	427,7	124,3	8	3	18,6	9,4
PDIA6_HUMAN	424,4	241,5	10	4	36,4	19,3
ATPB_HUMAN	402,7	174,1	10	5	35,3	17,4
SALL2_HUMAN	393,0		5		13,4	
VIME_HUMAN	381,4	74,2	5	2	17,8	7,1
NUCL_HUMAN	380,6	78,0	6	1	14,9	3,1
ROA2_HUMAN	379,9	119,0	3	2	26,9	19,0
PUR6_HUMAN	370,5		8		34,6	
HS90B_HUMAN	361,2	56,5	8	2	19,5	6,4
SC16A_HUMAN	356,4	311,5	5	5	7,3	7,3
HNRPF_HUMAN	353,3	414,0	5	5	26,7	24,1
ZMYM3_HUMAN	348,9		8		15,2	
PCBP1_HUMAN	346,8	355,2	6	5	35,1	34,0
RCN2_HUMAN	346,4	319,0	5	6	31,2	31,5
SYEP_HUMAN	323,2	98,8	6	4	8,5	5,5
PPIA_HUMAN	318,3	212,1	4	3	44,8	36,4
IPO5_HUMAN	312,0	410,0	9	12	15,0	22,4

ZSWM3_HUMAN	310,1		8		19,1	
PIMT_HUMAN	309,1	118,5	5	2	40,5	22,5
DNJA2_HUMAN	303,8	178,3	5	4	27,7	24,0
CALX_HUMAN	293,7	152,9	4	4	15,5	15,5
TNR6B_HUMAN	290,7	111,5	5	3	7,3	6,1
HS90A_HUMAN	287,2	86,0	6	2	18,2	10,8
TRIM5_HUMAN	282,1	248,2	4	6	19,7	20,9
PDCD6_HUMAN	278,1	315,8	4	4	30,4	35,1
IMB1_HUMAN	278,0	121,3	5	4	12,6	10,6
K2C1_HUMAN	275,4	258,8	5	4	16,9	10,4
TCPE_HUMAN	271,5	122,2	3	2	14,0	8,5
H2A1H_HUMAN	271,2	255,5	2	2	37,5	37,5
H2A2C_HUMAN	269,1	241,6	2	2	37,2	37,2
PTBP1_HUMAN	269,0	257,0	5	5	23,7	23,7
DYL2_HUMAN	263,0		3		50,6	
RS3_HUMAN	258,4	69,7	4	1	25,5	9,1
ILVBL_HUMAN	257,2		4		14,6	
DHX9_HUMAN	255,5	17,0	8	1	10,2	1,4
MCM3_HUMAN	248,5	117,9	6	3	10,4	5,7
ATPA_HUMAN	248,3	236,8	5	5	21,9	23,0
RL10_HUMAN	244,9	56,5	3	1	19,2	12,6
CBSL_HUMAN	237,6	252,8	4	4	17,2	21,1
XPO1_HUMAN	234,8	78,8	6	3	11,1	5,5
ROA1_HUMAN	231,5		4		13,4	
AT2A2_HUMAN	228,2	52,5	5	1	9,4	2,5
DNJB6_HUMAN	227,8	92,7	4	3	20,2	15,0
HNRPK_HUMAN	221,7	139,7	5	3	25,9	12,7
IF4A1_HUMAN	214,8	86,3	5	3	22,7	20,0
DDX5_HUMAN	214,4	97,3	6	2	15,8	8,1
RS2_HUMAN	213,9	88,3	5	3	25,6	16,7
HNRH2_HUMAN	212,0	189,7	3	3	13,4	13,4
MCM5_HUMAN	211,6	150,0	5	2	13,8	8,3
RS15_HUMAN	210,0	165,2	2	2	28,3	28,3
RPN1_HUMAN	205,2	41,2	3	2	5,8	5,3
DDX17_HUMAN	205,0	37,2	5	1	8,6	4,5
SC23A_HUMAN	203,7	241,5	5	4	14,1	11,9
RS4X_HUMAN	202,1	82,1	4	3	16,0	9,5
EF1G_HUMAN	200,5	146,0	4	4	19,0	17,6
RSSA_HUMAN	196,0	191,0	3	3	22,7	22,7
RS3A_HUMAN	194,7	79,8	3	2	16,7	11,7
HNRL1_HUMAN	194,0	298,2	5	6	11,9	13,3
KPYM_HUMAN	190,6	178,6	4	4	14,7	15,6
TCPG_HUMAN	190,5	67,9	5	3	20,0	14,3
H2B1K_HUMAN	188,2	148,3	2	2	11,9	11,9
RL23_HUMAN	186,6	147,4	3	3	27,1	27,1
PYRG1_HUMAN	184,7	147,1	3	4	12,0	15,6
SERA_HUMAN	184,6	77,2	6	2	25,5	4,5
RLA1_HUMAN	184,3	90,1	3	2	66,7	51,8
SYRC_HUMAN	181,4	36,5	5	2	11,5	5,3
IF2B1_HUMAN	180,7	57,2	3	1	8,7	2,8
TFR1_HUMAN	176,6	98,5	3	2	11,1	5,8
RS27A_HUMAN	174,8		3		23,1	
RUXE_HUMAN	174,2	80,3	2	1	39,1	27,2
TFG_HUMAN	172,9	34,5	4	2	13,3	13,3
P5CS_HUMAN	172,2	305,3	2	6	5,9	18,0
H4_HUMAN	171,8	67,0	4	1	21,4	11,7
TNPO1_HUMAN	171,2	57,7	6	2	18,5	7,0
TXND5_HUMAN	167,9	96,3	4	2	19,7	12,3
GANAB_HUMAN	159,9		4		10,5	
RL4_HUMAN	159,8		3		11,9	
S61A1_HUMAN	159,4	65,4	2	2	10,1	10,1
K1C10_HUMAN	158,6		2		7,2	
SYIC_HUMAN	156,7	154,5	3	5	4,9	11,2
RLA0_HUMAN	154,1	27,4	2	1	16,7	9,8
YBOX1_HUMAN	153,5	22,9	2	1	14,5	11,1
IPO7_HUMAN	151,0	46,7	3	2	8,7	4,6
TCPZ_HUMAN	148,3	114,7	4	3	13,4	11,1
AT1A1_HUMAN	148,2		2		4,0	

ZSWM1_HUMAN	147,4		3		10,1	
ILF2_HUMAN	144,1	38,7	3	1	20,0	7,2
VPS28_HUMAN	143,8	180,8	4	3	33,0	24,9
MCCB_HUMAN	143,6	88,9	2	2	5,5	6,0
DECR_HUMAN	142,6		2		14,9	
LRC59_HUMAN	142,2	133,5	4	2	24,1	13,4
ZCCHL_HUMAN	142,1	187,3	2	3	18,7	25,0
TRAP1_HUMAN	142,0		2		4,8	
ADNP_HUMAN	141,9		3		6,9	
ACTH_HUMAN	141,5	30,8	2	1	8,5	4,3
NU155_HUMAN	140,3	90,8	3	4	4,1	7,8
RLA2_HUMAN	139,1		3		60,0	
SEC13_HUMAN	138,7	26,7	2	1	12,4	7,1
GCN1_HUMAN	136,6	88,9	5	5	5,1	5,1
DYHC1_HUMAN	135,0	61,4	4	3	1,5	1,4
KT3K_HUMAN	133,4	285,4	3	5	20,7	25,2
HDAC2_HUMAN	132,1		3		10,0	
DDB1_HUMAN	131,9		2		5,4	
HSP74_HUMAN	131,4	33,6	5	1	11,5	1,8
ROA3_HUMAN	129,8		2		4,2	
ESYT1_HUMAN	126,9		4		7,2	
SC24C_HUMAN	123,5	190,2	2	4	4,7	9,4
TIF1B_HUMAN	123,1	67,6	4	3	12,5	13,9
RB11A_HUMAN	122,7	91,9	2	1	19,4	13,4
DHB12_HUMAN	122,3	91,1	2	3	12,8	24,7
TADBP_HUMAN	119,1	148,9	4	4	23,2	23,2
GTF2I_HUMAN	118,8	20,6	5	1	10,9	2,5
PGRC1_HUMAN	117,3	37,0	3	1	27,7	20,5
EF2_HUMAN	116,9	130,0	3	5	5,4	10,6
EMD_HUMAN	116,2	33,8	2	1	17,3	11,4
RCOR1_HUMAN	115,6		2		7,4	
THIL_HUMAN	115,4	85,7	2	2	10,8	10,8
RS5_HUMAN	114,9	72,5	3	1	23,5	7,4
AR6P1_HUMAN	114,7	84,7	1	1	13,8	13,8
GEMI5_HUMAN	113,1		2		3,6	
SKP1_HUMAN	112,9	158,3	2	3	28,8	29,4
1433E_HUMAN	112,5	13,9	3	1	11,4	11,4
RAB14_HUMAN	110,6	26,2	1	1	14,0	14,0
AIMP2_HUMAN	109,5	104,6	2	1	22,5	11,3
K1C9_HUMAN	109,0	241,9	1	4	5,1	20,9
HNRPL_HUMAN	108,9	17,4	1	1	4,9	4,9
PUR4_HUMAN	108,1	109,8	2	2	3,4	3,4
TCPA_HUMAN	107,8	147,6	3	5	14,4	22,5
U520_HUMAN	107,7	17,3	3	1	2,4	0,9
NONO_HUMAN	107,2	111,4	2	2	7,6	9,6
FBRL_HUMAN	106,5		3		15,6	
RS8_HUMAN	103,5		3		19,7	
H2AV_HUMAN	101,2	68,7	1	1	22,7	22,7
ECHB_HUMAN	100,9	130,5	3	4	15,6	23,2
IF5A1_HUMAN	100,6	109,1	2	2	30,5	30,5
RAB10_HUMAN	100,2	28,9	2	1	17,5	5,5
RS12_HUMAN	95,3		2		25,0	
HAX1_HUMAN	95,2	67,2	2	1	17,6	8,6
DCAF7_HUMAN	95,1	187,1	2	1	17,0	12,6
FANCI_HUMAN	94,3		1		2,5	
2AAA_HUMAN	93,4	70,5	2	1	6,5	3,1
SF01_HUMAN	93,4	209,2	3	5	7,8	12,5
RAB7A_HUMAN	92,1	78,1	2	3	15,5	23,2
RS27_HUMAN	91,9		1		15,5	
SIR5_HUMAN	91,2	186,3	2	3	13,9	23,5
ECHA_HUMAN	89,9	278,3	3	5	7,6	16,9
DHE3_HUMAN	89,1		2		6,5	
SYLC_HUMAN	89,0		2		3,1	
ATPO_HUMAN	87,0	47,6	2	2	19,2	18,3
RS24_HUMAN	86,9		1		11,3	
HDAC1_HUMAN	86,0	131,3	3	2	12,9	12,9
SSRG_HUMAN	85,8		1		7,6	
CHD4_HUMAN	85,5		3		5,2	

OPA1_HUMAN	85,0		3		4,5	
COPA_HUMAN	84,8		3		5,8	
DHB4_HUMAN	84,4	62,3	2	1	6,7	3,7
NT5D2_HUMAN	83,3	31,7	2	2	7,3	11,5
RS23_HUMAN	82,9	83,3	2	2	26,6	19,6
PUR2_HUMAN	82,8		1		2,1	
HNRPQ_HUMAN	82,5	30,8	2	1	5,9	2,1
RL7A_HUMAN	82,5	18,1	1	1	6,0	6,0
ECM29_HUMAN	82,4	74,6	1	2	1,6	3,9
PCBP2_HUMAN	80,0	150,0	2	4	9,0	29,0
PARK7_HUMAN	79,8	88,1	2	2	23,8	23,8
EIF3F_HUMAN	79,3	32,0	1	1	5,3	5,3
EWS_HUMAN	79,2	86,0	1	1	7,9	7,9
AP1G1_HUMAN	78,4		2		5,0	
ATPG_HUMAN	77,9	65,1	2	2	8,4	14,8
RAB1A_HUMAN	76,7		2		16,1	
PRP19_HUMAN	75,4		2		14,1	
MCM7_HUMAN	75,3		2		5,8	
K22E_HUMAN	75,2		1		3,6	
SFPQ_HUMAN	75,0	119,1	2	2	5,7	5,7
RL3_HUMAN	74,2	49,0	2	2	6,5	6,5
PUM1_HUMAN	74,0	37,1	1	1	1,7	1,7
SYFA_HUMAN	73,9		2		5,9	
TCPB_HUMAN	72,3	29,1	2	2	7,1	7,3
OST48_HUMAN	71,5	68,5	1	1	6,4	6,4
SIAH1_HUMAN	70,4		2		13,8	
TNR6A_HUMAN	70,0	153,2	1	4	1,3	6,4
HS105_HUMAN	69,6		2		3,1	
BORG5_HUMAN	69,5		2		12,0	
FBX21_HUMAN	68,8		1		3,7	
RAN_HUMAN	68,4	118,1	2	3	29,6	29,6
RPN2_HUMAN	67,8	100,6	3	3	11,4	12,4
SAC1_HUMAN	67,5		1		6,0	
SYPM_HUMAN	67,3	122,4	3	4	10,1	14,1
PSA5_HUMAN	67,1		2		15,8	
MOGS_HUMAN	67,1		2		3,6	
CS068_HUMAN	66,6		1		2,2	
DJB11_HUMAN	66,2		1		4,5	
BCKD_HUMAN	66,1		2		9,2	
SYTM_HUMAN	66,0		2		7,1	
CHCH9_HUMAN	64,5		1		18,5	
TS101_HUMAN	64,2	151,5	2	5	11,8	20,0
ZBTB1_HUMAN	63,7		1		2,4	
NPM_HUMAN	63,7		1		4,8	
RUVB1_HUMAN	63,5		2		8,8	
KCRB_HUMAN	61,6		2		8,7	
YETS4_HUMAN	61,1	165,0	1	1	11,9	11,9
ACSL3_HUMAN	60,8		1		2,4	
YTHD2_HUMAN	60,7		1		5,9	
NFAC1_HUMAN	60,4	49,0	1	1	4,5	4,5
SFXN1_HUMAN	60,2	43,6	1	1	10,6	10,6
RS16_HUMAN	59,8	16,7	1	1	6,8	6,8
HSPB1_HUMAN	59,3		1		8,3	
RS10_HUMAN	59,2	36,4	2	1	14,5	5,5
TIM50_HUMAN	58,9	40,4	2	1	7,9	4,8
HNRPC_HUMAN	58,8		1		3,6	
RBM14_HUMAN	58,6	66,5	2	2	5,8	10,5
DHX15_HUMAN	58,6	81,1	2	1	5,9	4,4
CBX3_HUMAN	57,7		1		8,7	
ILF3_HUMAN	57,5		1		3,8	
RS28_HUMAN	57,4		1		17,4	
ZN281_HUMAN	57,4	25,4	1	1	3,4	3,4
HV366_HUMAN	57,1	36,1	1	1	19,8	19,8
RS26_HUMAN	57,1	47,2	1	1	13,0	13,0
4F2_HUMAN	56,9	33,7	1	1	3,3	1,9
SSRD_HUMAN	56,4	15,9	2	1	17,3	11,0
RL31_HUMAN	56,3	63,1	1	2	11,2	18,4
CARL1_HUMAN	55,7		1		2,1	

RTN4_HUMAN	55,1	15,5	1	1	2,9	2,9
IMA1_HUMAN	54,9		2		9,1	
LAP2B_HUMAN	54,2	36,4	3	1	14,5	5,1
RL11_HUMAN	54,2		1		7,9	
SYK_HUMAN	53,8	16,7	2	1	7,2	5,7
SRPRB_HUMAN	53,7	65,5	2	2	12,2	7,4
MSD2_HUMAN	53,7		1		2,9	
NCLN_HUMAN	53,3	33,6	1	1	5,7	5,7
TBCD_HUMAN	53,2		1		3,5	
NU188_HUMAN	52,8		1		1,7	
RL37A_HUMAN	52,6	65,2	1	1	19,6	19,6
RS13_HUMAN	52,5		3		18,5	
MDN1_HUMAN	52,2		1		0,5	
SMU1_HUMAN	52,0	65,5	1	1	3,3	3,3
RL17_HUMAN	51,8		1		8,7	
NDUA4_HUMAN	51,5		1		14,8	
PP1G_HUMAN	51,4	72,1	1	1	5,3	5,3
VP37B_HUMAN	50,9	249,1	2	5	13,3	37,2
1433Z_HUMAN	50,5		2		11,8	
COPB2_HUMAN	50,5	31,7	3	1	6,5	2,1
UBA1_HUMAN	50,3	60,8	2	1	5,4	3,2
S23IP_HUMAN	50,1	52,7	1	1	3,6	3,6
QCR2_HUMAN	49,8	25,4	2	1	8,2	11,0
RFOX2_HUMAN	49,3	29,6	2	1	10,5	6,7
BASI_HUMAN	49,3		1		4,7	
SF3B6_HUMAN	48,8		1		9,6	
SC22B_HUMAN	48,2		1		5,6	
CAPR1_HUMAN	48,0		1		5,9	
TCPD_HUMAN	47,9	43,8	3	1	10,2	4,8
IF4A3_HUMAN	47,5		1		5,1	
SC24A_HUMAN	47,0	41,6	1	1	1,4	1,4
TARB1_HUMAN	46,9		1		1,9	
CPVL_HUMAN	46,2	21,1	1	1	7,4	7,4
RS17_HUMAN	45,7	40,9	1	1	16,3	16,3
CN166_HUMAN	45,5		1		5,7	
SMRC1_HUMAN	44,8	20,8	2	1	5,9	1,7
HNRPD_HUMAN	44,3		1		2,3	
RL24_HUMAN	44,2		1		8,3	
RACK1_HUMAN	43,8	13,8	1	1	8,5	7,6
MPCP_HUMAN	43,8		1		4,4	
IRAK1_HUMAN	43,0	188,4	1	4	3,1	18,0
AAAT_HUMAN	42,9		1		2,0	
TMX1_HUMAN	42,7		1		4,3	
RL18_HUMAN	42,7		1		6,9	
DESP_HUMAN	42,4	38,1	2	1	1,5	0,9
RS19_HUMAN	42,4		1		9,0	
CAPZB_HUMAN	42,4	21,1	2	1	16,2	9,0
EIF3E_HUMAN	42,2	18,7	1	1	4,0	4,0
PSB1_HUMAN	42,1	36,3	2	1	18,7	11,2
GFPT2_HUMAN	41,8	52,1	1	1	3,2	3,2
MIC13_HUMAN	41,6	75,6	1	1	16,9	16,9
GET4_HUMAN	41,4	49,7	1	1	11,6	11,6
TMM33_HUMAN	41,4		1		4,9	
HLAE_HUMAN	41,2		1		3,9	
U5S1_HUMAN	40,8		1		1,7	
TMEDA_HUMAN	40,7		1		5,0	
IPO11_HUMAN	40,6		1		3,4	
THOC4_HUMAN	40,1		2		17,5	
PRS10_HUMAN	40,1		1		4,4	
HACD3_HUMAN	40,0		1		2,8	
SYMC_HUMAN	39,8	55,4	1	1	3,1	3,1
PARP1_HUMAN	39,4	36,2	2	2	4,2	4,8
AAAS_HUMAN	39,3	13,0	2	1	7,7	4,2
RL35_HUMAN	39,1	29,1	1	1	10,6	10,6
RIR1_HUMAN	38,9	24,5	1	1	2,9	2,9
FUS_HUMAN	38,9	50,5	2	2	9,3	9,3
SF3B3_HUMAN	38,7	29,0	1	1	3,0	3,0
CEPT1_HUMAN	38,3	48,6	1	1	3,8	3,8

VAS1_HUMAN	38,2		1		8,3	
RBM39_HUMAN	38,1		2		4,7	
STT3A_HUMAN	38,1	26,5	1	1	3,0	3,0
C1TC_HUMAN	38,0	20,4	1	1	1,7	2,8
SCAM3_HUMAN	38,0		1		4,0	
ANM1_HUMAN	38,0		2		8,3	
ASNA_HUMAN	37,1	21,1	1	1	9,8	9,8
SURF4_HUMAN	37,1	76,3	1	1	6,7	6,7
FAF2_HUMAN	37,0	19,5	1	1	6,5	6,5
CNN3_HUMAN	37,0		1		14,3	
AKP8L_HUMAN	36,4	17,6	1	1	3,6	4,3
ASPH_HUMAN	36,2		1		2,4	
PPP6_HUMAN	35,9	38,8	1	1	7,2	7,2
ADT2_HUMAN	35,6		1		4,4	
ALDR_HUMAN	35,3	16,7	1	1	11,1	11,1
QPCTL_HUMAN	35,3		1		9,2	
ELOC_HUMAN	35,0	69,2	1	1	26,8	26,8
CEP44_HUMAN	35,0		1		6,7	
PSB3_HUMAN	34,0		1		8,8	
XPO5_HUMAN	33,9	100,6	1	2	2,8	5,0
THOC3_HUMAN	33,7		1		2,8	
MTMR3_HUMAN	33,5	20,4	1	1	2,2	2,2
SYDC_HUMAN	33,5	49,8	2	2	7,8	7,4
ACSL1_HUMAN	33,3		1		3,7	
PHB_HUMAN	33,2	29,2	1	1	7,4	7,4
C1TM_HUMAN	33,0		1		1,0	
UBP24_HUMAN	32,5		1		1,1	
NOL9_HUMAN	32,5		1		5,6	
ACOD_HUMAN	32,3	23,1	1	1	7,0	7,0
TPR_HUMAN	32,2		1		1,0	
COPB_HUMAN	31,5		1		2,2	
KAT2A_HUMAN	31,2		1		4,4	
PO210_HUMAN	30,6	14,5	1	1	1,3	1,3
TAGL2_HUMAN	30,5	67,7	1	2	9,0	18,6
RL19_HUMAN	30,3		1		8,7	
MDHM_HUMAN	30,2		1		3,3	
LMAN1_HUMAN	30,1		1		4,1	
CCD47_HUMAN	29,6	36,8	1	1	5,0	3,7
SPTC1_HUMAN	29,5	25,3	1	1	7,2	7,2
RHG35_HUMAN	29,5		1		2,1	
SIX1_HUMAN	29,0		1		4,6	
MBB1A_HUMAN	29,0		1		2,5	
MD2L1_HUMAN	28,5	57,0	1	1	10,2	10,2
ELAV1_HUMAN	28,3		1		7,4	
RL30_HUMAN	28,1	43,9	1	1	13,9	13,9
SPD2A_HUMAN	28,1	43,3	1	2	1,9	1,9
NUBP2_HUMAN	27,9	41,1	1	1	9,6	9,6
H32_HUMAN	27,9		1		23,5	
AIMP1_HUMAN	27,8		1		10,9	
CD81_HUMAN	27,7	33,4	1	1	9,7	9,7
API5_HUMAN	27,0	23,1	1	1	5,5	5,5
RDH11_HUMAN	26,9		1		6,3	
KIF22_HUMAN	26,9		1		2,0	
P5CR1_HUMAN	26,7	33,7	1	1	14,7	14,7
SYIM_HUMAN	26,7		1		3,6	
PCAT1_HUMAN	26,5		1		2,2	
ARL1_HUMAN	26,4	28,0	1	1	8,8	8,8
RUVB2_HUMAN	25,8		1		3,5	
SC24B_HUMAN	25,7	33,0	1	1	1,5	1,5
STML2_HUMAN	25,6		1		8,4	
RS30_HUMAN	25,5		1		16,9	
MYCB2_HUMAN	25,3		1		0,2	
ATPK_HUMAN	25,2		1		11,7	
ITPR3_HUMAN	25,2		1		0,5	
RCBT1_HUMAN	24,9		1		6,0	
RSU1_HUMAN	24,7		1		12,3	
MAGD2_HUMAN	24,6		1		2,6	
COPZ1_HUMAN	24,5	15,3	1	1	18,6	18,6

RAB21_HUMAN	24,4		1		7,6	
PRAF3_HUMAN	24,3		1		5,9	
COPE_HUMAN	24,3		1		4,5	
RCC2_HUMAN	23,9		1		2,1	
SDHA_HUMAN	23,7	56,9	1	2	3,3	8,4
PSD12_HUMAN	23,7		1		3,1	
RL6_HUMAN	23,6		1		5,9	
USP9X_HUMAN	23,5		1		1,5	
DC1L1_HUMAN	23,4		1		3,6	
HDHD5_HUMAN	23,1		1		6,4	
DRG1_HUMAN	23,0	15,2	1	1	8,2	8,2
POGZ_HUMAN	23,0		1		2,4	
PSMD1_HUMAN	22,7		1		1,3	
NU205_HUMAN	22,6		1		1,1	
LMAN2_HUMAN	22,4	15,1	1	1	7,3	5,9
NH2L1_HUMAN	22,3		1		21,9	
RM03_HUMAN	22,0	36,3	1	1	10,3	10,3
DDX20_HUMAN	22,0		1		3,8	
SRP14_HUMAN	21,8		1		10,3	
SF3B2_HUMAN	21,5		1		2,6	
PSDE_HUMAN	21,4		1		11,0	
TTC7B_HUMAN	21,4	21,2	1	1	2,4	2,4
HNRH3_HUMAN	21,3	37,0	1	1	4,9	4,9
ALBU_HUMAN	21,3		1		2,5	
TMED5_HUMAN	21,0		1		10,0	
HV353_HUMAN	21,0	18,7	1	1	19,8	19,8
UGGG1_HUMAN	20,9	123,4	1	4	1,6	7,6
PSMD4_HUMAN	20,9		1		15,4	
TRI65_HUMAN	20,9	21,2	1	1	4,1	4,3
LYRM4_HUMAN	20,7		1		8,8	
G6PI_HUMAN	20,5		1		2,7	
VP37A_HUMAN	20,5	31,7	1	1	10,1	10,1
WDR19_HUMAN	20,4		1		1,1	
THIO_HUMAN	20,1		1		8,6	
SSRP1_HUMAN	20,0		1		3,0	
HDAC7_HUMAN	20,0		1		0,8	
LDHA_HUMAN	19,9	32,4	1	1	5,1	5,1
VDAC2_HUMAN	19,9		1		6,8	
UTP20_HUMAN	19,8	19,9	1	1	0,6	0,6
U2AF2_HUMAN	19,7		1		5,1	
BAG2_HUMAN	19,5		1		5,2	
NSDHL_HUMAN	19,4		1		4,3	
GOGA4_HUMAN	18,9		1		1,2	
INT1_HUMAN	18,9		1		1,4	
RMXL1_HUMAN	18,8		1		3,6	
CELF1_HUMAN	18,7		1		7,4	
KCRU_HUMAN	18,6	25,5	1	1	6,2	6,2
VAPA_HUMAN	18,5		1		7,6	
H2AY_HUMAN	18,4		1		7,8	
CTND1_HUMAN	18,3		1		3,8	
PSB7_HUMAN	18,3		1		6,9	
FA98A_HUMAN	18,0		1		3,3	
RAB5C_HUMAN	17,8	15,8	1	1	11,1	11,1
TIM44_HUMAN	17,8		1		2,9	
NP1L1_HUMAN	17,7	41,2	1	2	2,8	7,2
SMD3_HUMAN	17,5		1		7,1	
RL21_HUMAN	17,3		1		11,3	
RM12_HUMAN	17,2		1		12,1	
PYGL_HUMAN	17,0		1		5,3	
CILP1_HUMAN	16,9		1		1,3	
TM209_HUMAN	16,9		1		4,1	
TIRR_HUMAN	16,7	41,8	1	1	4,7	8,1
ROAA_HUMAN	16,7		1		2,4	
EIF3A_HUMAN	16,7	18,9	1	1	1,4	1,4
NCHL1_HUMAN	16,6		1		0,9	
CYB5B_HUMAN	16,6		1		23,3	
K1107_HUMAN	16,6		1		1,5	
HV311_HUMAN	16,5	26,5	1	1	19,7	19,7

RBP56_HUMAN	16,3		1		5,7	
CNIH4_HUMAN	16,2		1		14,4	
TM9S2_HUMAN	16,1		1		2,3	
PERM_HUMAN	16,1	15,9	1	1	3,9	3,9
PSMD6_HUMAN	16,0	13,8	1	1	6,2	6,2
SPEF2_HUMAN	16,0		1		0,6	
TM39B_HUMAN	15,9		1		2,4	
MTHFS_HUMAN	15,8		1		4,4	
CDK4_HUMAN	15,7	14,0	1	1	9,2	9,2
FOXP1_HUMAN	15,7		1		4,9	
RBM4_HUMAN	15,6		1		7,7	
TCPQ_HUMAN	15,6	14,0	1	1	2,9	3,3
MCMBP_HUMAN	15,5	51,2	1	2	3,9	10,6
ENOA_HUMAN	15,3		1		3,9	
LRP8_HUMAN	15,2		1		1,0	
TBR1_HUMAN	15,1		1		1,8	
RS9_HUMAN	14,9		1		5,7	
MYZAP_HUMAN	14,8		1		5,2	
AL3A2_HUMAN	14,8		1		2,9	
KIF1B_HUMAN	14,7		1		0,6	
UBP15_HUMAN	14,7		1		1,4	
MMGT1_HUMAN	14,7		1		18,3	
BZW2_HUMAN	14,6		1		1,9	
PNML1_HUMAN	14,6		1		3,2	
PR38B_HUMAN	14,6		1		4,6	
RND2_HUMAN	14,5		1		3,1	
CNTD2_HUMAN	14,5		1		4,9	
AUP1_HUMAN	14,4		1		5,3	
GPN1_HUMAN	14,4		1		9,4	
CGNL1_HUMAN	14,1		1		1,3	
NRBP2_HUMAN	14,0		1		3,8	
CLPB_HUMAN	13,9		1		2,1	
PRC2B_HUMAN	13,9		1		0,5	
AT1B3_HUMAN	13,9		1		5,4	
LMNB1_HUMAN	13,8		1		4,1	
GLD2_HUMAN	13,8		1		2,1	
CDC42_HUMAN	13,8		1		8,9	
C2TA_HUMAN	13,5		1		2,1	
DHX30_HUMAN	13,5		1		1,5	
GGYF1_HUMAN	13,4		1		0,8	
RCC1L_HUMAN	13,4		1		3,0	
DOCK3_HUMAN	13,4		1		0,4	
RAD17_HUMAN	13,3		1		2,9	
CYTB_HUMAN	13,3		1		21,4	
PROF1_HUMAN	13,1		1		11,4	
ARAF_HUMAN	13,0		1		2,3	

Supplementary table S7: Raw mass spectrometry data for GFP-ZMYM4 experiment 2

protein	Scores A GFP-ZMYM4	Scores B GFP	Peptides A	Peptides B	SC (A) [%]	SC (B) [%]
ZMYM4_HUMAN	9700,8		103		65,4	
TBB5_HUMAN	2644,3	1441,9	28	20	76,8	72,7
TBB4B_HUMAN	2371,3	1263,7	25	17	73,9	54,8
CLH1_HUMAN	2248,8	922,9	43	19	44,2	21,4
TBB4A_HUMAN	2211,6		22		61,9	
TBB2B_HUMAN	1815,9	957,4	23	14	56,0	41,6
TBA1A_HUMAN	1543,4	1154,5	20	14	73,4	55,2
DJC10_HUMAN	1538,8	1290,2	18	15	39,7	36,3
TBA1B_HUMAN	1505,7	1147,3	20	14	73,4	55,2
HSP7C_HUMAN	1441,3	621,3	23	11	51,7	28,8
HS71A_HUMAN	1437,8	829,4	21	13	55,4	34,8
TBA1C_HUMAN	1376,2	1005,7	19	13	73,3	50,8
FUBP2_HUMAN	1327,9	1023,3	17	15	37,6	31,9
C1QBP_HUMAN	1166,9	835,4	11	9	62,8	55,7
HDAC6_HUMAN	1091,3	833,9	16	14	36,5	30,4
TBB6_HUMAN	1037,9		16		56,1	
PRR12_HUMAN	1000,8		14		25,0	
ZMYM2_HUMAN	936,4		20		29,6	
TRI32_HUMAN	925,8	70,7	15	3	40,4	8,0
PYR1_HUMAN	912,3	39,7	20	1	20,1	0,9
DECR_HUMAN	877,8		14		57,3	
RU2A_HUMAN	871,2	464,9	10	7	56,9	48,2
HNRPU_HUMAN	782,6	416,4	12	6	27,0	14,3
KDM1A_HUMAN	707,2	17,2	15	1	40,3	3,2
NNRE_HUMAN	645,2	316,5	8	5	55,6	38,5
YYAP1_HUMAN	605,9		12		34,7	
DDX3X_HUMAN	601,9	695,3	12	12	27,3	28,2
GRP78_HUMAN	579,6	243,9	9	4	24,2	9,6
MAGD1_HUMAN	543,0	348,2	7	6	21,2	20,2
PEF1_HUMAN	536,4	561,3	5	5	33,8	33,8
HNRH1_HUMAN	534,3	537,7	7	8	22,3	33,6
EF1A1_HUMAN	513,3		7		32,5	
IRS4_HUMAN	512,8	135,5	11	4	23,4	10,6
HNRPF_HUMAN	480,2	369,6	7	7	31,1	34,2
AP1G1_HUMAN	470,2		8		22,1	
DNJA3_HUMAN	467,3	94,0	10	2	28,1	9,6
SYIC_HUMAN	462,3	16,9	9	1	13,3	2,6
FBX22_HUMAN	461,1	314,5	8	7	48,1	38,5
DYL1_HUMAN	459,7	83,7	4	1	38,2	24,7
ZSWM3_HUMAN	453,0		12		26,7	
ROA2_HUMAN	407,9	269,3	6	4	37,7	34,0
FAS_HUMAN	406,1	517,3	8	13	5,6	8,9
SC16A_HUMAN	401,3	510,5	9	9	11,2	10,3
ZMYM3_HUMAN	400,1		10		13,3	
HNRPM_HUMAN	394,1		8		17,7	
PDIA6_HUMAN	389,9	416,8	8	8	29,1	32,0
TNR6B_HUMAN	389,6	155,9	9	4	11,4	6,1
CRKL_HUMAN	384,3	362,6	8	7	53,5	49,8
DHX9_HUMAN	381,6	104,1	11	2	15,5	2,5
SC23A_HUMAN	375,8	433,8	7	8	17,9	20,9
PDCD6_HUMAN	374,0	480,4	6	7	45,0	57,6
SYEP_HUMAN	342,3	38,4	6	2	7,7	2,2
PCBP1_HUMAN	330,7	543,2	7	8	38,8	43,0
SYRC_HUMAN	330,3	114,9	8	3	20,0	5,3
UBP2L_HUMAN	326,7	659,2	6	12	14,1	26,0
MATR3_HUMAN	325,6	167,0	5	2	13,7	5,1
DDX5_HUMAN	325,6	330,1	6	9	15,8	19,9
IPO5_HUMAN	308,4	420,8	9	10	15,7	16,3
MCCB_HUMAN	303,1	164,3	5	3	13,5	8,3
HNRL1_HUMAN	297,9	453,0	8	9	16,9	19,3
NUCL_HUMAN	293,4	99,9	6	2	14,8	5,1
ILVBL_HUMAN	291,5		6		27,2	
MCM3_HUMAN	288,1	238,5	8	7	14,5	12,3
MCM5_HUMAN	287,3	312,4	5	6	11,9	15,4

ACTB_HUMAN	284,7	346,8	4	4	16,3	24,0
VIME_HUMAN	284,7	230,3	4	4	14,8	13,1
CBSL_HUMAN	269,3	301,3	4	4	17,2	18,7
SALL2_HUMAN	264,9		4		10,1	
CH60_HUMAN	262,6	444,0	4	6	20,2	19,5
HNRPK_HUMAN	261,2	149,3	5	3	25,9	12,7
PPIA_HUMAN	255,6	188,4	4	4	44,8	41,2
CHD4_HUMAN	255,3		7		8,5	
H2A2C_HUMAN	253,6		2		37,2	
CS068_HUMAN	252,8		7		19,1	
NU155_HUMAN	252,5	275,3	6	7	9,4	10,3
TARB1_HUMAN	248,3	19,7	6	1	6,4	1,9
PYRG1_HUMAN	246,9	280,8	4	6	14,0	19,5
NONO_HUMAN	242,4	66,2	4	1	14,4	4,9
RS2_HUMAN	240,3	157,5	6	4	25,9	19,1
PTBP1_HUMAN	239,8	257,0	6	5	31,1	23,7
HNRH2_HUMAN	237,4	327,6	4	5	19,2	22,0
H2A1H_HUMAN	236,2		2		37,5	
TRI65_HUMAN	230,6	74,9	5	2	24,8	8,3
SFPQ_HUMAN	227,9	91,6	4	1	10,2	3,3
DDX17_HUMAN	224,8	302,3	4	7	6,9	11,8
UBQL4_HUMAN	220,9		5		25,1	
PRKDC_HUMAN	218,7	81,0	6	1	3,2	0,5
HNRPL_HUMAN	217,9	131,9	4	1	13,2	4,9
AIMP2_HUMAN	214,8	79,9	3	1	21,9	11,3
DNJA1_HUMAN	205,6	31,0	6	2	30,7	9,3
IF2B1_HUMAN	202,3		4		10,9	
RA1L2_HUMAN	201,4	161,5	2	2	8,1	8,1
SC24C_HUMAN	200,3	238,5	6	6	12,1	9,9
H2AV_HUMAN	198,0	264,8	1	2	22,7	29,7
PIMT_HUMAN	195,1	79,3	4	1	33,9	7,5
SIR5_HUMAN	192,0	169,4	4	3	19,4	16,5
RL23_HUMAN	191,8	97,6	3	2	27,1	25,0
ZSWM1_HUMAN	189,5		5		14,2	
YBOX1_HUMAN	189,4	84,3	3	1	20,4	5,2
TS101_HUMAN	187,6	210,1	5	5	18,2	22,3
VPS28_HUMAN	185,6	228,6	4	3	33,0	20,4
SC24B_HUMAN	177,5	191,7	3	3	5,2	5,8
ILF2_HUMAN	176,4	142,0	4	4	26,9	26,9
RS3_HUMAN	175,6	195,5	4	6	25,5	34,6
GEMI5_HUMAN	175,3		3		4,2	
TRIM5_HUMAN	167,6	132,4	2	1	7,1	3,4
RS8_HUMAN	163,8	197,0	4	4	26,4	25,5
ACTH_HUMAN	162,8	58,8	2	2	8,5	8,5
DHE3_HUMAN	161,0		2		7,0	
ZCCHL_HUMAN	160,7	202,0	2	3	18,7	26,0
HNRPC_HUMAN	160,0		3		12,7	
GRP75_HUMAN	159,3		4		10,0	
SYMC_HUMAN	158,7	78,1	3	1	9,2	3,1
H2B1J_HUMAN	156,2	289,9	2	4	19,0	26,2
RS15_HUMAN	156,1	177,9	2	2	28,3	28,3
C1TC_HUMAN	155,9	237,2	4	5	9,3	12,1
H4_HUMAN	155,8	284,7	4	5	21,4	47,6
RUXE_HUMAN	155,1		3		56,5	
RL10_HUMAN	152,7	104,1	3	2	19,2	18,7
RS27A_HUMAN	152,1	156,4	2	3	22,4	30,8
RS3A_HUMAN	151,5	111,5	3	2	16,7	11,7
ADNP_HUMAN	149,9		3		6,9	
HS90B_HUMAN	149,3		2		4,0	
C1TM_HUMAN	148,7	97,8	6	2	12,4	3,2
RS4X_HUMAN	148,1	85,0	3	1	17,5	6,5
RS5_HUMAN	141,3	93,3	3	2	23,5	17,2
SYDC_HUMAN	134,2	44,1	2	2	6,0	7,0
PCBP2_HUMAN	131,6	147,3	2	3	9,0	13,4
ROA3_HUMAN	131,4		1		4,2	
RS24_HUMAN	129,0	91,7	2	2	20,3	20,3
SEC13_HUMAN	126,9		2		12,4	
GTF2I_HUMAN	125,7		4		8,7	

RCOR1_HUMAN	125,4		3		10,3	
RIR1_HUMAN	125,1		3		8,8	
RSSA_HUMAN	123,5	204,1	2	2	14,9	14,9
EWS_HUMAN	117,6	149,7	2	2	11,6	11,6
SC23B_HUMAN	115,2		4		6,9	
POGZ_HUMAN	114,4		2		4,9	
FUS_HUMAN	111,4	26,6	2	1	9,3	6,1
TADBP_HUMAN	111,4	38,2	3	1	17,4	5,8
THIL_HUMAN	107,7	49,6	3	1	13,8	4,0
IRAK1_HUMAN	107,0	242,4	3	5	7,6	15,2
MCMBP_HUMAN	105,4	28,0	2	1	7,6	3,9
DYL2_HUMAN	103,5		3		27,0	
SYPM_HUMAN	102,7	218,2	3	6	11,2	23,6
RBM39_HUMAN	101,7	80,1	3	2	7,9	5,3
NT5D2_HUMAN	101,4	204,1	3	5	10,4	15,0
RLA1_HUMAN	101,0		2		51,8	
PDC6I_HUMAN	98,5	470,3	2	10	7,3	21,7
HNRPR_HUMAN	97,4		3		13,6	
TNR6A_HUMAN	97,1	47,3	3	1	3,9	1,2
XRN2_HUMAN	96,7	974,8	2	18	5,3	34,0
P5CS_HUMAN	96,6	139,0	1	3	3,0	6,5
HNRPQ_HUMAN	96,3	26,6	2	1	5,9	2,1
SYLC_HUMAN	96,2		2		3,1	
KT3K_HUMAN	95,7	142,8	3	3	20,7	19,7
AGO2_HUMAN	94,0		1		2,0	
DHB4_HUMAN	92,8	30,9	2	1	5,7	3,4
RL7A_HUMAN	91,2		3		13,9	
TCPZ_HUMAN	90,7		2		6,2	
K1C10_HUMAN	83,7	106,1	2	1	7,2	3,3
RL19_HUMAN	83,6	60,4	1	1	8,7	8,7
DCAF7_HUMAN	81,5	214,0	3	4	15,5	28,1
CARL1_HUMAN	79,6		1		2,1	
SIX1_HUMAN	76,7		2		10,9	
SYTM_HUMAN	75,4		2		4,3	
HDAC1_HUMAN	74,6	48,5	3	1	14,1	2,5
UGGG1_HUMAN	74,0	26,2	2	1	3,4	0,9
RL4_HUMAN	72,7	58,9	1	2	5,2	8,0
HDAC2_HUMAN	72,3		2		6,1	
RCN2_HUMAN	72,2		1		7,3	
RS23_HUMAN	70,7	138,7	2	2	26,6	26,6
KITH_HUMAN	69,9	70,7	1	1	15,0	15,0
RL31_HUMAN	68,2	55,3	1	1	11,2	11,2
YETS4_HUMAN	67,9	62,5	1	1	11,9	11,9
ROA0_HUMAN	67,3		2		11,8	
RS17_HUMAN	67,1	66,6	2	2	32,6	32,6
RL7_HUMAN	66,9	51,6	2	2	11,7	11,7
CAPR1_HUMAN	66,7		1		5,9	
UBP15_HUMAN	66,2		2		4,2	
UBR5_HUMAN	65,8		2		1,4	
PUR4_HUMAN	65,0	61,4	1	1	2,0	2,0
TCPE_HUMAN	64,4		1		5,4	
SF01_HUMAN	62,6	151,9	2	3	5,8	7,8
DNJA2_HUMAN	62,2		2		10,9	
SMRC1_HUMAN	62,0		2		5,9	
CAPZB_HUMAN	61,1	41,4	1	1	9,0	9,0
MSD2_HUMAN	61,0		2		7,9	
PABP1_HUMAN	60,9	81,4	2	3	6,3	5,8
RS16_HUMAN	59,8	84,7	1	2	6,8	14,4
PSA5_HUMAN	59,4		1		7,9	
TXND5_HUMAN	59,2	108,6	1	3	4,6	14,4
RBM14_HUMAN	59,2	99,9	2	2	8,5	7,3
VP37B_HUMAN	59,0	220,0	2	4	15,8	28,1
RS27_HUMAN	58,7	45,7	1	1	15,5	15,5
S61A2_HUMAN	58,1		1		5,3	
PRP19_HUMAN	57,0		2		14,1	
EF1G_HUMAN	56,4		2		7,3	
RCC2_HUMAN	56,2	276,3	2	5	6,1	14,9
MMTA2_HUMAN	56,2	119,1	2	3	9,1	14,1

EPIPL_HUMAN	55,1		1		0,5	
SIAH1_HUMAN	52,8		1		6,0	
NOL9_HUMAN	52,4		1		5,6	
RS26_HUMAN	52,2	45,4	1	1	13,0	13,0
MB12A_HUMAN	52,1	169,2	2	4	11,0	23,4
IF4A3_HUMAN	51,2		1		5,1	
RL11_HUMAN	50,9	50,9	1	1	7,9	7,9
DYHC1_HUMAN	49,8		1		0,3	
RAN_HUMAN	48,4	24,1	2	1	29,6	6,5
PALLD_HUMAN	48,0	104,6	1	3	2,2	5,8
PARP1_HUMAN	48,0	222,8	1	5	1,5	11,8
EIF3F_HUMAN	47,9		1		5,3	
AIMP1_HUMAN	47,5		1		10,9	
XPO2_HUMAN	47,4	40,1	1	1	3,0	3,0
RHG35_HUMAN	47,1		1		2,1	
IMB1_HUMAN	46,7	20,3	1	1	3,5	3,5
K2C1_HUMAN	45,5	324,9	1	4	6,1	9,8
FA98A_HUMAN	45,5	15,5	1	1	3,3	3,3
DESP_HUMAN	44,7	46,8	2	2	0,9	0,9
NKRF_HUMAN	44,6	53,2	1	1	3,6	3,6
UBQL1_HUMAN	44,0		2		5,3	
NUBP2_HUMAN	43,7	71,9	1	2	9,6	15,1
SYFA_HUMAN	43,6		1		3,1	
ILF3_HUMAN	42,7	17,7	1	1	3,8	2,0
RLA2_HUMAN	42,5		1		28,7	
MBB1A_HUMAN	42,4	21,1	1	1	2,5	1,4
RL6_HUMAN	42,0	61,2	1	2	5,9	9,4
IF5AL_HUMAN	41,4	16,3	1	1	15,6	15,6
MD2L1_HUMAN	41,0	34,7	1	1	10,2	10,2
RL15_HUMAN	40,8	44,5	1	1	6,9	6,9
GFPT2_HUMAN	40,7	23,7	1	1	3,2	3,2
HS105_HUMAN	40,6		2		4,1	
NPM_HUMAN	40,5	93,1	1	1	12,6	4,8
SMRC2_HUMAN	40,1		1		3,8	
PUR6_HUMAN	39,8		1		8,5	
HLTF_HUMAN	39,6	88,7	1	2	1,9	4,7
RRP5_HUMAN	39,3	36,7	1	1	2,3	2,3
TCPA_HUMAN	38,7		1		4,9	
THOC4_HUMAN	38,3		2		17,5	
RL17_HUMAN	37,6		1		8,7	
NOP56_HUMAN	36,3		2		8,1	
NH2L1_HUMAN	35,7		1		21,9	
RL30_HUMAN	35,1	55,1	1	1	13,9	13,9
RL24_HUMAN	34,1	48,6	1	1	8,3	5,7
QPCTL_HUMAN	34,1		1		9,2	
RS10_HUMAN	33,9	22,5	1	1	5,5	5,5
ERF3A_HUMAN	32,6	17,4	1	1	5,2	5,2
BOP1_HUMAN	32,6		1		4,2	
MYCB2_HUMAN	32,5		1		0,6	
RMXL1_HUMAN	32,1		1		3,6	
MCA3_HUMAN	31,1		1		14,4	
RL18_HUMAN	30,9	21,0	1	1	6,9	6,9
HSP74_HUMAN	30,9		1		4,3	
FBRL_HUMAN	30,2		1		6,2	
THOC3_HUMAN	30,0		1		2,8	
RL35_HUMAN	29,4		1		10,6	
DDX1_HUMAN	29,3	33,5	1	1	2,8	2,8
KHDR1_HUMAN	29,3		1		9,0	
SYK_HUMAN	28,8		1		1,8	
IF4A1_HUMAN	28,7	14,1	1	1	5,2	5,7
RL3_HUMAN	28,7		1		6,0	
NU205_HUMAN	27,9		1		1,1	
PTCD3_HUMAN	27,6		1		2,2	
SF3B3_HUMAN	26,9		1		3,0	
PAN2_HUMAN	26,9		1		4,2	
PP1G_HUMAN	26,6	39,3	1	1	5,3	5,3
PR38B_HUMAN	26,3	37,3	1	1	4,6	4,6
ALBU_HUMAN	26,3	151,0	1	4	2,1	11,5

DHX15_HUMAN	25,4	162,4	1	4	4,4	9,4
SKP1_HUMAN	25,4		1		11,7	
1433E_HUMAN	25,2		1		11,4	
SERA_HUMAN	24,5		1		1,5	
DAZP1_HUMAN	24,2	49,6	1	1	3,9	3,9
TIF1B_HUMAN	24,1		1		2,4	
SNF5_HUMAN	24,0		1		8,1	
TRI47_HUMAN	23,5		1		4,7	
SMU1_HUMAN	23,3		1		3,3	
GANAB_HUMAN	23,0		1		2,4	
DCTP1_HUMAN	23,0		1		11,8	
ATPA_HUMAN	22,9	33,2	1	1	4,0	4,0
PAN3_HUMAN	22,7		1		4,3	
TTK_HUMAN	22,2	17,1	1	1	3,6	3,6
ATX2L_HUMAN	20,0	35,9	1	1	2,1	2,1
ATR_HUMAN	19,8		1		0,5	
RS7_HUMAN	19,7		1		5,7	
HDHD5_HUMAN	19,3		1		6,4	
XPO5_HUMAN	19,1		1		2,8	
NDUA4_HUMAN	19,0		1		14,8	
IMA7_HUMAN	18,6		1		7,6	
PDD2L_HUMAN	18,6	29,8	1	1	4,2	4,2
RL23A_HUMAN	18,2		1		8,3	
SSRD_HUMAN	18,0		1		6,4	
IPYR2_HUMAN	17,9		1		4,8	
IF4E2_HUMAN	17,9	92,4	1	2	11,4	16,7
GLOD4_HUMAN	16,8		1		4,2	
AIFM1_HUMAN	16,7		1		3,8	
EIF3D_HUMAN	16,7	25,0	1	1	3,6	3,6
HNRPD_HUMAN	16,7		1		2,3	
KBL_HUMAN	16,2	20,9	1	1	4,3	10,5
HV366_HUMAN	16,1	32,3	1	1	19,8	19,8
NEK9_HUMAN	16,0		1		1,7	
DDX50_HUMAN	16,0		1		1,6	
ANM1_HUMAN	15,9		1		3,9	
HV323_HUMAN	15,6		1		19,7	
EYA1_HUMAN	15,4		1		3,2	
SMCA2_HUMAN	15,3		1		0,6	
HV353_HUMAN	15,3	25,6	1	1	19,8	19,8
TBR1_HUMAN	15,0		1		1,8	
XIRP1_HUMAN	14,8		1		0,8	
TCPG_HUMAN	14,8		1		4,8	
MAZ_HUMAN	14,7		1		10,7	
AL1L2_HUMAN	14,1	34,1	1	1	2,3	3,1
AAMP_HUMAN	14,1		1		13,6	
SF3B2_HUMAN	14,1		1		2,6	
USP9X_HUMAN	14,0		1		1,5	
RL27A_HUMAN	13,7		1		14,9	

Supplementary table S8: Raw mass spectrometry data for GFP-ZMYM4 experiment 3

protein	Scores A GFP-ZMYM4	Scores B GFP	Peptides A	Peptides B	SC (A) [%]	SC (B) [%]
ZMYM4_HUMAN	6526,7		84		59,1	
TBB5_HUMAN	2315,4	2279,9	28	27	76,4	76,4
TBB4B_HUMAN	2102,1	2022,0	23	23	73,5	61,6
TBB4A_HUMAN	2022,8	1968,7	22	22	61,3	57,4
HSP7C_HUMAN	1713,5	895,1	23	16	52,2	41,5
TBB2B_HUMAN	1565,5		20		55,3	
HS71A_HUMAN	1558,4	1245,2	20	19	49,8	45,6
IRS4_HUMAN	1533,8	1123,9	22	19	36,0	34,8
TBA1B_HUMAN	1430,5	1232,0	16	15	54,3	52,5
UBQL4_HUMAN	1235,0		16		69,7	
TBA1C_HUMAN	1213,7	1006,9	14	13	51,2	49,4
TNR6B_HUMAN	1186,3	776,1	21	16	28,8	23,9
DJC10_HUMAN	1183,7	698,5	12	10	30,5	26,4
MAGD1_HUMAN	1163,4	1323,0	11	14	30,6	31,6
C1QBP_HUMAN	1150,7	940,6	12	11	62,8	62,8
PYR1_HUMAN	1046,3	366,6	18	10	17,7	8,9
TBB3_HUMAN	1040,6		16		29,3	
TNR6A_HUMAN	1035,6	1074,1	20	18	25,3	22,4
CH60_HUMAN	923,8	825,5	13	15	38,7	46,1
ACTB_HUMAN	869,4	862,4	9	10	38,7	38,7
HELZ_HUMAN	867,4	808,3	17	18	15,4	16,7
GRP78_HUMAN	845,8	524,6	14	9	36,9	26,1
TBB6_HUMAN	796,9	667,9	11	11	28,5	28,7
UBP54_HUMAN	791,2	1281,5	14	23	16,3	25,1
TRI32_HUMAN	789,2	183,0	13	4	36,9	11,6
UBQL1_HUMAN	774,2		12		51,1	
PRR12_HUMAN	740,9		11		20,8	
DNJA3_HUMAN	707,0	608,4	13	8	35,0	20,6
HNRPM_HUMAN	704,8	763,1	13	12	28,8	26,4
ATPB_HUMAN	681,8	1004,7	11	16	39,5	50,5
UBP2L_HUMAN	681,5	750,4	10	12	23,4	26,5
HDAC6_HUMAN	656,1	136,2	9	3	20,7	6,5
HNRPU_HUMAN	651,6	639,8	9	9	22,4	22,1
FUBP2_HUMAN	651,3	446,3	8	7	21,5	18,7
DYL1_HUMAN	639,6	50,9	5	1	37,1	24,7
ROA2_HUMAN	621,7	791,2	7	11	41,4	49,0
ANKH1_HUMAN	603,7	834,7	11	17	10,4	13,4
GRP75_HUMAN	593,1	190,3	11	5	31,5	16,6
DNJA1_HUMAN	590,8	458,3	9	9	35,8	41,6
HNRH1_HUMAN	582,2	505,6	7	5	24,7	19,4
RU2A_HUMAN	566,7	51,4	7	2	40,8	19,2
AKP8L_HUMAN	564,3	363,5	8	7	25,5	23,4
ZMYM2_HUMAN	544,2		14		20,9	
PRKDC_HUMAN	538,4	241,4	13	9	5,6	3,8
DDX3X_HUMAN	536,5	570,1	10	10	23,0	23,0
MATR3_HUMAN	534,8	390,1	10	7	22,6	14,9
DHX9_HUMAN	524,1	678,1	13	17	21,6	25,6
VIME_HUMAN	514,2	1003,1	9	16	30,0	37,1
K0355_HUMAN	497,3	547,9	13	13	25,4	26,5
RAVR1_HUMAN	495,0	551,6	7	9	27,4	33,5
XPO2_HUMAN	489,9	222,6	10	4	20,6	8,3
PIMT_HUMAN	479,8	319,1	7	6	51,1	44,9
EF1A1_HUMAN	466,6	489,3	5	5	30,5	30,5
SDCG3_HUMAN	453,2	559,2	8	7	28,7	30,8
ROA1_HUMAN	453,0	467,2	6	6	37,4	37,1
K2C1_HUMAN	444,2	1159,4	9	14	16,3	28,0
H2B1J_HUMAN	443,6	528,6	5	6	26,2	26,2
SC16A_HUMAN	430,9	342,3	6	6	8,3	8,1
KDM1A_HUMAN	430,0		10		28,4	
CLH1_HUMAN	414,9	240,1	10	6	13,2	6,9
NONO_HUMAN	413,0	946,1	6	8	30,4	33,8
DDX5_HUMAN	392,6	461,3	7	7	17,3	17,6
ILF2_HUMAN	389,2	404,7	5	5	32,1	32,1
AMRA1_HUMAN	384,1	349,8	8	7	15,9	15,1

RBM14_HUMAN	372,3	585,3	7	9	35,1	36,5
HNRPF_HUMAN	367,7	501,5	4	6	22,2	29,2
USP9X_HUMAN	365,3	81,7	9	1	7,8	1,1
HNRPK_HUMAN	346,0	459,5	6	7	35,6	30,5
AKAP8_HUMAN	342,7	329,4	5	5	23,0	23,0
AGO2_HUMAN	337,6	239,5	6	5	9,8	8,1
NUCL_HUMAN	336,3	354,0	7	7	15,1	15,1
RS3_HUMAN	335,6	297,3	7	6	34,6	35,8
H2A2C_HUMAN	335,1	344,2	4	4	44,2	44,2
DNJA2_HUMAN	327,7	247,9	8	5	32,0	27,7
DCAF7_HUMAN	327,2	411,7	4	4	28,1	28,1
PTBP1_HUMAN	316,3	332,8	4	7	20,2	33,0
H2A1H_HUMAN	304,2	320,2	3	3	44,5	44,5
H4_HUMAN	302,5	357,8	6	7	31,1	32,0
HNRH2_HUMAN	299,7	318,2	4	4	19,2	19,2
ROA3_HUMAN	298,1	315,4	3	4	12,4	28,6
FAS_HUMAN	297,8	170,1	6	4	4,1	2,7
KPYM_HUMAN	297,7	135,4	6	3	21,1	10,7
HS90B_HUMAN	296,4	270,3	6	6	14,8	15,7
AT1A1_HUMAN	285,4	140,9	5	4	9,5	8,2
PABP1_HUMAN	281,1	630,3	6	11	19,0	29,2
IF2B1_HUMAN	278,5	381,5	3	5	8,7	14,4
K1C10_HUMAN	278,4	1215,3	5	16	19,0	27,6
DDX17_HUMAN	277,2	400,4	7	8	12,1	13,9
K1C9_HUMAN	274,1	886,2	5	9	32,6	48,8
YYAP1_HUMAN	267,8		5		12,6	
DNJB6_HUMAN	263,5	280,7	4	4	22,4	22,4
HNRPL_HUMAN	258,7	384,6	5	8	18,0	23,4
RCN2_HUMAN	255,3	206,0	4	4	25,2	23,3
IMB1_HUMAN	255,1	154,4	6	5	17,5	15,5
LANC1_HUMAN	253,5	221,5	6	4	29,1	20,6
HS90A_HUMAN	249,3	227,4	5	6	12,8	18,4
RL4_HUMAN	246,0	265,9	5	7	18,0	22,7
SFPQ_HUMAN	244,9	458,2	4	6	10,2	16,8
TCPE_HUMAN	243,1	139,7	4	3	17,0	13,9
PABP4_HUMAN	242,2	524,4	4	7	10,4	17,7
CALX_HUMAN	241,9	121,5	5	3	18,4	11,7
RS15_HUMAN	239,5	197,6	3	2	28,3	28,3
XRCC6_HUMAN	236,2	289,4	5	6	14,4	16,3
IF4A1_HUMAN	233,0	160,1	6	5	31,5	25,6
VWA8_HUMAN	232,4	51,5	6	2	5,6	2,0
BCOR_HUMAN	220,8	135,2	6	4	7,4	5,0
ZMYM3_HUMAN	210,1		5		9,2	
THIL_HUMAN	209,0	238,0	3	4	17,1	21,5
DHB4_HUMAN	201,3	211,9	5	5	15,2	15,2
IRAK1_HUMAN	199,8	138,8	4	2	11,7	7,2
PPIA_HUMAN	198,8	162,3	4	3	41,2	35,8
LARP1_HUMAN	194,5	264,8	3	7	6,8	14,3
ILF3_HUMAN	194,0	357,1	3	7	9,3	17,3
ATPA_HUMAN	192,9	472,2	4	8	14,3	30,6
PEF1_HUMAN	192,3	157,4	3	2	17,3	13,4
RS8_HUMAN	191,5	161,3	4	4	26,9	26,9
1433Z_HUMAN	190,7	201,8	4	4	19,2	19,2
ACTH_HUMAN	189,0	195,6	3	4	10,6	10,6
TFG_HUMAN	188,2	26,8	4	1	18,5	6,0
FBX22_HUMAN	185,9		5		33,3	
1433E_HUMAN	182,2	237,8	4	3	23,1	11,4
PARP1_HUMAN	179,5	263,1	3	6	7,9	13,6
RBM39_HUMAN	179,5	214,1	4	5	12,3	15,1
AT2A2_HUMAN	177,0	81,5	2	2	4,2	4,2
ARF3_HUMAN	176,9	108,3	3	2	22,1	16,0
RS5_HUMAN	176,5	169,9	3	3	23,5	23,5
WDCP_HUMAN	175,9	98,9	2	1	7,9	4,0
PRP8_HUMAN	175,7	171,1	5	3	4,4	2,8
RS3A_HUMAN	175,2	162,7	3	2	16,7	11,7
NDUS3_HUMAN	174,0	177,0	4	4	19,7	21,2
AGO3_HUMAN	173,8		4		8,0	
RLA0_HUMAN	171,7	85,0	3	2	22,1	16,7

LAP2B_HUMAN	171,3	232,4	3	6	15,4	27,1
DJB11_HUMAN	169,2	101,5	4	2	18,2	4,7
U520_HUMAN	167,8	108,6	5	3	4,2	2,4
IF5A1_HUMAN	167,2	248,0	2	3	30,5	31,2
UBR5_HUMAN	165,9	113,5	5	4	3,6	3,8
PUR4_HUMAN	163,0	60,2	3	1	5,6	2,0
H2AV_HUMAN	162,7	188,2	2	2	29,7	29,7
EF2_HUMAN	161,5	110,7	5	4	11,2	8,5
ATPG_HUMAN	161,3	166,6	3	3	18,8	18,8
DYHC1_HUMAN	160,6		6		3,1	
RS2_HUMAN	159,4	122,0	3	2	16,0	8,2
NNRE_HUMAN	156,5	48,7	4	1	37,8	6,6
SALL2_HUMAN	155,8		2		5,4	
YBOX1_HUMAN	155,3	105,6	2	2	14,5	14,5
DYL2_HUMAN	153,1		2		37,1	
NPM_HUMAN	152,3	328,5	3	5	27,9	41,5
COPE_HUMAN	152,3	48,0	3	1	18,5	8,4
RS4X_HUMAN	151,7	187,0	3	3	11,0	16,7
MRP_HUMAN	150,8	213,8	2	2	36,9	36,9
HSPB1_HUMAN	148,5	177,5	3	4	28,3	46,8
DDB1_HUMAN	148,3	188,9	4	3	10,5	7,7
SEC13_HUMAN	148,1	102,0	2	2	12,4	12,4
TCPA_HUMAN	147,1	86,1	2	1	11,0	6,1
PUM1_HUMAN	146,8	116,8	3	3	5,2	5,2
RL23_HUMAN	146,5	238,2	2	3	25,0	27,1
SAFB1_HUMAN	144,4	271,5	3	5	10,9	12,6
HNRPC_HUMAN	143,1	283,3	2	6	8,8	16,7
FA83H_HUMAN	136,4		4		8,3	
GANAB_HUMAN	133,4	54,6	3	1	5,8	2,4
SKP1_HUMAN	132,9	95,5	2	2	28,8	28,8
SC23A_HUMAN	132,8		3		10,2	
VDAC1_HUMAN	131,8	235,0	3	5	14,8	25,8
ROA0_HUMAN	131,2	112,6	1	1	5,2	5,2
DHB12_HUMAN	130,6		2		14,1	
HNRL1_HUMAN	130,1	155,2	4	4	10,4	10,4
EF1G_HUMAN	130,1	135,6	3	3	13,7	16,7
TRAP1_HUMAN	128,7		2		6,1	
RAN_HUMAN	127,5	189,9	2	4	29,6	29,6
RSSA_HUMAN	126,6	158,8	2	3	14,9	39,0
XRN2_HUMAN	125,9	401,8	3	10	6,3	20,3
HAX1_HUMAN	125,7	148,7	3	3	29,0	29,0
RUVB1_HUMAN	124,3	84,6	3	2	12,3	7,0
TCPG_HUMAN	124,0	34,3	5	1	18,3	5,1
RCOR1_HUMAN	122,7		3		10,3	
RLA1_HUMAN	121,9	183,1	2	4	51,8	66,7
TADBP_HUMAN	121,7	190,9	3	4	17,4	28,7
AR6P1_HUMAN	120,9	67,9	1	1	13,8	13,8
TCPZ_HUMAN	120,8	53,9	4	2	13,2	5,3
RS17_HUMAN	120,6	124,9	2	2	32,6	32,6
RB11A_HUMAN	118,4	66,0	1	1	13,4	13,4
RS23_HUMAN	117,7	166,7	2	3	26,6	27,3
PHB2_HUMAN	117,2	229,5	3	5	13,7	26,1
U5S1_HUMAN	116,8	100,0	3	3	6,4	6,6
RS24_HUMAN	116,4	141,8	2	2	20,3	20,3
AIMP2_HUMAN	114,6		1		11,3	
CNOT1_HUMAN	114,4	16,4	3	1	1,3	0,4
RU17_HUMAN	114,1	101,8	2	2	14,2	14,2
TAGL2_HUMAN	112,7	64,4	2	1	18,6	9,0
SFXN1_HUMAN	112,2	144,7	1	2	10,6	15,2
XPO1_HUMAN	111,5	85,2	1	1	1,4	1,4
MDHM_HUMAN	110,6	32,7	2	1	8,3	3,3
QCR2_HUMAN	106,5	160,3	4	4	27,4	26,9
PDIA6_HUMAN	106,0	21,0	3	1	12,7	5,5
RL7A_HUMAN	105,6	18,9	2	1	13,5	7,5
YTHD2_HUMAN	104,5	70,2	2	2	8,5	8,5
ZSWM3_HUMAN	103,0		4		9,9	
NOP58_HUMAN	102,7	179,8	2	2	7,0	7,0
BCKD_HUMAN	102,4		2		9,2	

RL10_HUMAN	102,0	145,9	2	2	19,2	19,2
PHB_HUMAN	101,3	227,7	2	4	11,4	22,8
RBP56_HUMAN	101,0	110,4	1	1	5,7	5,7
ROAA_HUMAN	100,5	90,5	3	2	27,7	25,3
EMD_HUMAN	99,4	86,6	2	3	14,2	25,6
RL37A_HUMAN	99,3	42,9	1	1	19,6	19,6
MCM5_HUMAN	98,5	53,6	1	1	4,4	4,4
AGO1_HUMAN	98,4	17,3	3	1	4,9	1,9
ESYT1_HUMAN	97,9		3		6,0	
MBB1A_HUMAN	97,8	136,0	3	5	5,0	7,9
EIF3L_HUMAN	97,2		1		3,5	
KCRB_HUMAN	96,7	287,2	2	5	8,7	19,7
HNRDL_HUMAN	96,7	203,1	3	3	13,1	13,1
NU107_HUMAN	95,9	125,4	2	3	4,1	6,3
4ET_HUMAN	95,6	214,8	3	3	7,7	8,1
MIIP_HUMAN	95,4	112,2	1	1	8,8	8,8
EIF3A_HUMAN	94,3		3		3,5	
ECHA_HUMAN	93,2	169,3	4	4	9,2	9,2
AHR_HUMAN	93,0	94,5	3	2	11,0	7,5
P5CS_HUMAN	92,7	79,0	1	2	3,0	5,9
PGRC1_HUMAN	92,5	83,0	2	2	20,5	20,5
RLA2_HUMAN	92,5	193,1	1	4	28,7	53,9
SPTC1_HUMAN	92,3		2		7,8	
GGYF2_HUMAN	92,0	181,6	2	4	2,9	6,5
LRC59_HUMAN	91,8	60,7	3	1	18,2	8,1
COPA_HUMAN	91,8		3		5,8	
RPB2_HUMAN	89,6	58,2	1	1	3,3	3,3
ADT2_HUMAN	89,2	185,7	3	5	11,1	20,1
FBRL_HUMAN	88,9	147,3	2	4	12,1	21,8
COX2_HUMAN	88,8	89,7	1	1	7,0	7,0
SYFA_HUMAN	88,5	38,1	2	1	5,9	3,1
ILVBL_HUMAN	88,2		3		11,9	
TOP2A_HUMAN	87,8	348,7	3	9	3,1	11,1
TRIM5_HUMAN	87,6		1		3,4	
ALG13_HUMAN	86,9	32,6	2	1	3,0	1,6
ELAV1_HUMAN	86,7	177,5	3	4	15,6	21,2
GASP2_HUMAN	86,1	55,1	2	1	4,3	2,1
PYRG1_HUMAN	85,7	95,4	2	2	9,0	9,0
PUR6_HUMAN	85,2		2		11,8	
PRP19_HUMAN	84,3	72,8	2	2	14,1	14,1
PRC2B_HUMAN	83,8	41,8	3	1	2,6	1,1
RL17_HUMAN	83,8	40,5	2	1	18,5	8,7
VDAC2_HUMAN	83,1	412,1	2	7	14,3	40,1
PP1G_HUMAN	83,1		1		5,3	
RUXE_HUMAN	82,9	75,7	1	2	27,2	39,1
RS30_HUMAN	82,6	66,4	1	1	16,9	16,9
HSP74_HUMAN	81,5		3		8,2	
PARK7_HUMAN	81,4	92,3	2	2	23,8	23,8
CBSL_HUMAN	81,0	38,8	2	1	11,3	7,4
SDHA_HUMAN	80,9	214,4	3	5	9,0	17,6
RL18_HUMAN	80,6	80,6	1	1	6,9	6,9
RUVB2_HUMAN	80,5	31,6	3	1	12,7	6,9
HNRPQ_HUMAN	80,3	205,4	3	6	10,4	17,2
CS068_HUMAN	80,2		2		8,0	
TIM50_HUMAN	80,2	118,0	3	3	12,5	12,5
RS16_HUMAN	79,7	107,1	2	2	14,4	14,4
NOL6_HUMAN	79,7	58,0	1	1	2,7	2,7
H2AY_HUMAN	79,7	265,5	1	5	5,9	23,4
SYTM_HUMAN	79,2	74,2	2	1	5,8	2,5
RAB1C_HUMAN	78,5	47,6	1	1	8,0	8,0
RAB10_HUMAN	78,5	21,6	2	1	17,5	12,0
MSI1H_HUMAN	78,0	125,8	2	3	13,5	18,2
RL1D1_HUMAN	77,7	143,1	2	4	5,1	12,0
FAF2_HUMAN	77,3	81,9	2	2	10,6	10,6
OST48_HUMAN	77,1	83,9	1	1	6,4	6,4
NDUS2_HUMAN	77,1	128,4	1	3	5,0	13,6
EIF3F_HUMAN	76,4		1		5,3	
KCRU_HUMAN	76,1	30,0	2	1	11,3	6,2

RL19_HUMAN	75,4	32,2	1	1	8,7	8,7
MIF_HUMAN	72,8	54,4	1	1	18,3	18,3
MCM7_HUMAN	72,8	57,9	2	1	5,8	3,6
TS101_HUMAN	72,5	83,7	2	3	11,8	16,2
RS27_HUMAN	72,1	82,5	2	2	16,7	15,5
HNRPD_HUMAN	72,0	114,8	3	3	11,5	11,5
CMC2_HUMAN	71,5	95,9	2	2	8,7	8,7
SPB1_HUMAN	71,2	183,0	2	4	4,7	9,1
SERPH_HUMAN	70,3	48,0	1	1	6,2	6,2
PRC2A_HUMAN	70,1	477,6	3	12	2,4	13,3
SYIC_HUMAN	70,1	71,3	2	1	4,6	2,6
LDHA_HUMAN	70,0	69,6	1	1	4,5	4,5
PCBP1_HUMAN	69,7	105,7	3	2	20,2	11,5
PTN13_HUMAN	69,2	22,4	2	1	2,0	1,0
HNRH3_HUMAN	69,1	74,1	1	1	4,9	4,9
SDF2L_HUMAN	69,1	69,9	1	1	10,9	10,9
DHX30_HUMAN	69,1	51,5	2	1	2,5	1,5
MARCS_HUMAN	69,0	88,8	1	2	9,6	24,7
STML2_HUMAN	68,8	252,4	2	5	12,6	28,1
RL11_HUMAN	68,5	50,9	1	1	7,9	7,9
DHX15_HUMAN	68,3		2		5,9	
RL9_HUMAN	68,2	89,6	1	1	14,1	14,1
SSRD_HUMAN	68,1	61,8	2	1	17,3	6,4
IF4A3_HUMAN	67,8	70,5	2	2	10,7	10,7
LDHB_HUMAN	67,5	83,6	1	2	4,5	5,1
HS105_HUMAN	67,4		2		3,0	
FUS_HUMAN	67,3	183,0	2	3	9,3	12,4
PAN3_HUMAN	67,1	67,3	1	1	4,3	4,3
MSI2H_HUMAN	66,7	63,8	2	2	9,5	9,5
CN166_HUMAN	66,7	24,6	1	1	5,7	6,6
RAF1_HUMAN	66,3	15,2	2	1	7,7	4,2
MIC13_HUMAN	66,3	62,3	1	1	16,9	16,9
COF1_HUMAN	65,8	64,0	2	1	27,1	16,9
RL40_HUMAN	65,5	74,4	1	1	12,5	12,5
PSB1_HUMAN	65,5	48,5	1	1	11,2	11,2
RL3_HUMAN	65,3	124,8	1	3	6,0	11,9
CRKL_HUMAN	65,2	183,6	2	4	13,9	25,1
RS10_HUMAN	64,9	46,8	1	1	5,5	5,5
K22E_HUMAN	64,6	1067,5	2	15	6,6	38,7
PABP2_HUMAN	64,5	76,9	1	1	14,7	14,7
ECM29_HUMAN	64,3		2		2,2	
RS13_HUMAN	62,8	87,5	2	3	18,5	18,5
CRTAP_HUMAN	62,1	41,4	1	1	6,2	6,2
RL6_HUMAN	62,0	25,8	2	1	11,1	5,9
EWS_HUMAN	61,9	204,8	1	3	7,9	11,6
VPS28_HUMAN	61,7	37,4	2	1	13,6	6,8
RS18_HUMAN	61,0	36,3	2	1	11,2	5,3
SMCA5_HUMAN	60,9	102,4	1	2	1,4	3,4
TNPO1_HUMAN	60,9		2		5,1	
GBB2_HUMAN	60,7	100,1	1	2	6,8	12,6
RL31_HUMAN	60,5	94,6	1	2	11,2	18,4
KLHL9_HUMAN	59,6		2		4,9	
VDAC3_HUMAN	59,5	197,6	1	4	3,5	21,9
GTF2I_HUMAN	59,2	63,2	2	2	5,0	5,0
RTKN_HUMAN	58,8	32,3	1	1	4,4	4,4
FA83B_HUMAN	58,7	19,7	2	1	4,4	2,2
HDAC2_HUMAN	58,6	56,5	1	3	3,7	10,0
RL24_HUMAN	58,3	82,2	1	2	8,3	14,0
CALM1_HUMAN	58,2		1		24,8	
MCCB_HUMAN	57,3	71,0	2	2	9,8	5,5
RAB7A_HUMAN	57,3	43,6	1	1	6,8	6,8
DDX54_HUMAN	57,3	60,6	1	1	3,6	3,6
RS28_HUMAN	57,2	71,1	1	1	17,4	17,4
PAIRB_HUMAN	56,6		1		3,9	
ADT3_HUMAN	56,5	148,4	2	3	7,4	11,4
RMXL1_HUMAN	56,3	65,5	1	1	3,6	3,6
1433B_HUMAN	56,1	49,9	2	2	11,8	11,8
ATP5H_HUMAN	55,9	94,2	2	2	23,6	23,6

DDX21_HUMAN	55,8	83,8	2	3	5,0	8,0
BORG5_HUMAN	55,8	118,7	1	3	4,9	18,4
RS19_HUMAN	55,7		1		9,0	
BASI_HUMAN	55,4	44,4	1	1	4,7	4,7
DDX1_HUMAN	55,4	54,1	1	1	2,8	2,8
ZSWM1_HUMAN	55,3		2		8,7	
AP1G1_HUMAN	55,2		1		3,3	
RL15_HUMAN	54,9	44,0	1	1	6,9	6,9
RHOA_HUMAN	54,7	98,2	2	2	21,2	21,2
SERA_HUMAN	54,2	43,5	2	1	5,1	1,5
NU205_HUMAN	54,0	80,3	1	2	1,1	2,2
RM12_HUMAN	53,6	105,4	1	2	12,1	29,3
AAAS_HUMAN	53,0	48,5	1	1	4,2	4,2
SHKB1_HUMAN	52,6		2		5,0	
XRCC5_HUMAN	52,4	138,5	1	3	3,0	9,6
WDR43_HUMAN	52,2	68,3	1	1	4,3	4,3
ZN281_HUMAN	51,7	47,9	2	2	5,3	3,9
SSRP1_HUMAN	51,5	341,6	1	8	1,6	19,6
EF1B_HUMAN	51,5	52,8	1	1	6,7	6,7
ACLY_HUMAN	51,5	33,7	1	1	1,5	1,5
PYGL_HUMAN	51,4	24,3	1	1	5,3	5,3
RL35_HUMAN	51,2	46,3	1	1	10,6	10,6
CNO11_HUMAN	51,0		2		8,6	
SP16H_HUMAN	50,6	263,2	1	6	2,5	10,2
FBSL_HUMAN	50,3	68,6	1	2	1,1	3,4
UBA1_HUMAN	49,5		2		3,6	
SF3B6_HUMAN	49,4		1		9,6	
NOP56_HUMAN	49,2	235,4	2	6	7,4	21,2
RS12_HUMAN	49,1		2		17,4	
RFOX2_HUMAN	49,0	88,2	1	2	3,8	10,5
RM04_HUMAN	48,8	58,0	1	1	6,1	6,1
RL30_HUMAN	48,4	54,1	1	1	13,9	13,9
PSMD6_HUMAN	48,1		1		6,2	
NU155_HUMAN	48,0	105,6	2	2	3,5	3,5
MD2L1_HUMAN	47,2		1		10,2	
RS26_HUMAN	46,8	43,0	1	1	13,0	13,0
KCTD3_HUMAN	46,7		2		4,8	
WDR36_HUMAN	46,4	91,8	1	1	2,3	2,3
RM37_HUMAN	46,3	41,6	2	1	9,0	5,2
S61A2_HUMAN	46,2		1		5,3	
ACSL3_HUMAN	46,2		1		2,4	
WDR6_HUMAN	46,0	42,2	1	1	1,2	1,2
WDR59_HUMAN	45,9		2		5,4	
CNOT3_HUMAN	45,8	16,3	2	1	13,4	4,6
ELOC_HUMAN	45,7	36,5	1	1	26,8	26,8
IPO5_HUMAN	45,6	20,4	2	1	4,6	2,8
MYCB2_HUMAN	45,4		1		0,6	
AAAT_HUMAN	45,4	45,1	1	1	2,0	2,0
IASPP_HUMAN	45,1	101,9	1	3	2,9	7,5
PCBP2_HUMAN	45,1	128,2	2	3	9,0	17,5
DHE3_HUMAN	45,1	13,6	1	1	3,4	3,8
RAB5C_HUMAN	44,8		1		11,1	
ACADM_HUMAN	44,7	27,3	1	1	7,4	7,4
PPP6_HUMAN	44,7		1		7,2	
IMA1_HUMAN	44,6	90,9	1	3	6,2	15,7
CAPZB_HUMAN	44,4		1		9,0	
FKBP8_HUMAN	44,3	72,7	1	1	7,0	7,0
CNOT2_HUMAN	44,1	51,9	1	1	7,0	7,0
SURF4_HUMAN	44,0	38,3	1	1	6,7	6,7
RPN1_HUMAN	43,9	14,2	1	1	2,0	2,5
FXRD2_HUMAN	43,8		1		3,8	
EFTU_HUMAN	43,8	41,8	1	1	3,1	3,1
AMPB_HUMAN	43,7		1		2,9	
NUP93_HUMAN	43,2	31,5	2	1	4,4	1,2
ASNA_HUMAN	43,1		1		9,8	
IQEC1_HUMAN	43,0		1		2,3	
UCRI_HUMAN	42,6	64,7	1	2	8,8	16,4
GFPT2_HUMAN	42,5	50,6	1	1	3,2	3,2

SF01_HUMAN	42,3	81,1	1	2	3,3	5,3
EIF3E_HUMAN	41,7		1		4,0	
PSDE_HUMAN	41,5		2		18,1	
NIP7_HUMAN	41,4	61,4	1	1	15,6	15,6
SSRA_HUMAN	41,3		1		5,2	
SIAH1_HUMAN	41,1		1		7,8	
FANCI_HUMAN	41,0	42,1	1	1	2,5	2,5
NDUA4_HUMAN	41,0	42,9	1	1	14,8	14,8
CUL3_HUMAN	40,9		1		3,3	
SPD2A_HUMAN	40,9	26,6	2	1	1,9	1,9
YTHD1_HUMAN	40,8	24,1	1	1	6,4	6,4
ECHB_HUMAN	40,3	18,6	1	1	3,0	3,0
BZW1_HUMAN	40,2		2		12,6	
AQR_HUMAN	40,1		1		2,2	
RPAB5_HUMAN	40,0	72,1	1	1	35,8	35,8
ODPB_HUMAN	39,8	41,5	1	2	7,5	12,0
RL22_HUMAN	39,5	182,7	1	3	20,3	40,6
SAP_HUMAN	39,0		1		5,5	
NH2L1_HUMAN	39,0	32,0	1	1	21,9	21,9
TCPQ_HUMAN	38,3		1		2,9	
MCM3_HUMAN	38,3	80,9	1	2	1,7	4,2
CBX3_HUMAN	37,6	125,3	1	2	8,7	24,6
IF4E2_HUMAN	37,4	51,0	1	1	11,4	11,4
THOC4_HUMAN	37,1	90,0	1	2	7,0	17,1
RS9_HUMAN	37,0	24,4	1	1	5,7	5,7
H32_HUMAN	37,0	102,9	1	1	23,5	23,5
PROF1_HUMAN	37,0	31,5	1	1	11,4	11,4
2AAA_HUMAN	37,0	30,6	1	1	3,1	3,1
CPSF6_HUMAN	36,9	33,2	1	1	4,5	4,5
XPO5_HUMAN	36,8		1		2,8	
NP1L1_HUMAN	36,7	27,1	2	1	7,2	4,3
AUP1_HUMAN	36,7		1		5,3	
MARC1_HUMAN	36,7	52,3	1	1	7,4	7,4
MCMBP_HUMAN	36,6		1		3,9	
SMU1_HUMAN	36,5	40,6	1	1	3,3	3,3
ASPH_HUMAN	36,3		1		2,4	
SC24C_HUMAN	36,2		1		3,0	
NUBP2_HUMAN	36,1	58,0	1	1	9,6	9,6
SRS10_HUMAN	36,0	61,9	1	1	6,1	6,1
NAT10_HUMAN	36,0	225,5	2	6	5,1	11,4
PSD12_HUMAN	35,8		1		5,0	
DAZP1_HUMAN	35,7	90,7	1	1	3,9	3,9
SF3A3_HUMAN	35,1	43,0	1	1	4,0	4,0
DECR_HUMAN	35,1		1		6,6	
SMD1_HUMAN	34,7	77,5	1	2	16,8	27,7
MOGS_HUMAN	34,6		1		1,4	
GTPB3_HUMAN	34,3	40,9	1	1	4,7	4,7
GSTP1_HUMAN	34,0	42,0	1	1	9,0	9,0
MDN1_HUMAN	33,7		1		0,5	
TERA_HUMAN	33,0		1		2,1	
DCTP1_HUMAN	32,7		1		11,8	
NOLC1_HUMAN	32,5	49,5	1	1	3,3	3,3
CPSF2_HUMAN	32,4		1		3,1	
WDR75_HUMAN	32,4	87,1	1	2	2,3	5,3
RCC1_HUMAN	32,3	182,1	1	5	6,9	27,3
RBM28_HUMAN	32,3	49,8	1	1	2,9	2,9
SRSF7_HUMAN	32,2	64,0	1	1	8,8	8,8
AL1B1_HUMAN	32,2		1		3,3	
PDCD6_HUMAN	32,1	85,6	1	1	6,8	14,1
LMNB1_HUMAN	32,0	357,8	1	7	2,9	20,6
TKT_HUMAN	31,8		1		2,7	
MGST1_HUMAN	31,7	13,2	1	1	16,1	16,1
FA98A_HUMAN	31,5	29,3	1	1	3,3	2,9
GCN1_HUMAN	31,5		2		1,8	
TCPB_HUMAN	31,4	34,0	1	1	4,1	4,1
FOXG1_HUMAN	31,3	37,9	1	1	4,3	4,3
ANR35_HUMAN	31,0	28,8	1	1	2,0	2,0
UBP7_HUMAN	30,8	52,5	1	1	2,9	2,9

COPB2_HUMAN	30,8		1		2,5	
SUGP2_HUMAN	30,0	37,4	1	1	2,6	2,6
NMD3A_HUMAN	30,0		2		2,0	
NCHL1_HUMAN	29,6		1		0,9	
FXR1_HUMAN	29,5	69,6	1	1	2,3	2,3
SRPRA_HUMAN	29,4		2		8,2	
SFI1_HUMAN	29,3	14,3	1	1	1,4	1,4
SAC1_HUMAN	28,8		1		6,0	
EIF3M_HUMAN	28,4		1		7,2	
NDK8_HUMAN	28,0		1		13,9	
SYEP_HUMAN	27,9		1		1,5	
STAU1_HUMAN	27,6	20,4	1	1	2,3	2,3
POGZ_HUMAN	27,5		1		2,4	
PHS_HUMAN	27,5		1		15,4	
ARL1_HUMAN	27,2	52,3	1	1	8,8	8,8
PIHD1_HUMAN	27,1	27,0	1	1	4,8	4,8
RRP5_HUMAN	27,1	123,8	1	2	2,3	3,4
PDIA1_HUMAN	27,1	102,3	1	2	4,9	10,8
IF6_HUMAN	26,9	63,6	1	1	9,8	9,8
ZN318_HUMAN	26,6	13,3	1	1	1,4	1,4
DESP_HUMAN	26,6		1		0,9	
CATB_HUMAN	26,5	17,2	1	1	5,3	5,3
MMTA2_HUMAN	26,3	69,4	1	2	3,8	9,1
ALBU_HUMAN	26,1		1		2,5	
NU160_HUMAN	25,9	56,7	1	2	1,6	3,3
SAV1_HUMAN	25,8	27,3	1	1	6,0	6,0
TM39B_HUMAN	25,6	16,0	1	1	2,4	2,4
PPOX_HUMAN	25,5		1		6,5	
DNLI4_HUMAN	25,5		1		2,1	
LIGO1_HUMAN	25,4		1		2,1	
RTCB_HUMAN	25,3		1		2,8	
ITPR3_HUMAN	25,1		1		0,5	
ERH_HUMAN	25,0	133,4	1	2	29,8	46,2
CALR_HUMAN	24,8	66,0	1	1	7,0	7,0
FBX21_HUMAN	24,7		1		5,1	
RPF2_HUMAN	24,7	32,3	1	1	2,6	2,6
SNR40_HUMAN	24,7	16,6	1	1	9,5	9,5
CELR3_HUMAN	24,4		1		0,3	
TMM33_HUMAN	24,3		1		4,9	
PSMD1_HUMAN	24,3		1		2,7	
TMEDA_HUMAN	24,3		1		5,0	
PLST_HUMAN	24,3		1		3,5	
HDAC7_HUMAN	24,1		1		0,8	
U2AF2_HUMAN	24,1	64,9	1	2	8,0	11,8
RL7_HUMAN	24,1	44,7	1	2	4,4	11,7
LMNB2_HUMAN	24,0	64,3	1	2	2,7	7,1
TRI65_HUMAN	24,0		1		8,1	
NUP85_HUMAN	24,0	39,1	1	1	2,3	2,3
RAB14_HUMAN	23,9		1		14,0	
GNL3_HUMAN	23,7	39,5	1	1	4,4	4,4
HNRPR_HUMAN	23,6	107,2	1	3	2,1	13,3
CAPR1_HUMAN	23,4	193,5	1	5	5,9	15,4
CRTC3_HUMAN	23,4	160,6	1	4	9,0	19,1
GET4_HUMAN	22,4		1		11,6	
MIA3_HUMAN	22,4		1		1,4	
ACSL1_HUMAN	22,3		1		3,7	
HDHD5_HUMAN	22,1		1		3,1	
CHCH9_HUMAN	22,1		1		18,5	
RHG35_HUMAN	21,8		1		2,1	
SMD3_HUMAN	21,8	21,7	1	1	7,1	7,1
SRPRB_HUMAN	21,8		1		6,3	
DJC16_HUMAN	21,7	13,1	1	1	3,3	3,3
CNOT4_HUMAN	21,6	45,6	1	2	4,5	7,1
RPN2_HUMAN	21,6	100,2	1	3	2,9	10,0
XRN1_HUMAN	21,4		1		1,7	
GHDC_HUMAN	21,3		1		7,2	
RL5_HUMAN	21,3	32,5	1	1	4,4	6,1
HV323_HUMAN	21,1		1		19,7	

ATPO_HUMAN	21,1	53,4	1	1	8,0	10,3
DPOD1_HUMAN	21,0		1		1,0	
NU133_HUMAN	20,8	80,6	1	2	2,9	4,2
CPSF7_HUMAN	20,6	23,8	1	1	5,3	5,3
ZN703_HUMAN	20,4	17,0	1	1	2,0	2,0
TTC7B_HUMAN	20,4	20,7	1	1	2,4	2,4
RS14_HUMAN	20,0	13,8	1	1	13,9	13,9
DX39B_HUMAN	19,8		1		5,1	
ZCCHV_HUMAN	19,8	37,0	1	1	3,1	3,1
KHDR1_HUMAN	19,7	95,0	1	3	4,5	20,1
RD23B_HUMAN	19,7		1		7,6	
COPZ1_HUMAN	19,6		1		18,6	
AL3A2_HUMAN	19,5		1		5,4	
RHG23_HUMAN	19,3	19,5	1	1	0,8	0,8
PPIL3_HUMAN	19,2	13,4	1	1	11,8	11,8
MGN_HUMAN	19,2	36,8	1	1	13,7	13,7
CLPB_HUMAN	19,2		1		3,7	
KITH_HUMAN	19,1		1		15,0	
CX057_HUMAN	19,0		1		2,6	
CDK1_HUMAN	19,0		1		6,7	
GLD2_HUMAN	19,0		1		2,1	
KC1A_HUMAN	18,9	26,8	1	1	3,0	3,0
HV353_HUMAN	18,9		1		19,8	
MXRA7_HUMAN	18,9		1		36,3	
YLPM1_HUMAN	18,8	42,8	1	1	1,5	1,5
CCD47_HUMAN	18,7		1		5,0	
OAT_HUMAN	18,7	37,7	1	1	8,4	8,4
RGSL_HUMAN	18,4		1		1,1	
ATPK_HUMAN	18,4	37,1	1	1	11,7	11,7
HV311_HUMAN	18,3		1		19,7	
PSA5_HUMAN	18,2		1		7,9	
TARA_HUMAN	18,2		1		0,5	
LMAN1_HUMAN	18,2		1		4,1	
PERM_HUMAN	18,1	15,3	1	1	3,9	3,9
IMDH1_HUMAN	18,1		1		6,2	
HDAC1_HUMAN	18,0		1		6,8	
MYH2_HUMAN	17,8	15,6	1	1	0,9	0,9
LAR4B_HUMAN	17,8	126,2	1	4	2,2	11,0
EIF3D_HUMAN	17,8	18,4	1	1	3,6	3,6
MMGT1_HUMAN	17,8	29,8	1	1	18,3	18,3
PPT1_HUMAN	17,5		1		4,9	
RBMS1_HUMAN	17,5	17,8	1	1	9,1	9,1
NT5D2_HUMAN	17,4		1		5,6	
COE3_HUMAN	17,4		1		2,0	
HIP1_HUMAN	17,4		1		1,1	
TOM7_HUMAN	17,4	36,3	1	1	30,9	30,9
MDC1_HUMAN	17,4	289,1	1	6	1,2	8,8
SCO1_HUMAN	17,2	31,0	1	1	9,3	9,3
CLPT1_HUMAN	17,2		1		3,3	
ATD3A_HUMAN	17,1		1		3,3	
GNA13_HUMAN	16,9	17,3	1	1	5,0	5,0
MKKS_HUMAN	16,8		1		1,8	
HV315_HUMAN	16,4	16,5	1	1	19,3	19,3
HLTF_HUMAN	16,1	22,1	1	1	2,8	2,8
M2OM_HUMAN	16,1	129,0	1	2	3,2	8,3
RBM4_HUMAN	15,9	99,4	1	2	7,7	14,6
ALG1_HUMAN	15,8	18,7	1	1	3,4	3,4
GANP_HUMAN	15,8		1		1,9	
TBR1_HUMAN	15,6	16,6	1	1	1,8	1,8
C1TC_HUMAN	15,3		1		4,6	
WDR81_HUMAN	15,2	14,1	2	1	1,3	1,3
CHMP5_HUMAN	15,2	41,6	1	2	22,8	35,2
RAB2A_HUMAN	15,1		1		6,1	
ATP5L_HUMAN	14,8	15,9	1	1	23,3	23,3
HBA_HUMAN	14,7	27,7	1	1	6,3	6,3
NSUN4_HUMAN	14,7		1		2,6	
IF4H_HUMAN	14,7	43,7	1	1	11,7	11,7
WDR12_HUMAN	14,7	23,6	1	1	5,9	5,9

AP3D1_HUMAN	14,5		1		1,6	
BZW2_HUMAN	14,4		1		1,9	
L37A3_HUMAN	13,9		1		0,7	
CGNL1_HUMAN	13,8		1		1,3	
TGM3L_HUMAN	13,8		1		1,7	
SYLC_HUMAN	13,8		1		1,7	
SPEG_HUMAN	13,7		1		0,6	
RM10_HUMAN	13,7	19,2	1	1	7,3	7,3
TIF1B_HUMAN	13,6		1		4,2	
PKHH1_HUMAN	13,6		1		1,1	
PTPRG_HUMAN	13,5		1		0,8	
DHB1_HUMAN	13,4		1		4,0	
PYDC4_HUMAN	13,3		1		31,1	
SPON2_HUMAN	13,2		1		3,6	
PNPT1_HUMAN	13,1		1		2,2	
MSH3_HUMAN	13,1		1		1,8	
RADIL_HUMAN	13,1		1		2,0	

Characterization of the zinc finger proteins ZMYM2 and ZMYM4 as novel B-MYB binding proteins

Hannah Cibis, Abhiruchi Biyanee, Wolfgang Dörner, Henning D. Mootz and Karl-Heinz Klempnauer

Original scans

Fig.1a

Fig.1b

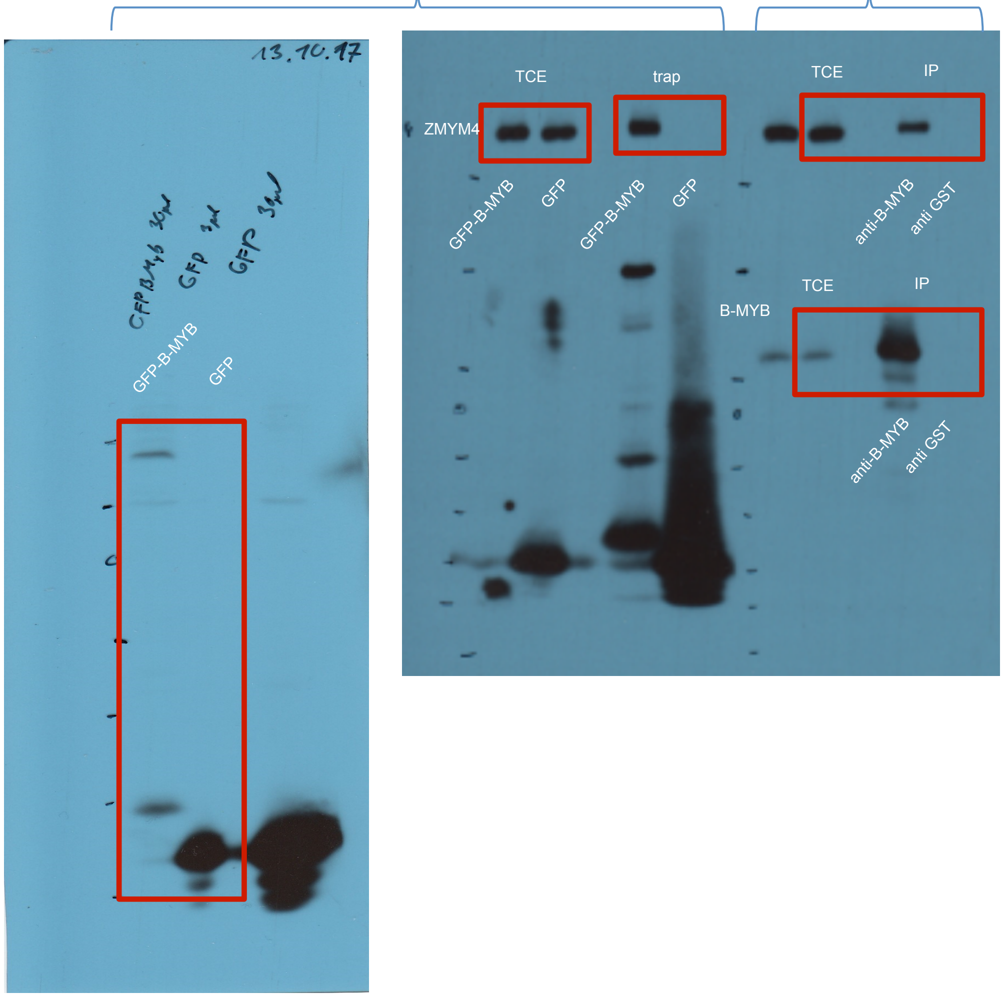

Fig.1c

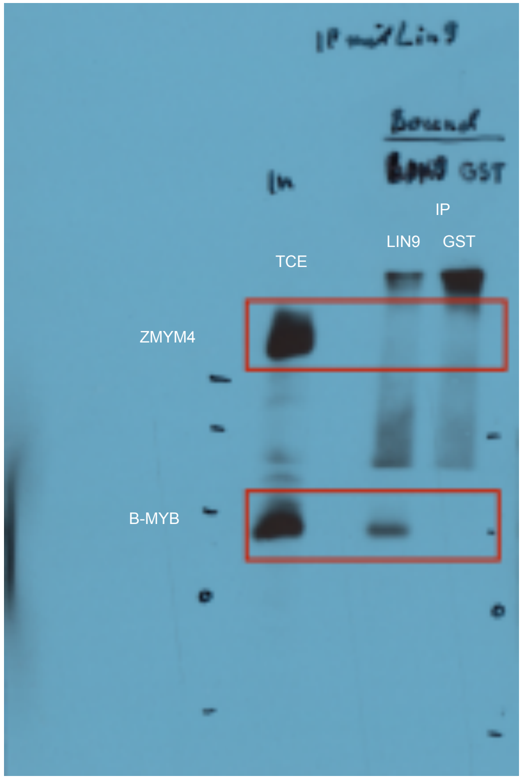

Fig.2b

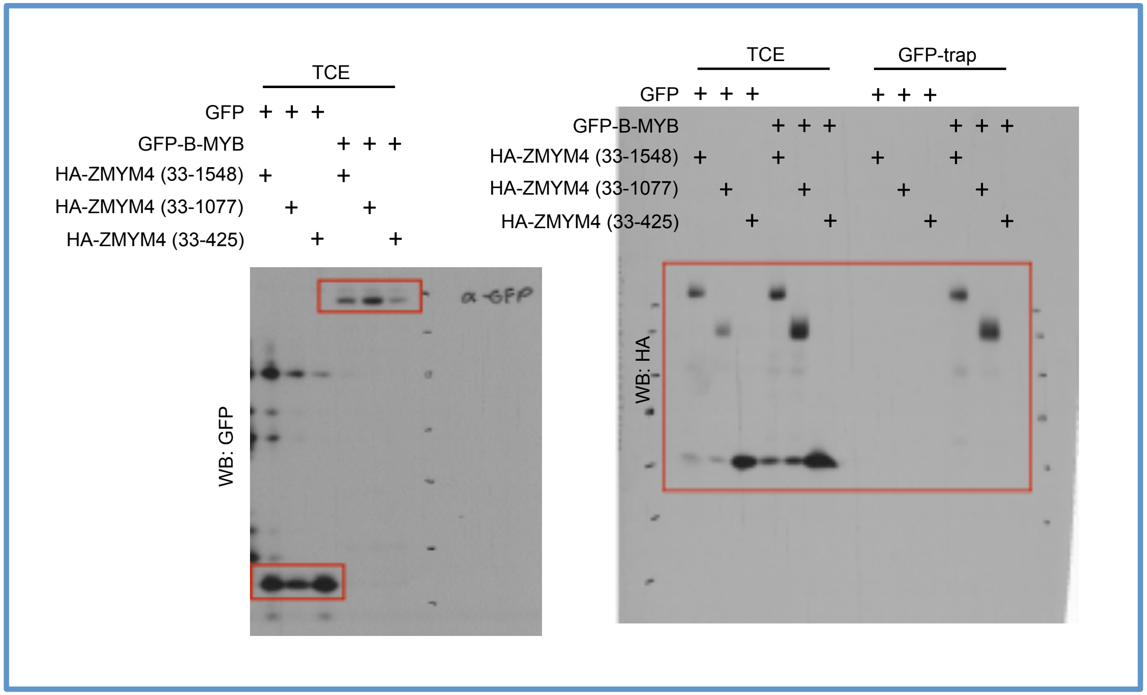

Fig.2c

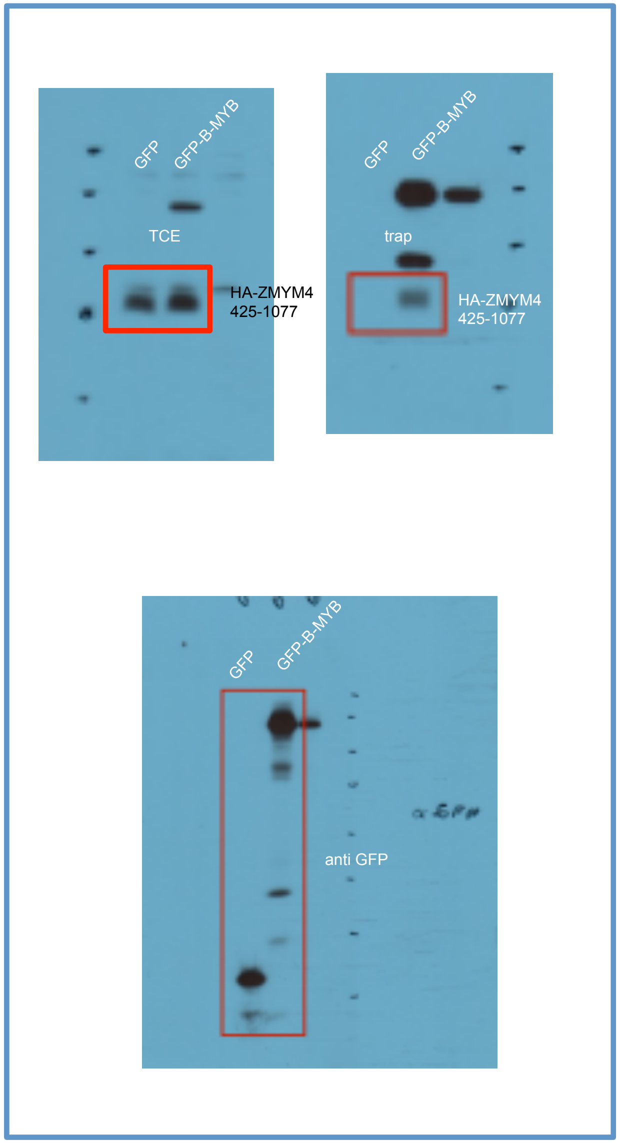

Fig.2d

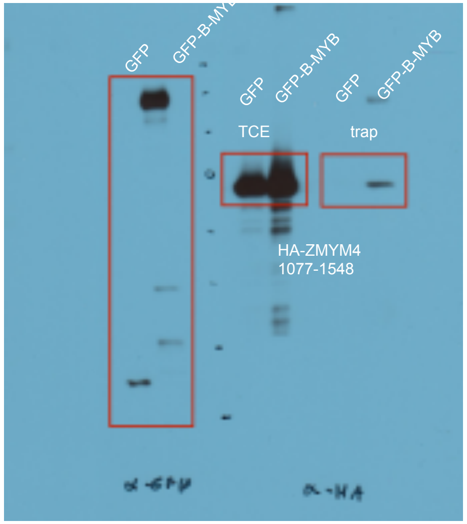

Fig.2g

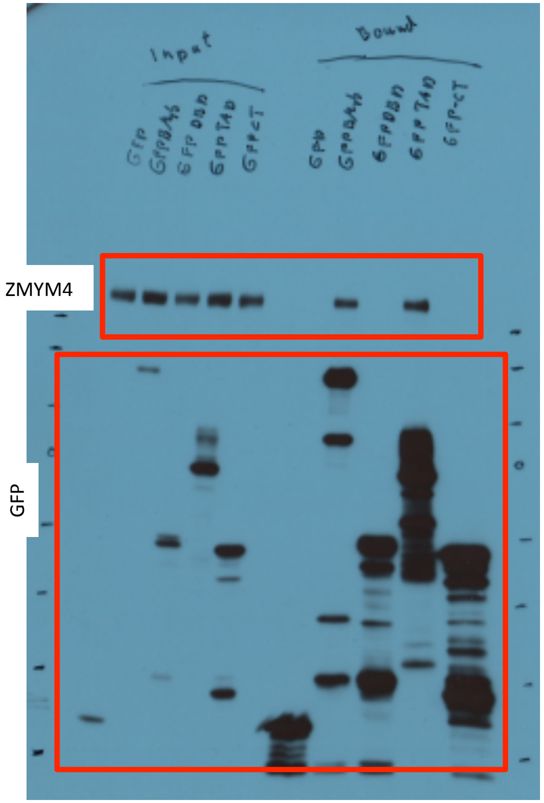

Fig.2f

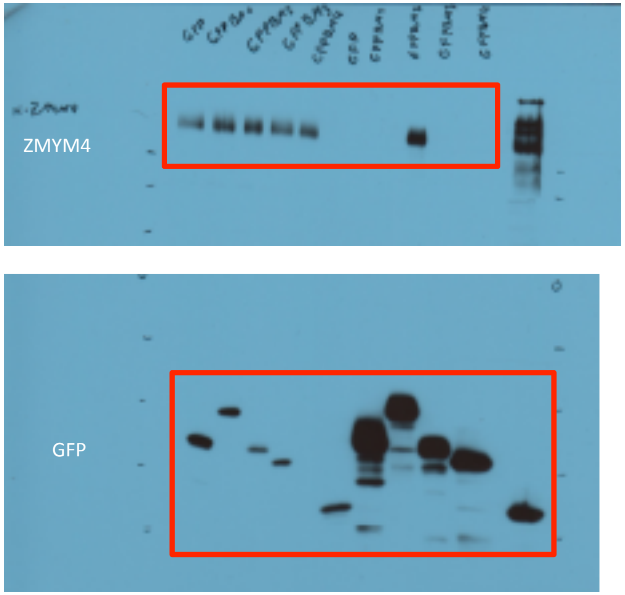

Fig.2h

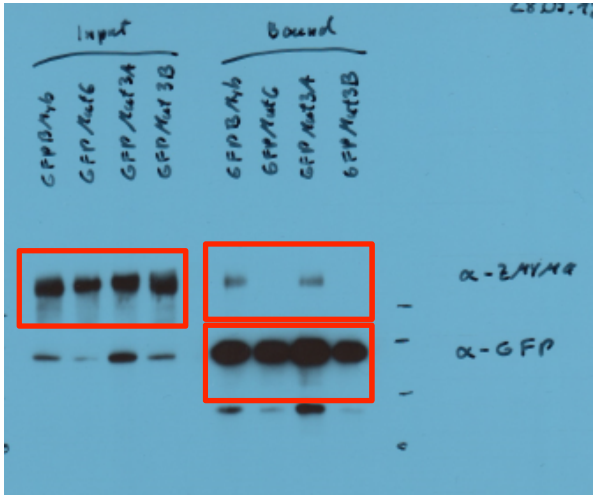

Fig.3a

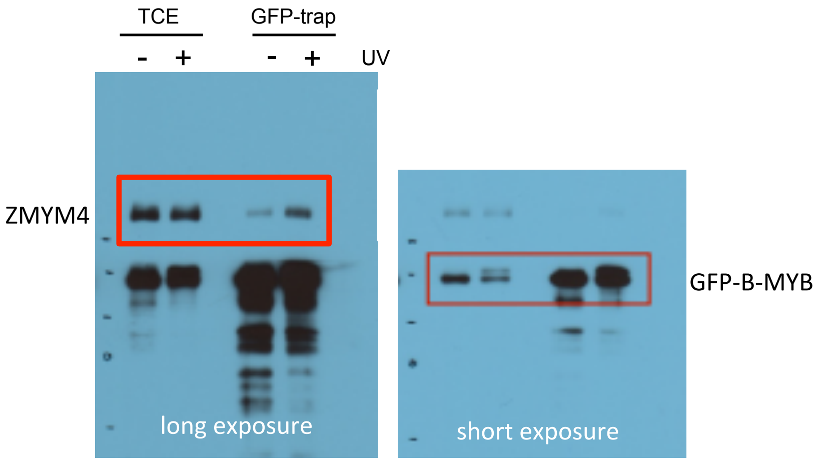
TCE
GFP-trap
- + - + UV
ZMYM4
long exposure
short exposure
GFP-B-MYB

Fig.3b

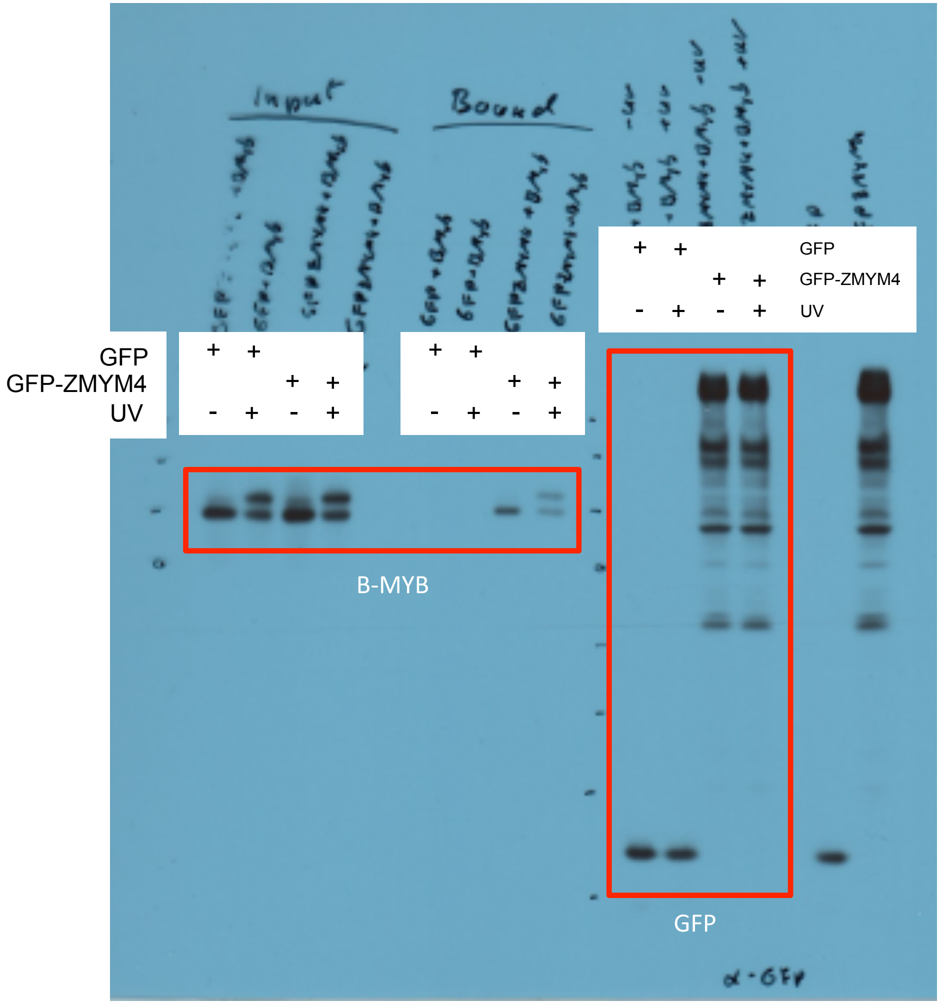
Input
Bound
GFP
GFP-ZMYM4
UV
B-MYB
GFP
α-GFP

Fig.4a

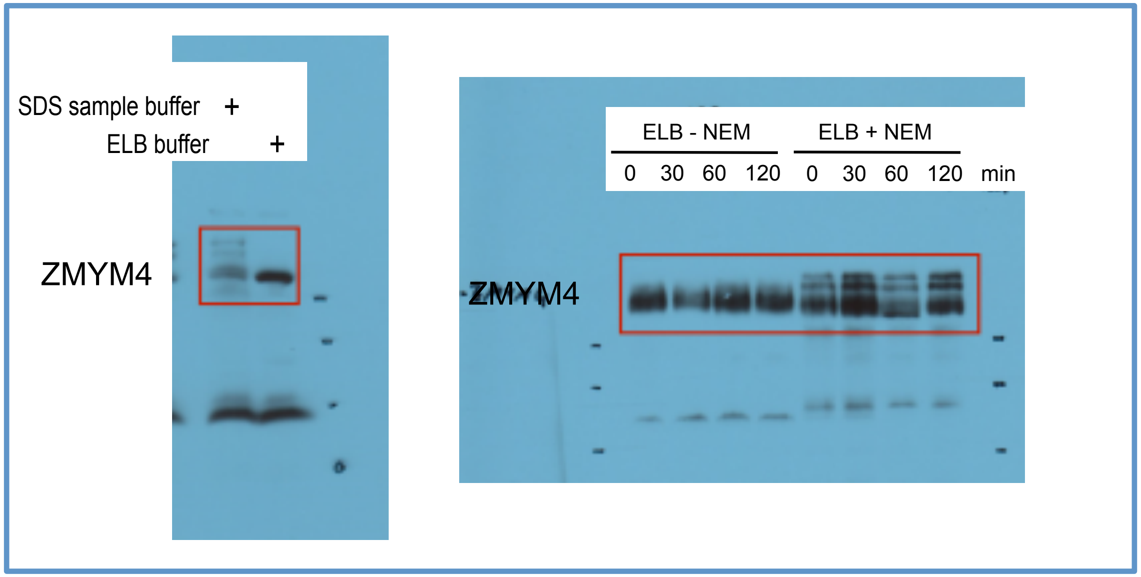

Fig.4b

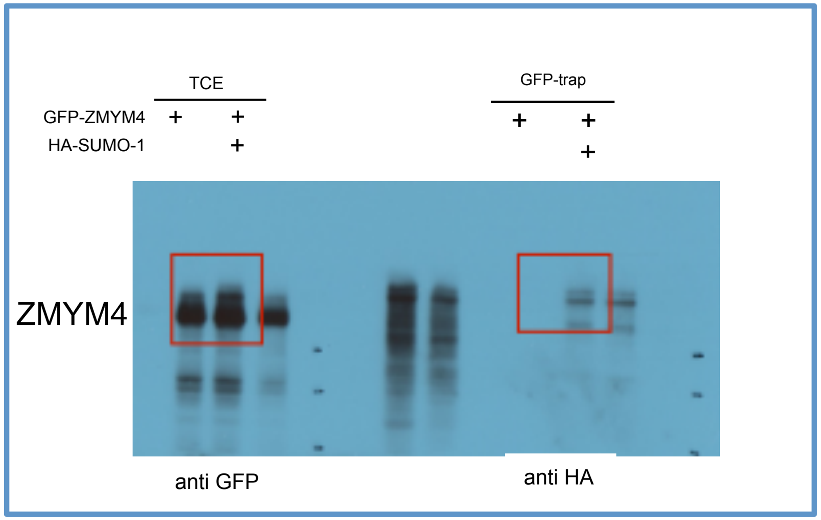

Fig.4c

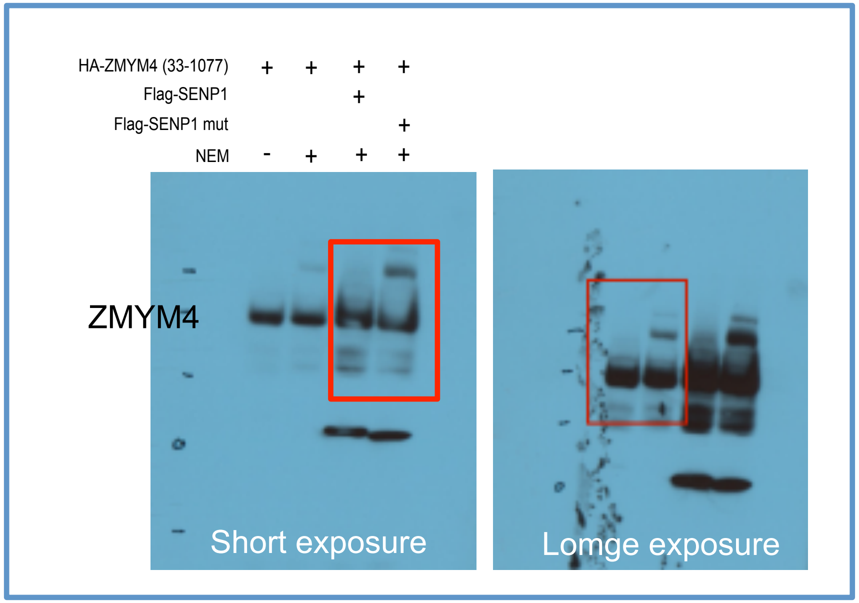

Fig.4d

Fig.4e

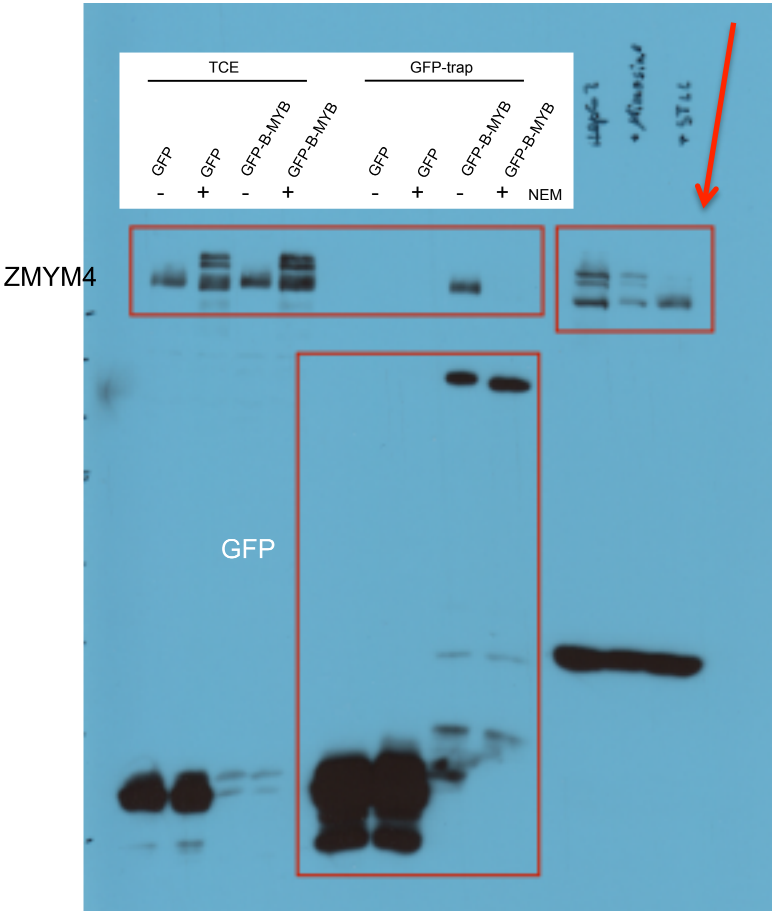

Short exposure

Fig.4d (longer exposure)

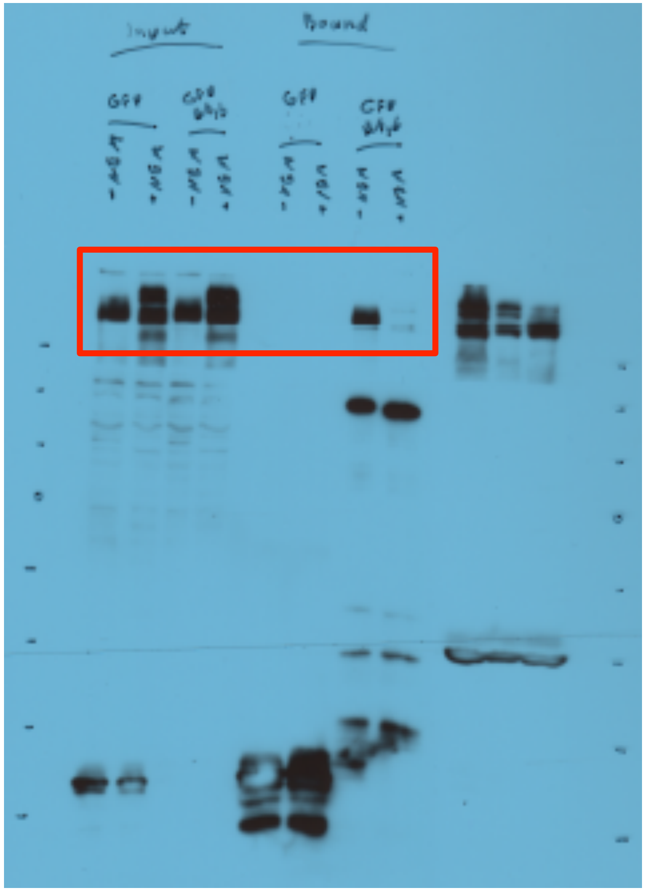

longer exposure

Fig.5a

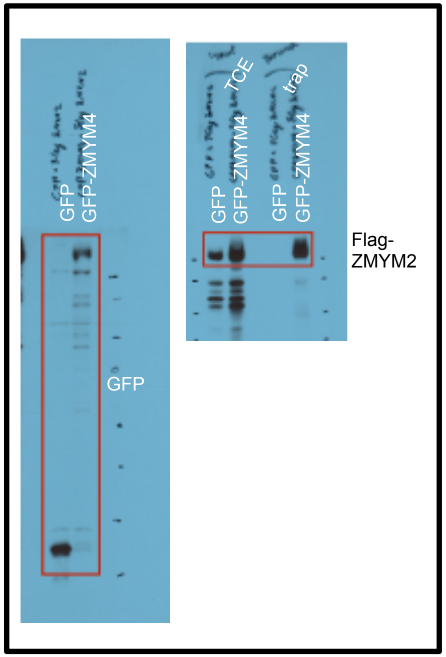

Fig.5c

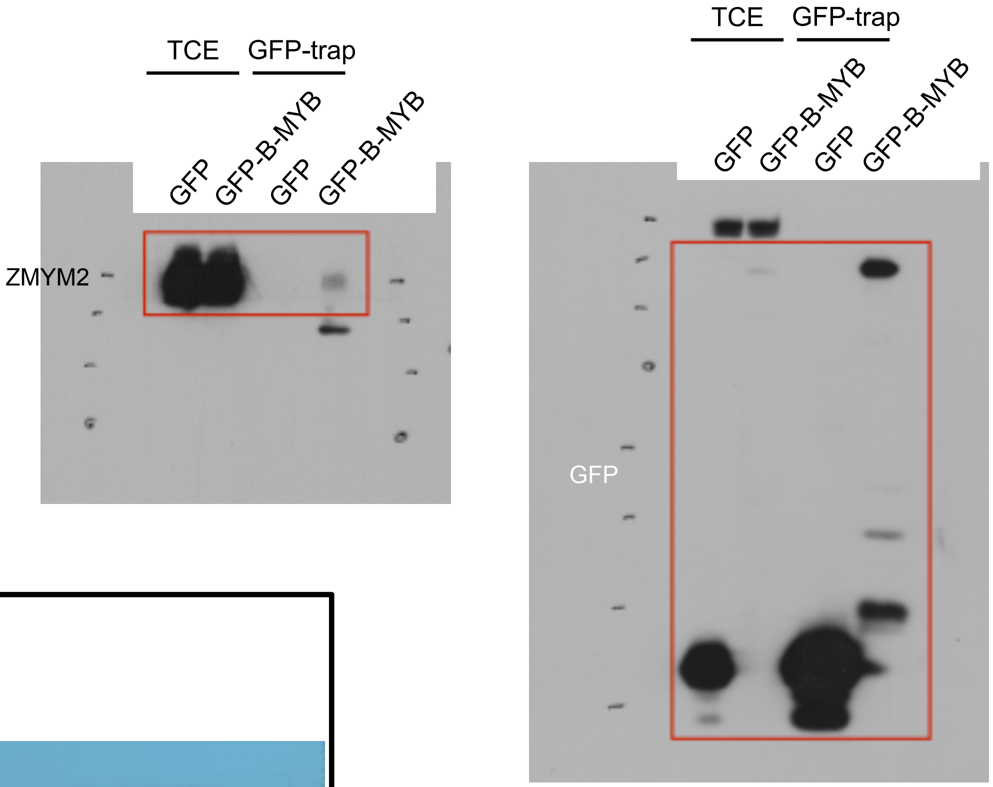

Fig.5b

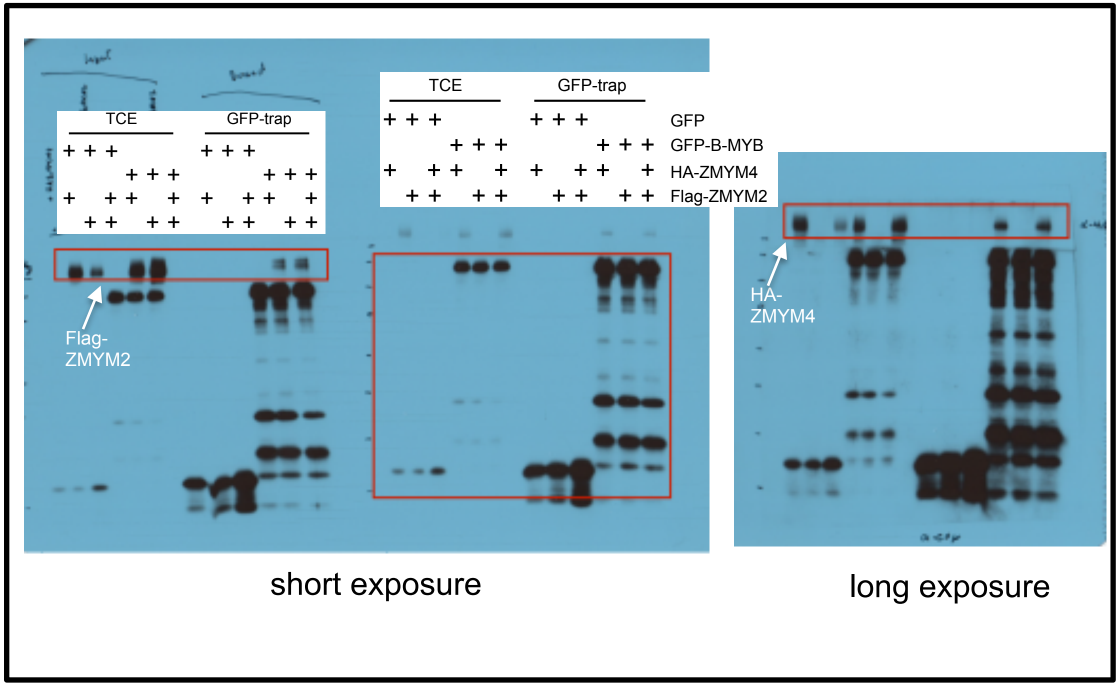

Fig.5e

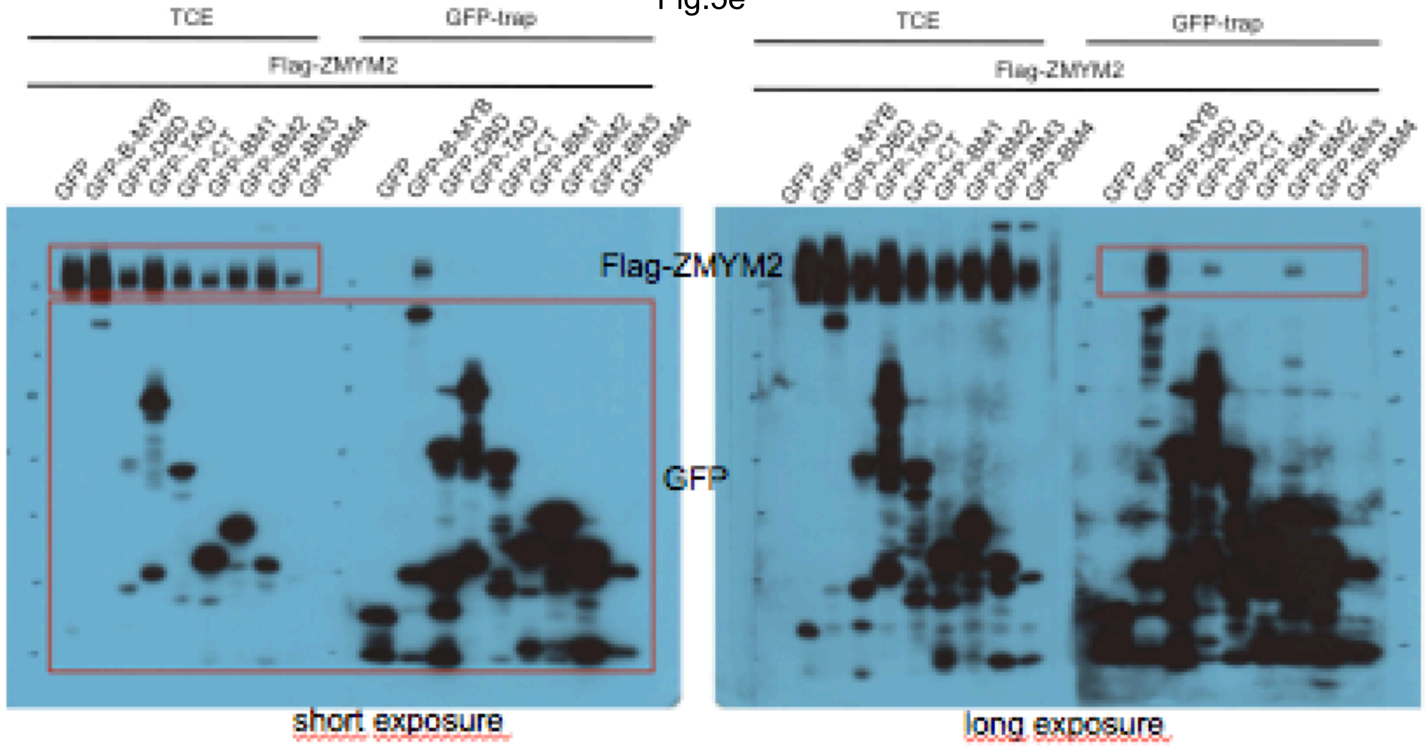

Fig5f

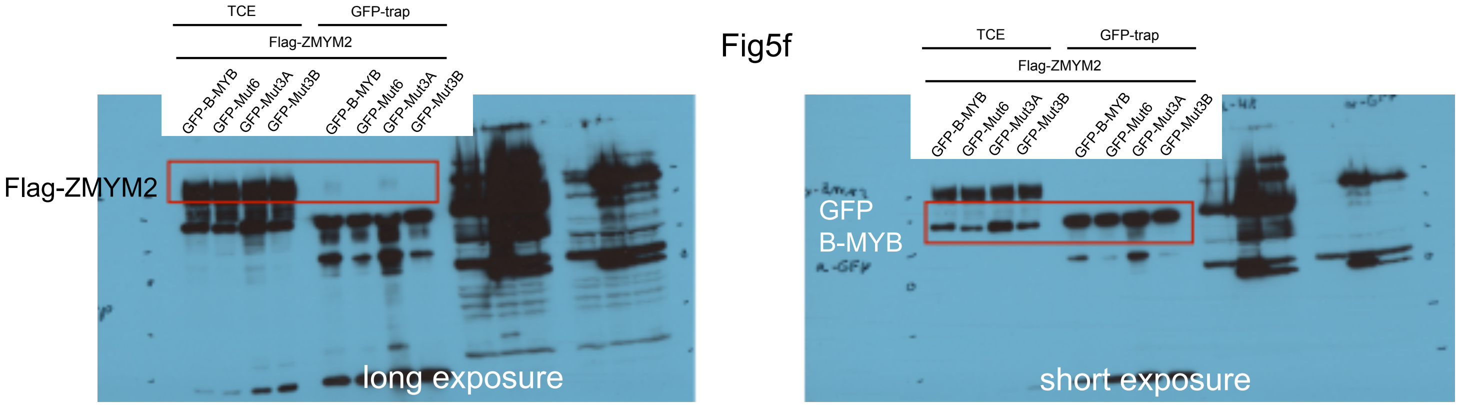

Fig.5g

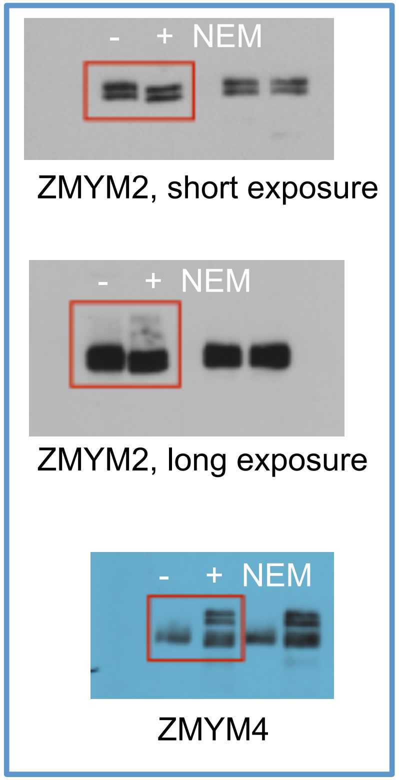

Fig.6b

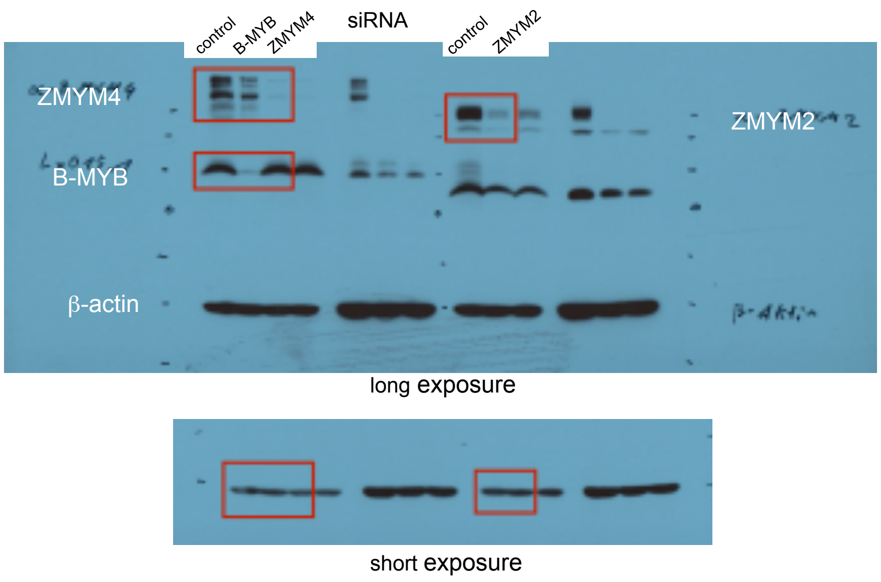

control
B-MYB
ZMYM4
siRNA
control
ZMYM2
ZMYM4
B-MYB
ZMYM2
β-actin
long exposure
short exposure

Fig.7b

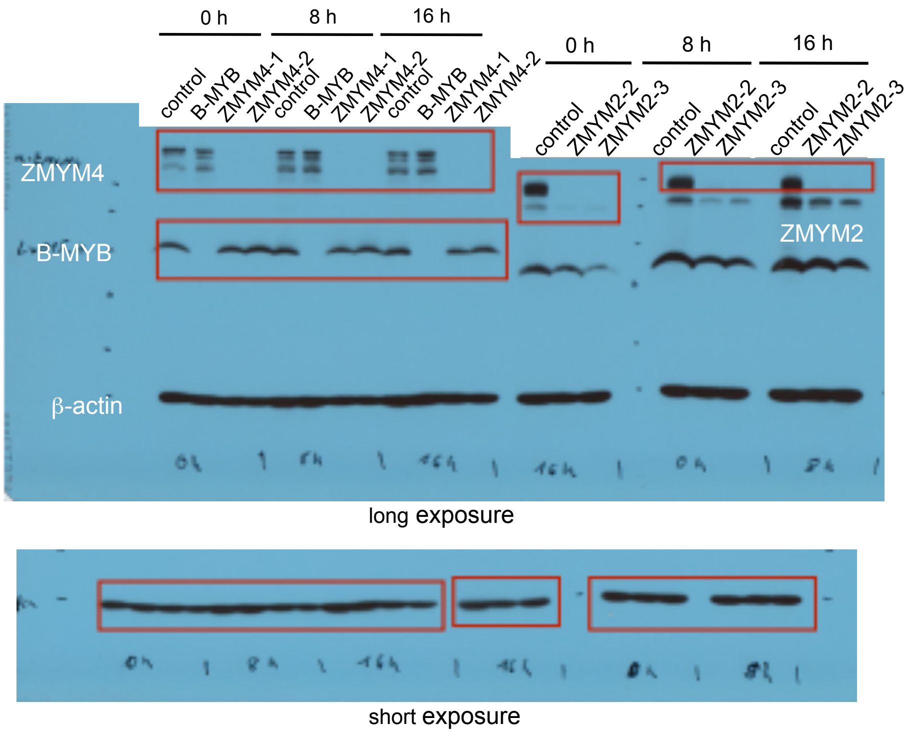

0 h
8 h
16 h
control
B-MYB
ZMYM4-1
ZMYM4-2
control
B-MYB
ZMYM4-1
ZMYM4-2
control
B-MYB
ZMYM4-1
ZMYM4-2
0 h
8 h
16 h
control
ZMYM2-2
ZMYM2-3
control
ZMYM2-2
ZMYM2-3
control
ZMYM2-2
ZMYM2-3
ZMYM4
B-MYB
ZMYM2
β-actin
long exposure
short exposure

Fig.7d

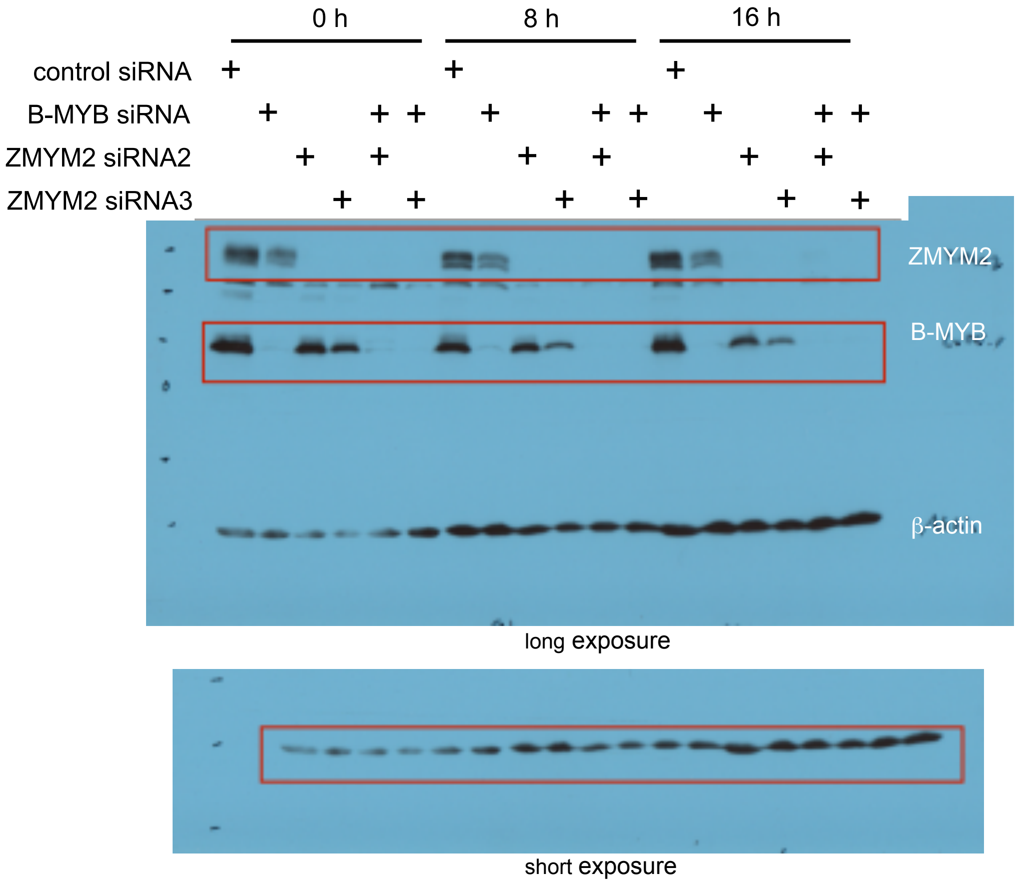
0 h
8 h
16 h
control siRNA
B-MYB siRNA
ZMYM2 siRNA2
ZMYM2 siRNA3
ZMYM2
B-MYB
β-actin
long exposure
short exposure

Fig.8b

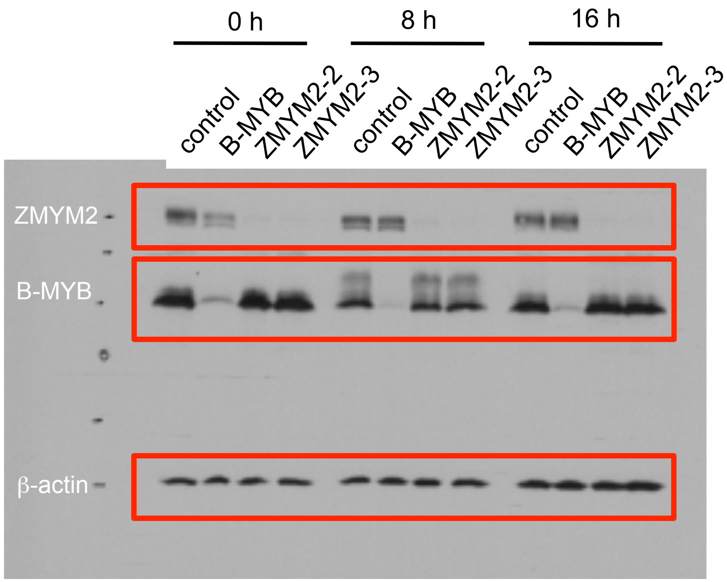
0 h
8 h
16 h
control
B-MYB
ZMYM2-2
ZMYM2-3
control
B-MYB
ZMYM2-2
ZMYM2-3
control
B-MYB
ZMYM2-2
ZMYM2-3
ZMYM2
B-MYB
β-actin
